# Status and trends of early-onset cancers and their risk factors in China: population-based study

**DOI:** 10.7189/jogh.16.04005

**Published:** 2026-01-12

**Authors:** Zhangjun Yun, Qianru Yang, Xinpu Han, Chaoran Wang, Mengchao Wang, Yuanyuan Wang, Yao Zhang, Na Wang, Lili Zhang, Fanming Kong

**Affiliations:** 1 Department of Oncology, First Teaching Hospital of Tianjin University of Traditional Chinese Medicine, Tianjin, China; 2 National Clinical Research Center for Chinese Medicine, Tianjin, China; 3 National Clinical Research Center for Chinese Medicine Acupuncture and Moxibustion, Tianjin, China; 4 Tianjin Cancer Institute of Traditional Chinese Medicine, Tianjin, China; 5 Department of Oncology and Hematology, Dongzhimen Hospital, Beijing University of Chinese Medicine, Bejing, China

## Abstract

**Background:**

This study aimed to assess the burden of early-onset cancers in China and the trends in their associated risk factors based on the latest cancer statistics.

**Methods:**

We integrated and analysed data on 34 cancers in China from the Global Burden of Disease 2021 study and the Global Cancer Observatory 2022 project. The primary outcomes included age-standardised incidence (ASIR), mortality (ASMR), and disability-adjusted life years rates (ASDR), and the average annual percent change.

**Results:**

In 2022, an estimated 743 688 new cases and 159 167 cancer-related deaths caused by early-onset cancers in China, with an ASIR of 98.37 per 100 000, and an ASMR of 21.40 per 100 000. Thyroid cancer, breast cancer, and cervical cancer were the most common cancers among female, while thyroid cancer, liver and intrahepatic bile ducts cancer, and trachea, bronchus, and lung (TBL) cancer were the most common cancers among male. Breast cancer, cervical cancer, and TBL cancer had the highest mortality rates in female, while liver and intrahepatic bile ducts cancer, TBL cancer, and colorectal cancer had the highest rates in male. From 1990–2021, the largest increases in ASIR, ASMR, and ASDR were observed for early-onset neuroblastoma and other peripheral nervous cell tumours, multiple myeloma, and kidney cancer. Smoking and high body mass index remained the primary risk factors contributing to disability-adjusted life years for most early-onset cancers.

**Conclusions:**

Developing targeted health prevention strategies for specific cancer types and promoting healthy lifestyles could help reduce the burden of early-onset cancers in China.

As a major disease that poses a serious threat to human health globally, cancer has been a key area of medical research in terms of its incidence trends and influencing factors. Although cancer is typically more prevalent in adults aged 50 and older, the incidence of early-onset cancer (<50 years) has increased globally [1]. Early-onset cancers not only affect a significant proportion of the working-age population, but also pose unique challenges in terms of prevention, diagnosis and treatment, which will significantly increase the associated disease burden [2].

In China and other developed countries, cancer was the leading cause of death [3]. During the past few decades, China has experienced rapid economic development, social transformation, and profound changes in lifestyles. These macro-level changes are closely related to the development of cancer, as they may affect individual exposure factors, health behaviours, and disease susceptibility through multiple pathways [4]. For example, environmental pollution problems brought about by accelerated industrialisation, the accelerated pace of life and changes in behavioural patterns due to urbanisation, and the shift in the dietary structure of the population from the traditional pattern to high energy and high fat intake have all played an important role in the changes in the gradual rejuvenation of the age of cancer incidence [5]. At the same time, the medical field has made great strides in diagnostic techniques and treatment methods, improving the early diagnosis rate, significantly improving the prognosis of some cancer patients and extending their survival time [6]. However, over the past few decades, cancer incidence and mortality in China continued to increase, with annual medical expenditures for malignant tumours exceeding 220 billion Renminbi (RMB) [7]. Cancer screening, early diagnosis, and early treatment were consistently emphasised as core strategies in China’s cancer prevention and control efforts. Therefore, understanding the burden and trends of early-onset cancer in China represented an urgent and essential approach to advancing the country's cancer control initiatives.

Most previous studies on the burden of early-onset cancers have focused on the global epidemiology and disease burden of multiple or a particular cancer type [2,8–11]. In contrast, the epidemiologic trends of multiple early-onset cancers have not been comprehensively studied and thoroughly investigated in China, a country with rapidly changing environment, lifestyle, and health care dynamics. Especially after the COVID-19 pandemic, cancer patients seemed to experience more severe symptoms and had a higher risk of mortality, making the trend of early-onset cancer burden even more concerning [12]. The most recent updates from the Global Cancer Observatory (GLOBOCAN) 2022 and the Global Burden of Disease (GBD) 2021 study have furnished an expanded data set, thereby enhancing the precision and breadth of cancer burden estimations [13,14]. By synthesising insights from these databases, a more comprehensive understanding of the early-onset cancer burden in China was attained, which is pivotal for formulating targeted interventions and optimising survival outcomes. Through this study, we expect to provide a key scientific basis for the development of public health policies for early-onset cancers in China that address the unique needs of the young population, optimise the allocation of health care resources, and improve patient management, which will ultimately help to mitigate the impact of early-onset cancers on the health of China's population, as well as provide unique perspectives and valuable data from China for global cancer research.

## METHODS

### Data sources

This study adopted the definition of early-onset cancer as cancer occurring between the ages 15–49, as established by two previous studies [1,2]. We extracted all available malignant tumours from the GLOBOCAN 2022 and GBD 2021 studies, classified according to the 10th edition of the International Classification of Diseases. The International Classification of Diseases codes for the cancer types in the GLOBOCAN and GBD databases are provided in Table S1 in the **Online Supplementary Document**. Notably, we included non-melanoma skin cancer (NMSC, C44) and combined colon, rectal, and anal cancers into a single colorectal cancer category (C18–C21). We accessed data from the GBD 2021 results, which are available online through the Institute for Health Metrics and Evaluation [13], as well as from the GLOBOCAN 2022 estimates provided by the International Agency for Research on Cancer, available at Cancer Today [14]. Based on GLOBOCAN 2022, we estimated the incidence and mortality rates for 34 types of early-onset cancer in China for the year 2022. Using the GBD 2021 database, we estimated the trends in incidence, mortality, disability-adjusted life years (DALYs), and the percentage contribution of risk factors for 32 early-onset cancers in China from 1990–2021. All reported age-standardised incidence rates (ASIR), age-standardised mortality rates (ASMR), and age-standardised disability-adjusted life years rates (ASDR) in this study were reported per 100 000 population.

### Estimates

Comprehensive details on the data biasources and methodologies used to compile these results can be found in the associated publications and on the online platform [13,14]. The GBD 2021 collaborators modelled the incidence and mortality estimates for both male and female individuals across all age groups. When direct incidence data are unavailable, mortality-to-incidence ratios were employed. The cause-of-death ensemble model was used to estimate cause-specific mortality by gender, age, location, and year. The GBD team developed Disease Modeling-Meta Regression (DisMod-MR) software (version 2.1, IHME, University of Washington, Seattle, WA, USA), a Bayesian meta-regression tool, to estimate incidence by analysing cascading processes. The DisMod-MR framework is designed for epidemiological modelling, particularly for synthesising disparate data sources, such as surveys, cohort studies, and clinical records, while adjusting for covariates and study-level biases. It employs hierarchical models with Markov chain Monte Carlo sampling to estimate disease parameters (such as incidence, prevalence) and their uncertainties, accounting for heterogeneity across populations and missing data. The software is widely used in global health research, including the GBD study, due to its flexibility in handling complex, sparse, or inconsistent data [13]. Prior to modelling, data points and biases were adjusted in the following ways:

(1) by decomposing previously undistributed data by age and sex

(2) by applying a Bayesian, regularised, trimmed meta-regression model to directly compare study designs and case definitions.

Information on bias correction and other modifications applied to each specific disease can be found in the GBD 2021 capstone report [13]. As a comprehensive measure of overall health loss, DALYs were calculated by summing years of life lost (YLLs) and years lived with disability (YLDs) for each aetiology. For YLLs, the values were calculated by multiplying the number of deaths attributable to a specific cause by the remaining life expectancy at the time of death, based on standard life expectancy. The GBD 2021 cause-of-death database included data from various sources, including vital registration, cause-of-death inference, cancer registries, police records, sibling history, monitoring, and surveys or census data collected since 1980. Additionally, we estimated the DALYs burden of early-onset cancer attributable to risk factors in 2021. The GBD Comparative Risk Assessment framework was used to assess exposure to disease-related risk factors and their corresponding disease burden. Effect sizes were initially estimated by quantifying the relative risks for specified health outcomes associated with exposure to identified risk factors. By considering the distribution of exposure across different ages, genders, locations, and years, and the relative risks associated with each exposure level, population-attributable scores for YLLs and years lived with disability were calculated for each risk factor. Meanwhile, the GLOBOCAN 2022 project provides estimates for 36 cancer types across 185 countries and territories, drawing on data from national and subnational cancer registries, mortality databases, and predictive models. For countries with robust data, short-term prediction models are applied, while mortality-to-incidence ratios estimates, adjusted for the Human Development Index, are used in regions with limited data. In the absence of national registries, data from neighbouring countries is used as a proxy. Additionally, non-specific or poorly defined cases are reallocated to specific cancer categories to improve the precision of the estimates. It is worth noting that the cancer data for China in GLOBOCAN 2022 were derived from sub-national registries. The incidence was calculated as the weighted average of 919 sub-national registries (2015–2017) applied to the 2022 population, while mortality was estimated from incidence using mortality: incidence ratios derived from survival.

### Statistical analysis

Using the GBD framework, 95% uncertainty intervals (UIs) for all estimates were calculated by averaging 1000 draws, with the lower and upper bounds determined by the 25th and 975th ranked values of these draws [11,13]. The study also aimed to investigate trends in the incidence, mortality, and DALYs of 32 early-onset cancers in China from 1990–2021. We used linear regression models from Joinpoint software (version 4.9.1.0, National Cancer Institute, Bethesda, MD, USA) to calculate age-specific rates and their average annual percentage changes (AAPCs), with the dependent variable as the logarithmic scale of rates and the independent variable as the year [15]. In Joinpoint regression analysis, the Empirical Quantile Method is employed to construct confidence intervals (CIs) for the AAPC. This approach involves generating multiple resampled data sets through bootstrapping, fitting the regression model to each data set, and calculating the corresponding AAPC values. The desired CI is then determined by identifying the appropriate percentiles from the distribution of these AAPC estimates. This method addresses the conservative nature of asymptotic CI for AAPC, providing a more accurate representation of uncertainty in trend analyses. The AAPC, which summarises the trend over a fixed period, was derived as the weighted average of the annual percentage changes. This method provides a single value to represent the average change across multiple years. An AAPC of 0.1, for instance, would indicate a 0.1% increase annually. Trends were assessed through AAPC values and their corresponding 95% CIs. Data analysis was conducted using *R*, version 4.4.2 (R Foundation for Statistical Computing, Vienna, Austria). The data used in this study were obtained from the publicly accessible study, which was approved by the Institutional Review Board at the University of Washington and followed the Guidelines for Accurate and Transparent Health Estimates Reporting [13]. Our research adheres to The Strengthening the Reporting of Observational studies in Epidemiology (STROBE) guidelines [16].

### Ethics

These data sets in this study have undergone rigorous privacy protection and data anonymisation processes during their collection and publication. Specifically, the data have been de-identified to ensure that individual identities cannot be traced, effectively safeguarding personal privacy. Therefore, due to the anonymised and de-identified nature of the data, no additional ethical approval was required for this research.

## RESULTS

### Burden of 34 early-onset cancers burden in China from GLOBOCAN 2022

According to GLOBOCAN 2022 estimates, China reported 0.74 million new cases of early-onset cancer in 2022, with 0.48 million in female and 0.26 million in male (**Table 1**). The country also recorded 0.16 million deaths due to early-onset cancer, with 0.07 million in females and 0.09 million in males. The age-standardised rate (ASR) for incidence and mortality were 98.37 per 100 000 and 21.4 per 100 000, respectively.

**Table 1:** The incidence and mortality for 34 early-onset cancers in China from GLOBOCAN 2022

Cancer type	Estimated new cancer cases and ASR per 100 000 people per year						Estimated cancer-related deaths and ASR per 100 000 people per year					
	**Both sex**		**Female**		**Male**		**Both sex**		**Female**		**Male**	
	**Cases, n (%)**	**ASR**	**Cases, n (%)**	**ASR**	**Cases, n (%)**	**ASR**	**Cases, n (%)**	**ASR**	**Cases, n (%)**	**ASR**	**Cases, n (%)**	**ASR**
All cancers	743 688 (100)	98.37	484 206 (100)	129.99	259 482 (100)	68.56	159 167 (100)	21.40	67 108 (100)	18.45	92 059 (100)	24.19
Bladder	5458 (0.73)	0.68	1210 (0.25)	0.32	4248 (1.64)	1.02	707 (0.44)	0.09	157 (0.23)	0.04	550 (0.60)	0.13
Brain, central nervous system	17 854 (2.40)	2.41	8442 (1.74)	2.36	9412 (3.63)	2.46	8466 (5.32)	1.14	3229 (4.81)	0.89	5237 (5.69)	1.37
Breast	111 983 (15.06)	27.99	111 983 (23.13)	27.99	0 (0)	0	12 824 (8.06)	3.18	12 824 (19.11)	3.18	0 (0)	0
Cervix uteri	46 147 (6.21)	11.56	46 147 (9.53)	11.56	0 (0)	0	9424 (5.92)	2.35	9424 (14.04)	2.35	0 (0)	0
Colorectum	43 361 (5.83)	5.36	18 649 (3.85)	4.74	24 712 (9.52)	5.96	12 684 (7.97)	1.57	5217 (7.77)	1.32	7467 (8.11)	1.81
Corpus uteri	15 899 (2.14)	3.99	15 899 (3.28)	3.99	0 (0)	0	1288 (0.81)	0.32	1288 (1.92)	0.32	0 (0)	0
Gallbladder	1412 (0.19)	0.17	860 (0.18)	0.22	552 (0.21)	0.13	844 (0.53)	0.10	514 (0.77)	0.13	330 (0.36)	0.08
Hodgkin lymphoma	1577 (0.21)	0.24	703 (0.15)	0.22	874 (0.34)	0.26	262 (0.16)	0.04	99 (0.15)	0.03	163 (0.18)	0.04
Hypopharynx	695 (0.09)	0.08	45 (0.01)	0.01	650 (0.25)	0.15	368 (0.23)	0.05	12 (0.02)	0	356 (0.39)	0.08
Kaposi sarcoma	136 (0.02)	0.02	50 (0.01)	0.01	86 (0.03)	0.02	73 (0.05)	0.01	49 (0.07)	0.02	24 (0.03)	0.01
Kidney	11 118 (1.49)	1.38	3709 (0.77)	0.95	7409 (2.86)	1.79	1430 (0.90)	0.18	468 (0.70)	0.12	962 (1.04)	0.24
Larynx	1473 (0.20)	0.18	156 (0.03)	0.04	1317 (0.51)	0.31	580 (0.36)	0.07	52 (0.08)	0.01	528 (0.57)	0.13
Leukaemia	18 030 (2.42)	2.63	7669 (1.58)	2.30	10 361 (3.99)	2.93	8414 (5.29)	1.24	3374 (5.03)	1.03	5040 (5.47)	1.43
Lip, oral cavity	4634 (0.62)	0.58	1578 (0.33)	0.41	3056 (1.18)	0.74	1417 (0.89)	0.17	379 (0.56)	0.1	1038 (1.13)	0.24
Liver and intrahepatic bile ducts	49 936 (6.71)	6.13	8054 (1.66)	2.07	41 882 (16.14)	10.00	37 240 (23.40)	4.56	5373 (8.01)	1.37	31 867 (34.62)	7.60
Melanoma of skin	1079 (0.15)	0.14	572 (0.12)	0.16	507 (0.20)	0.13	502 (0.32)	0.06	215 (0.32)	0.06	287 (0.31)	0.07
Mesothelioma	237 (0.03)	0.03	124 (0.03)	0.03	113 (0.04)	0.03	135 (0.08)	0.02	49 (0.07)	0.01	86 (0.09)	0.02
Multiple myeloma	2238 (0.30)	0.28	963 (0.20)	0.25	1275 (0.49)	0.31	1009 (0.63)	0.13	400 (0.60)	0.11	609 (0.66)	0.15
Nasopharynx	15 411 (2.07)	1.93	4460 (0.92)	1.16	10 951 (4.22)	2.66	4363 (2.74)	0.54	984 (1.47)	0.25	3379 (3.67)	0.81
Non-Hodgkin lymphoma	13 146 (1.77)	1.77	5731 (1.18)	1.57	7415 (2.86)	1.96	3777 (2.37)	0.51	1318 (1.96)	0.36	2459 (2.67)	0.65
Non-melanoma skin cancer	4364 (0.59)	0.57	2039 (0.42)	0.55	2325 (0.90)	0.59	569 (0.36)	0.07	235 (0.35)	0.06	334 (0.36)	0.08
Oesophagus	5841 (0.79)	0.71	1213 (0.25)	0.31	4628 (1.78)	1.10	3571 (2.24)	0.43	521 (0.78)	0.13	3050 (3.31)	0.72
Oropharynx	937 (0.13)	0.12	227 (0.05)	0.06	710 (0.27)	0.17	362 (0.23)	0.05	66 (0.10)	0.02	296 (0.32)	0.07
Ovary	16 010 (2.15)	4.37	16 010 (3.31)	4.37	0 (0)	0	4066 (2.55)	1.05	4066 (6.06)	1.05	0 (0)	0
Pancreas	6665 (0.90)	0.82	2495 (0.52)	0.65	4170 (1.61)	0.99	4728 (2.97)	0.58	1619 (2.41)	0.41	3109 (3.38)	0.74
Penis	695 (0.09)	0.17	0 (0)	0	695 (0.27)	0.17	171 (0.11)	0.04	0 (0)	0	171 (0.19)	0.04
Prostate	739 (0.10)	0.18	0 (0)	0	739 (0.28)	0.18	268 (0.17)	0.07	0 (0)	0	268 (0.29)	0.07
Salivary glands	2581 (0.35)	0.35	1329 (0.27)	0.38	1252 (0.48)	0.33	340 (0.21)	0.04	118 (0.18)	0.03	222 (0.24)	0.05
Stomach	22 815 (3.07)	2.82	10 225 (2.11)	2.62	12 590 (4.85)	3.02	11 404 (7.16)	1.40	4710 (7.02)	1.20	6694 (7.27)	1.60
Testis	1991 (0.27)	0.54	0 (0.00)	0	1991 (0.77)	0.54	224 (0.14)	0.06	0 (0)	0	224 (0.24)	0.06
Thyroid	251 192 (33.78)	32.54	177 724 (36.70)	47.51	73 468 (28.31)	18.52	1437 (0.90)	0.18	971 (1.45)	0.26	466 (0.51)	0.12
Trachea, bronchus and lung	67 052 (9.02)	8.21	34 958 (7.22)	8.78	32 094 (12.37)	7.67	25 988 (16.33)	3.17	9145 (13.63)	2.29	16843 (18.30)	4.01
Vagina	434 (0.06)	0.11	434 (0.09)	0.11	0 (0)	0	89 (0.06)	0.02	89 (0.13)	0.02	0 (0)	0
Vulva	548 (0.07)	0.14	548 (0.11)	0.14	0 (0)	0	143 (0.09)	0.04	143 (0.21)	0.04	0 (0)	0

ASR – age-standardised rate, GLOBOCAN – Global Cancer Observatory

**Figure 1:**
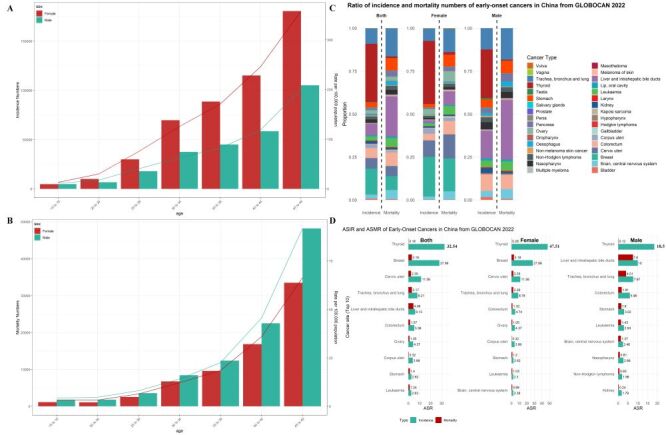
Burden of 34 early-onset cancers burden in China from GLOBOCAN 2022. **Panel A.** Global age-specific counts and rates of incidence by sex. **Panel B.** Global age-specific counts and rates of mortality by sex. **Panel C.** Proportion of incidence and mortality numbers of 34 early-onset cancers in China by sex. **Panel D.** ASIR and ASMR of the top 10 early-onset cancers in Chin. ASIR – age-standardised incidence rate, ASMR – age-standardised mortality rate, GLOBOCAN – Global Cancer Observatory.

When stratified by 5-year age groups, cancer incidence rates were higher in female across all age groups, while cancer mortality rates were higher in males in each group (**Figure 1**, Panels A–B). Thyroid cancer (33.78%), breast cancer (15.06%), trachea, bronchus, and lung (TBL) cancer (9.02%), and liver and intrahepatic bile duct (LIBD) cancer (6.71%) were the most common early-onset cancers by new case count (**Figure 1**, Panel C). Among female, the most frequent cancers were thyroid cancer (36.70%), breast cancer (23.13%), cervical cancer (9.53%), and TBL cancer (7.22%), while in males, the most common cancers were thyroid cancer (28.31%), LIBD cancer (16.14%), TBL cancer (12.37%), and colorectum cancer (9.52%). Between the ages of 15–49, the highest number of deaths were attributed to LIBD cancer (23.40%), TBL cancer (16.33%), breast cancer (8.06%), and colorectum cancer (7.97%) (**Figure 1**, Panel C). Notably, early-onset breast cancer (19.11%), cervical cancer (8.11%), TBL cancer (13.63%), and LIBD cancer (8.01%) caused the most deaths in Chinese female, while LIBD cancer (34.62%), TBL cancer (18.30%), colorectum cancer (8.01%), and stomach cancer (7.27%) accounted for the highest death toll among Chinese male.

Regarding the ASIR (**Figure 1**, Panel D), the highest rates in female were for thyroid cancer (47.51 per 100 000), breast cancer (27.99 per 100 000), cervical cancer (11.56 per 100 000), and TBL cancer (8.78 per 100 000). In male, the highest ASIR were for thyroid cancer (18.52 per 100 000), LIBD cancer (10.00 per 100 000), TBL cancer (7.67 per 100 000), and colorectum cancer (5.96 per 100 000). In terms of ASMR, early-onset LIBD cancer (7.60 per 100 000) and TBL cancer (4.01 per 100 000) had the highest ASMR in male, while breast cancer (3.18 per 100 000) and cervical cancer (2.35 per 100 000) had the highest ASMR in female.

**Figure 2:**
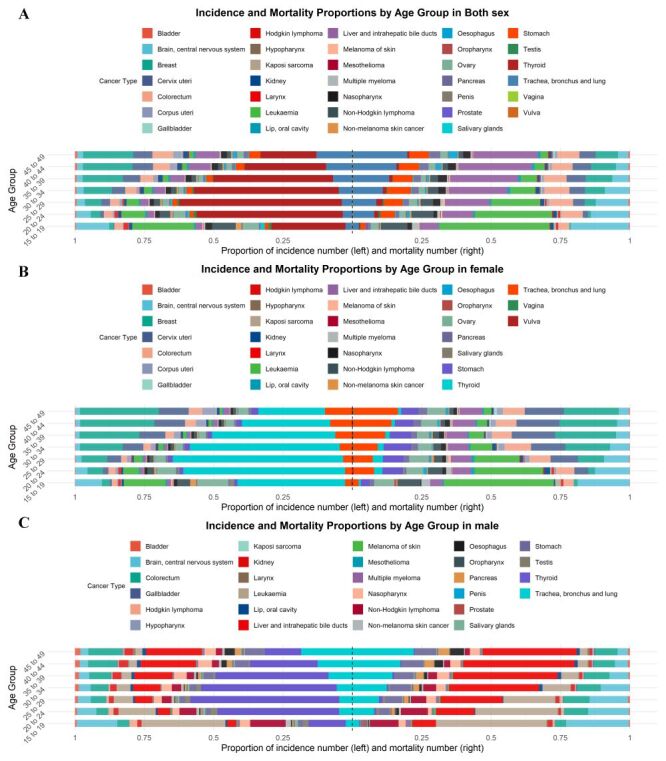
Age-specific counts and proportion of 34 cancers incidence and mortality numbers in China by sex from GLOBOCAN 2022. **Panel A.** Incidence and mortality proportions by age group in both sexes. **Panel B.** Incidence and mortality proportions by age group in female. **Panel C.** Incidence and mortality proportions by age group in male. GLOBOCAN – Global Cancer Observatory.

Significant differences were observed in the incidence and mortality proportions of various cancers across different 5-year age groups (**Figure 2**, Panel A). In the overall population, the proportion of new cases of thyroid cancer peaked at ages 25–29 (58.60%), then gradually declined. In the 15–19 (39.81%) and 20–24 (27.77%) age groups, leukaemia (39.69%) was the leading cause of cancer-related deaths. Among female aged 15–19, the most common new cancer cases were thyroid cancer (38.80%), leukaemia (15.03%), ovarian cancer (11.22%), and brain and central nervous system (CNS) cancer (9.57%) (**Figure 2**, Panel B). In contrast, among male in the same age group, leukaemia, brain and CNS cancer, and non-Hodgkin lymphoma had the highest numbers of both new cases and deaths (**Figure 2**, Panel C). From ages 15–49, the proportion of new cases and deaths from breast cancer, TBL cancer, and LIBD cancer increased steadily across all age groups. Conversely, the proportion of new cases and deaths from brain and CNS cancer decreased across these age groups. Detailed data on the incidence and mortality of 34 types of cancer in each age group, stratified by sex, are presented in Table S2–8 in the **Online Supplementary Document**.

### Trends in incidence, mortality and DALYs of 32 early-onset cancers from 1990–2021

**Figure 3:**
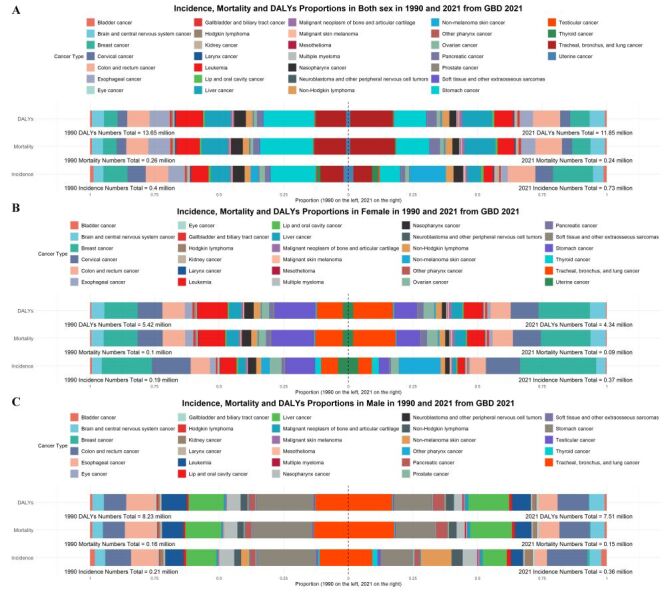
Trends in incidence, mortality and DALYs numbers for 32 cancers in China by sex from 1990–2021. **Panel A.** Incidence, mortality and DALYs proportions in both sexes in 1990 and 2021 from GBD 2021. **Panel B.** Incidence, mortality and DALYs proportions in female in 1990 and 2021 from GBD 2021. **Panel C.** Incidence, mortality and DALYs proportions in male in 1990 and 2021 from GBD 202. DALYs – disability-adjusted life years, GBD – Global Burden of Disease.

According to GBD 2021 estimates, the number of early-onset cancers in China was estimated to be 0.73 million, an increase of about 83% from 1990 (0.40 million) (**Figure 3**, Panel A). Early-onset cancers in China were estimated to cause 0.24 million deaths and 11.85 million DALYs in 2021, both of which were down from the number of mortality (0.26 million) and DALYs (13.65 million) in 1990. Table S9 in the **Online Supplementary Document** showed the changes in the number of incidences, mortality, and DALYs from 1990–2021 for 32 early-onset cancers. Briefly, non-melanoma skin cancer, multiple myeloma, neuroblastoma and other peripheral nervous cell tumours and kidney cancer were the early-onset cancers with the largest increase in the number of incidences. A dramatic increase in the number of mortality and DALYs was observed in early-onset multiple myeloma, neuroblastoma and other peripheral nervous cell tumours, kidney cancer, and mesothelioma. For female, the number of early-onset cancers in China was estimated to reach 0.37 million, an increase of about 95% from 1990 (0.19 million) (**Figure 3**, Panel B). In 2021, female early-onset cancers led to 0.85 million deaths, representing a 7.5-fold increase compared to that in 1990 (0.10 million), along with 4.34 million DALYs number. Trends observed in incidence, mortality, and DALYs number for 30 female early-onset cancers from 1990–2021 were shown in detail in Table S10 in the **Online Supplementary Document**. For early-onset cancers in male, the estimated number of new cases in China in 2021 was 0.36 million, a 71% increase from 1990 (0.21 million) (**Figure 3**, Panel C). Early-onset cancers in male in 2021 resulted in 0.15 million deaths and 7.51 million DALYs, respectively, which were both decreased from the 1990 deaths (0.16 million) and DALYs (8.23 million). Trends observed in incidence, mortality, and DALYs number for 29 male early-onset cancers from 1990–2021 were shown in detail in Table S11 in the **Online Supplementary Document**.

From 1990–2021 (**Table 2**; Figure S1 **in the** **Online Supplementary Document**, Panel A), early-onset neuroblastoma and other peripheral nervous cell tumours, multiple myeloma, and kidney cancer were observed to have the largest increase in ASIR, ASMR and ASDR. Meanwhile, early-onset malignant neoplasm of bone and articular cartilage, mesothelioma, and non-melanoma skin cancer showed increasing trends in ASIR, ASMR and ASDR. Early-onset oesophageal cancer, stomach cancer, Hodgkin lymphoma, and liver cancer obtained the most substantial decrease in ASIR, while early-onset Hodgkin lymphoma, nasopharynx cancer, oesophageal cancer, and stomach cancer had the greatest decreases in ASMR and ASDR. For female in China, the ASIR of early-onset neuroblastoma and other peripheral nervous cell tumours, non-melanoma skin cancer, malignant skin melanoma, and multiple myeloma were observed to exhibit the most significant increases (Figure S1 **in the** **Online Supplementary Document**, Panel B and Table S12 in the **Online Supplementary Document**). The ASMR and ASDR of neuroblastoma and other peripheral nervous cell tumours, multiple myeloma, non-melanoma skin cancer, mesothelioma, and malignant neoplasm of bone and articular cartilage were also observed to have the largest increases among female early-onset cancers. For male in China, the early-onset neuroblastoma and other peripheral nervous cell tumours, breast cancer, thyroid cancer, and multiple myeloma have experienced the greatest increase in ASIR over the last 30 years (Figure S1 **in the** **Online Supplementary Document**, Panel C and Table S13 in the **Online Supplementary Document**). Neuroblastoma and other peripheral nervous cell tumours, multiple myeloma, breast cancer, and kidney cancer were observed to have the highest increase in ASMR and ASDR.

**Table 2:** The ASR of incidence, mortality, and DALYs for 32 early-onset cancers in 1990 and 2021 in China, with AAPC from 1990–2021

Early-onset cancers	Incidence			Mortality			DALYs		
	**ASR (95% UI), 1990**	**ASR (95% UI), 2021**	**AAPC (95% CI)**	**ASR (95% UI), 1990**	**ASR (95% UI), 2021**	**AAPC (95% CI)**	**ASR (95% UI), 1990**	**ASR (95% UI), 2021**	**AAPC (95% CI)**
Bladder cancer	0.92 (0.59, 1.13)	1.10 (0.85, 1.42)	0.46 (0.23, 0.69)	0.35 (0.23, 0.43)	0.19 (0.15, 0.25)	−2.35 (−2.60, −2.09)	17.61 (11.38, 21.40)	10.00 (7.79, 12.95)	−2.20 (−2.47, −1.93)
Brain and central nervous system cancer	2.53 (1.79, 3.23)	4.21 (3.20, 5.54)	1.67 (1.57, 1.76)	1.88 (1.32, 2.41)	1.79 (1.35, 2.36)	−0.36 (−0.45, −0.27)	102.29 (72.04, 130.83)	98.57 (74.18, 129.83)	−0.31 (−0.41, −0.22)
Breast cancer	6.69 (5.28, 8.26)	13.69 (10.21, 17.84)	2.35 (2.26, 2.45)	2.48 (1.96, 3.07)	2.01 (1.50, 2.62)	−1.04 (−1.16, −0.91)	125.87 (99.31, 156.15)	107.04 (79.61, 140.04)	−0.87 (−0.98, −0.76)
Cervical cancer	4.90 (3.79, 6.23)	6.20 (4.39, 8.24)	1.50 (1.25, 1.75)	1.80 (1.40, 2.28)	1.11 (0.79, 1.48)	−1.10 (−1.29, −0.91)	90.81 (70.69, 115.34)	57.17 (40.63, 76.40)	−1.04 (−1.23, −0.86)
Colon and rectum cancer	6.19 (5.12, 7.26)	9.67 (7.67, 11.95)	1.32 (1.01, 1.62)	4.00 (3.30, 4.69)	3.00 (2.37, 3.74)	−1.30 (−1.54, −1.06)	203.47 (167.28, 238.56)	155.82 (123.35, 193.47)	−1.26 (−1.51, −1.00)
Oesophageal cancer	4.86 (3.98, 5.84)	2.18 (1.69, 2.76)	−3.38 (−3.63, −3.12)	4.29 (3.53, 5.16)	1.49 (1.15, 1.92)	−4.22 (−4.52, −3.91)	201.40 (165.67, 242.21)	70.61 (54.32, 90.48)	−4.23 (−4.53, −3.92)
Eye cancer	0.05 (0.03, 0.08)	0.07 (0.04, 0.11)	1.55 (1.31, 1.78)	0.01 (0.01, 0.02)	0.01 (0.00, 0.01)	−0.98 (−1.15, −0.80)	0.59 (0.33, 0.85)	0.43 (0.23, 0.60)	−0.72 (−0.90, −0.54)
Gallbladder and biliary tract cancer	0.38 (0.25, 0.46)	0.47 (0.29, 0.62)	0.88 (0.67, 1.09)	0.32 (0.22, 0.39)	0.23 (0.15, 0.30)	−1.00 (−1.09, −0.91)	15.47 (10.42, 18.92)	11.25 (7.31, 14.81)	−0.98 (−1.07, −0.88)
Hodgkin lymphoma	0.28 (0.12, 0.40)	0.18 (0.12, 0.26)	−1.48 (−1.81, −1.14)	0.24 (0.10, 0.33)	0.06 (0.04, 0.09)	−4.72 (−4.97, −4.46)	13.34 (5.85, 18.65)	3.54 (2.35, 5.28)	−4.67 (−4.94, −4.41)
Kidney cancer	0.67 (0.56, 0.78)	1.67 (1.33, 2.08)	4.02 (3.65, 4.38)	0.26 (0.22, 0.31)	0.31 (0.25, 0.39)	1.27 (0.97, 1.57)	13.42 (11.29, 15.79)	16.48 (13.07, 20.62)	1.35 (1.05, 1.65)
Larynx cancer	0.36 (0.28, 0.43)	0.33 (0.25, 0.42)	−0.30 (−0.40, −0.20)	0.26 (0.20, 0.31)	0.12 (0.09, 0.16)	−2.54 (−2.63, −2.45)	12.33 (9.64, 14.83)	6.08 (4.64, 7.76)	−2.49 (−2.58, −2.40)
Leukaemia	4.19 (3.09, 5.04)	4.03 (2.78, 5.14)	−0.18 (−0.28, −0.08)	3.71 (2.74, 4.45)	2.28 (1.61, 2.89)	−1.77 (−1.88, −1.66)	215.76 (159.60, 259.00)	133.64 (94.18, 169.17)	−1.75 (−1.86, −1.64)
Lip and oral cavity cancer	0.58 (0.48, 0.67)	0.93 (0.74, 1.16)	1.97 (1.65, 2.28)	0.29 (0.25, 0.35)	0.25 (0.19, 0.31)	−0.36 (−0.58, −0.14)	14.71 (12.30, 17.27)	12.57 (9.88, 15.77)	−0.32 (−0.54, −0.10)
Liver cancer	5.47 (4.42, 6.67)	4.63 (3.53, 6.04)	−1.09 (−1.49, −0.70)	4.96 (4.01, 6.05)	3.38 (2.58, 4.42)	−1.83 (−2.21, −1.45)	244.35 (197.98, 298.15)	166.86 (127.38, 218.12)	−1.88 (−2.27, −1.50)
Malignant neoplasm of bone and articular cartilage	0.33 (0.21, 0.61)	0.80 (0.49, 1.08)	3.12 (2.42, 3.83)	0.25 (0.16, 0.46)	0.36 (0.23, 0.49)	1.14 (0.44, 1.84)	14.74 (9.20, 26.96)	21.26 (13.53, 28.55)	1.10 (0.42, 1.78)
Malignant skin melanoma	0.21 (0.12, 0.27)	0.49 (0.26, 0.68)	3.20 (2.82, 3.58)	0.14 (0.08, 0.18)	0.12 (0.06, 0.16)	−0.45 (−0.63, −0.28)	7.23 (4.07, 9.25)	6.24 (3.39, 8.47)	−0.35 (−0.53, −0.17)
Mesothelioma	0.06 (0.05, 0.07)	0.07 (0.05, 0.09)	1.11 (0.78, 1.43)	0.05 (0.04, 0.07)	0.06 (0.05, 0.07)	1.00 (0.68, 1.32)	2.70 (2.22, 3.29)	3.00 (2.41, 3.73)	0.96 (0.65, 1.27)
Multiple myeloma	0.05 (0.04, 0.10)	0.24 (0.13, 0.33)	4.26 (3.75, 4.77)	0.04 (0.03, 0.08)	0.14 (0.08, 0.19)	2.99 (2.44, 3.53)	2.17 (1.52, 4.16)	7.46 (4.11, 10.14)	3.10 (2.55, 3.65)
Nasopharynx cancer	3.18 (2.63, 3.83)	3.46 (2.63, 4.51)	−0.59 (−1.13, −0.04)	2.13 (1.77, 2.54)	0.75 (0.58, 0.95)	−4.41 (−4.84, −3.98)	108.22 (89.91, 128.87)	39.24 (30.24, 49.79)	−4.35 (−4.80, −3.90)
Neuroblastoma and other peripheral nervous cell tumours	0.01 (0.01, 0.02)	0.06 (0.04, 0.08)	5.66 (5.31, 6.00)	0.01 (0.01, 0.01)	0.03 (0.02, 0.04)	4.60 (4.29, 4.91)	0.51 (0.37, 0.70)	1.61 (1.23, 2.03)	4.56 (4.25, 4.86)
Non-Hodgkin lymphoma	1.54 (1.28, 1.88)	2.80 (2.11, 3.51)	2.02 (1.61, 2.44)	1.05 (0.87, 1.27)	0.82 (0.63, 1.01)	−0.88 (−1.18, −0.57)	57.02 (47.32, 69.08)	45.71 (34.99, 56.43)	−0.82 (−1.14, −0.51)
Non-melanoma skin cancer	2.13 (1.33, 3.18)	12.60 (8.34, 18.17)	3.66 (2.88, 4.46)	0.13 (0.11, 0.17)	0.13 (0.10, 0.17)	0.30 (0.08, 0.52)	6.80 (5.49, 8.53)	6.78 (5.12, 8.51)	0.32 (0.10, 0.54)
Other pharynx cancer	0.16 (0.13, 0.20)	0.17 (0.13, 0.22)	−0.23 (−0.73, 0.29)	0.12 (0.09, 0.15)	0.06 (0.04, 0.07)	−3.04 (−3.48, −2.59)	5.57 (4.39, 6.88)	2.80 (2.15, 3.58)	−2.97 (−3.41, −2.52)
Ovarian cancer	1.44 (0.86, 1.96)	1.47 (1.03, 2.02)	−0.12 (−0.22, −0.01)	0.52 (0.32, 0.71)	0.43 (0.30, 0.59)	−0.97 (−1.09, −0.85)	26.65 (16.10, 36.39)	21.92 (15.46, 30.20)	−0.95 (−1.08, −0.83)
Pancreatic cancer	1.15 (0.95, 1.37)	1.23 (0.95, 1.55)	0.09 (−0.04, 0.23)	1.05 (0.87, 1.25)	1.06 (0.82, 1.33)	−0.11 (−0.24, 0.01)	50.77 (42.22, 60.69)	51.33 (39.76, 64.39)	−0.13 (−0.26, 0.00)
Prostate cancer	0.12 (0.06, 0.18)	0.28 (0.19, 0.40)	2.76 (2.55, 2.96)	0.06 (0.03, 0.09)	0.05 (0.03, 0.06)	−1.36 (−1.64, −1.08)	3.08 (1.38, 4.34)	2.42 (1.55, 3.38)	−1.13 (−1.43, −0.83)
Soft tissue and other extraosseous sarcomas	0.27 (0.18, 0.36)	0.21 (0.14, 0.32)	−0.97 (−1.08, −0.86)	0.15 (0.10, 0.20)	0.07 (0.05, 0.11)	−2.52 (−2.67, −2.37)	8.01 (5.51, 10.77)	4.07 (2.78, 6.16)	−2.51 (−2.67, −2.36)
Stomach cancer	12.87 (10.14, 15.20)	7.05 (5.46, 9.01)	−2.14 (−2.25, −2.03)	10.05 (7.93, 11.88)	3.66 (2.84, 4.69)	−3.53 (−3.69, −3.37)	487.67 (386.09, 576.02)	180.20 (139.85, 230.42)	−3.50 (−3.65, −3.33)
Testicular cancer	0.21 (0.17, 0.26)	0.62 (0.46, 0.83)	3.06 (2.67, 3.46)	0.11 (0.08, 0.13)	0.06 (0.05, 0.08)	−2.57 (−2.99, −2.16)	6.12 (4.90, 7.54)	3.98 (3.01, 5.08)	−2.32 (−2.76, −1.89)
Thyroid cancer	0.95 (0.72, 1.18)	2.20 (1.71, 2.92)	3.11 (2.87, 3.34)	0.11 (0.09, 0.13)	0.08 (0.06, 0.10)	−0.90 (−1.04, −0.75)	6.04 (4.73, 7.47)	5.27 (4.04, 6.84)	−0.40 (−0.56, −0.24)
Tracheal bronchus and lung cancer	6.65 (5.60, 7.77)	6.23 (4.88, 7.70)	−0.41 (−0.51, −0.31)	6.03 (5.08, 7.03)	4.82 (3.75, 5.97)	−0.96 (−1.10, −0.82)	291.59 (245.79, 340.24)	233.09 (181.95, 288.34)	−0.99 (−1.12, −0.85)
Uterine cancer	1.38 (0.78, 1.86)	1.71 (1.15, 2.44)	0.68 (0.24, 1.12)	0.41 (0.22, 0.56)	0.19 (0.13, 0.27)	−2.81 (−3.21, −2.41)	20.65 (11.20, 28.02)	10.16 (6.82, 14.36)	−2.62 (−3.01, −2.22)

AAPC – average annual percentage changes, ASR – age-standardised rate, CI – confidence interval, DALYs – disability-adjusted life years, UI – uncertainty interval

Overall, from 1990–2021, cancer incidence rates among female in China consistently exceeded those among male across all age groups (Figure S2 in the **Online Supplementary Document**). However, mortality and DALYs rates remained lower in female compared to male. From 1990–2021, mortality and DALYs rates across all age groups and both genders exhibited a marked declining trend, with the most substantial reductions observed in the 40–44 and 45–49 age groups. Notably, during the same period, incidence rate demonstrated a consistent upward trend across all age groups, particularly from 2005–2010. This increase was predominantly attributed to a significant rise in cancer incidence among female. In male, incidence, mortality, and DALYs rates increased progressively with age, reaching their peak in the 45–49 age group. Conversely, in female, the highest cancer incidence was observed in the 40–44 age group, followed by the 35–39 and 45–49 age groups. These findings underscored a younger age of cancer onset among female compared to male in China.

### Disability-adjusted life years from early-onset cancers attributable to risk factors

We identified 34 risk factors in GBD 2021, mainly including the percentage contribution of environmental, occupational, behavioural and metabolic risks to 23 early-onset cancer DALYs. Specifically, for environmental and occupational risk factors (Table S14 in the **Online Supplementary Document**), ambient particulate matter pollutions (20.51%; 95% UI = 11.66, 28.77) were the most frequent risk factor contributing to DALYs for TBL cancer in female. While Occupational exposure to asbestos contributed the most to DALYs caused by mesothelioma, both in male (48.11%; 95% UI = 32.18, 63.16) and female (31.11%; 95% UI = 13.73, 47.71).

For behavioural and metabolic risks (Table S15 in the **Online Supplementary Document**), the high body-mass index (BMI) was higher for kidney cancer (15.36%; 95% UI = 5.87, 25.13), thyroid cancer (11.61%; 95% UI = 8.70, 14.77), gallbladder and biliary tract cancer (11.48%; 95% UI = 7.96, 15.66), leukaemia (7.01%; 95% UI = 5.19, 8.94), multiple myeloma (5.67%; 95% UI = −1.78, 14.75), and non-Hodgkin lymphoma (3.98%; 95% UI = 1.33, 6.73) had the largest percentage of DALYs contributing, both male and female. In addition, the high BMI contributed the most DALYs for two female-specific cancers, such as uterine cancer (25.10%; 95% UI = 7.8, 34.12) and ovarian cancer (5.94%; 95% UI = 1.06, 11.62). Smoking contributed the most to DALYs for TBL cancer (67.36%; 95% UI = 63.95, 70.72), other pharynx cancer (51.97%; 95% UI = 44.02, 59.45), oesophageal cancer (36.04%; 95% UI = 29.55, 42.40), bladder cancer (32.82%; 95% UI = 29.06, 36.61), pancreatic cancer (26.99%; 95% UI = 24.82, 29.31), liver cancer (16.67%; 95% UI = 5.87, 26.97), stomach cancer (13.49%; 95% UI = 11.28, 15.72), and prostate cancer (8.26%; 95% UI = 3.83, 12.54) in male. In addition, larynx cancer (66.33%; 95% UI = 59.76, 71.32) predominantly DALYs were also caused by smoking, both in male (76.18%; 95% UI = 71.80, 80.09) and female (11.15%; 95% UI = 7.64, 15.69). High fasting plasma glucose (HFPG) (11.19%; 95% UI = 7.64, 15.69), diet high in sodium (8.11%; 95% UI = −0.00, 40.42), and drug use (11.81%; 95% UI = 7.90, 15.74) had a significant impact on the risk of pancreatic cancer, stomach cancer, and liver cancer in female respectively. For bladder cancer in female, DALYs were mainly attributed to HFSG (2.20%; 95% UI = −0.30, 4.84) whereas in male it was mainly attributed to smoking (32.82%; 95% UI = 29.06, 36.61). Diet high in red meat contributed the most DALYs to breast cancer (13.55%; 95% UI = −0.01, 28.62), both in male and female. Notably, the DALYs for cervical cancer were all due to unsafe sex (100.00%; 95% UI = 100.00, 100.00). Diet low in milk (22.4%; 95% UI = 6.24, 35.65) and diet low in whole grains (17.80%; 95% UI = 7.48, 26.43) contributed the most to the DALYs of colon and rectum cancer in female and male, respectively. Alcohol use contributed a significant proportion of DALYs for other pharynx cancer (4.39%; 95% UI = 2.56, 6.76) and oesophageal cancer (2.41%; 95% UI = 1.32, 3.80) in female. Similarly, alcohol use was also the most important risk factor for DALYs causing lip and oral cavity cancer (32.79%; 95% UI = 26.57, 39.01) and nasopharynx cancer (32.47%; 95% UI = 24.61, 40.01) in both male and female.

### The trend of risk factors of early-onset cancers burden from 1990–2021

We analysed the trend of risk factors of early-onset cancers burden from 1990–2021, separately by all gender, female and male (Table S16 in the **Online Supplementary Document**). For all gender, HFPG was the fastest growing risk factor contributing to DALYs for breast cancer and pancreatic cancer. High BMI was the fastest growing risk factor contributing to DALYs for colon and rectum cancer, gallbladder and biliary tract cancer, kidney cancer, leukaemia, liver cancer, multiple myeloma, non-Hodgkin lymphoma and thyroid cancer. Meanwhile, diet high in processed meat, alcohol use and HFPG are also the risk factors that contribute to the increasing trend of DALYs for colon and rectum cancer. Occupational exposure to trichloroethylene and smoking are also the risk factors that contribute to the increasing trend of DALYs for kidney cancer. Alcohol use is the fastest growing risk factor for lip and oral cavity cancer. Occupational exposure to trichloroethylene and smoking were also risk factors for the increasing trend of DALYs in kidney cancer. Alcohol use was the fastest growing risk factor for lip and oral cavity cancer. Simultaneously, chewing tobacco and smoking were the risk factors with increasing trend of contributing to DALYs for lip and oral cavity cancer. Occupational exposure to asbestos was the fastest growing risk factor contributing to DALYs for mesothelioma. Ambient particulate matter pollution was the fastest growing risk factor contributing to DALYs for TBL cancer. Occupational exposure to asbestos, HFPG, chromium, diesel engine exhaust and polycyclic aromatic hydrocarbons were also the risk factors that showed an increasing trend in contributing to DALYs for TBL cancer.

In female, HFPG was the fastest growing risk factor contributing to DALYs for breast cancer and pancreatic cancer. High BMI was the fastest growing risk factor contributing to DALYs for colon and rectum cancer, gallbladder and biliary tract cancer, kidney cancer, liver cancer, multiple myeloma, non-Hodgkin lymphoma, ovarian cancer, and uterine cancer. Diet high in processed meat was also a risk factor contributing to the increasing trend of DALYs for colon and rectum cancer. Occupational exposure to trichloroethylene and smoking were also risk factors contributing to the increasing trend of DALYs for kidney cancer. Occupational exposure to asbestos was the fastest growing risk factor contributing to DALYs for mesothelioma. Ambient particulate matter pollution was the fastest growing risk factor contributing to DALYs of TBL cancer. Meanwhile, HFPG, occupational exposure to arsenic, cadmium, chromium, diesel engine exhaust and polycyclic aromatic hydrocarbons were also the DALYs contributing to TBL cancer risk factors that showed an increasing trend.

In male, alcohol use was the fastest growing risk factor contributing to DALYs for breast cancer. Diet high in red meat and second-hand smoke were also risk factors contributing to increasing DALYs for breast cancer. High BMI was the fastest growing risk factor for colon and rectum cancer, gallbladder and biliary tract cancer, kidney cancer, leukaemia, liver cancer, multiple myeloma, non-Hodgkin lymphoma, and thyroid cancer with the fastest growing risk factors for DALYs. Diet high in processed meat, HFPG, and low physical activity are risk factors that contribute to the increasing trend of DALYs for colon and rectum cancer. The increasing trend of DALYs for liver cancer is also influenced by HFPG as a risk factor. Occupational exposure to trichloroethylene and smoking were also risk factors that contributed to the increasing trend of DALYs in kidney cancer. Chewing tobacco was the fastest growing risk factor contributing to DALYs for lip and oral cavity cancer. Alcohol use and Smoking were risk factors that also showed an increasing trend in their contribution to DALYs for lip and oral cavity cancer. Occupational exposure to asbestos was the fastest growing risk factor for DALYs of mesothelioma. Ambient particulate matter pollution was the fastest growing risk factor for DALYs of TBL cancer. Meanwhile, HFPG, occupational exposure to asbestos, chromium and polycyclic aromatic hydrocarbons were also the risk factors that showed an increasing trend in the effect of DALYs for TBL cancer.

## DISCUSSION

### Key findings in context

In this study, we conducted a comprehensive epidemiological assessment of the incidence, mortality, DALYs, and risk factors for early-onset cancers in China using the latest data from GLOBOCAN 2022 and GBD 2021. To our knowledge, this is the first study to integrate these two recent cancer statistics databases to systematically evaluate the disease burden and trend changes of early-onset cancers in China. Our research updates and expands the epidemiological evidence on early-onset cancers in China, offering new perspectives and insights. Most importantly, we confirmed significant differences between this specific population and the overall cancer burden in China, identifying trends in the incidence, mortality, DALYs, and risk factors for age-specific cancers in recent years.

### Public health implications

We observed that among individuals aged 15–49, the incidence of cancer was lower in male than in female, yet their mortality rate was higher, which aligns with findings from two recent large-scale epidemiological studies in China [7,17]. Prior to the age of 55, the incidence of cancer in male was lower than in female, but it increased significantly after 55 [17]. In the 15–49 age group, male mortality rates were consistently higher than those in female. These differences may be attributed to biological factors and environmental influences, including higher exposure to occupational risk factors, greater social stress, and unhealthy lifestyles in male [18,19]. We also found that thyroid cancer has become the most common early-onset cancer in both male and female in China. The increasing incidence of thyroid cancer in both sexes may be partly attributable to improvements in imaging and diagnostic technologies that facilitate the detection of small or subclinical lesions [20]. However, this trend likely reflects a combination of factors, including heightened public awareness, increased health screening coverage, and potential changes in environmental or lifestyle exposures. Therefore, caution should be exercised when interpreting these increases solely as overdiagnosis, and efforts should focus on refining diagnostic thresholds and promoting risk-based screening strategies to ensure appropriate management. Furthermore, breast cancer, cervical cancer, and TBL cancer also ranked among the leading cancers in terms of new incidence and mortality rates in female. The widespread use and diffusion of mammography globally from 2005–2015, along with the rapid development of emerging technologies such as ultrasound, magnetic resonance imaging, and genetic testing, has led to earlier screening ages and higher detection rates of early-onset breast cancer [2,21]. This has contributed in part to the increased incidence of breast cancer, but has facilitated timely interventions and may improve patient prognosis, survival and quality of life [22–25]. Oestrogen plays a key role in the development of breast cancer, and our study also identified diet high in red meat as the most important risk factor for early-onset breast cancer. Adipose tissue was one of the most important sources of oestrogen in the body, and red meat contains a certain amount of animal fat. When too much red meat is consumed, it may lead to the accumulation of fat in the body, which makes the oestrogen level in the body increase and leads to the development of breast cancer. Moreover, despite a decline in the incidence of cervical cancer among women following the introduction of the human papillomavirus vaccine [26], our analysis revealed that cervical cancer ranked third in terms of incidence and second in terms of mortality among early-onset cancers. The increase in smoking rates and the rise in unsafe sex were potential contributing factors. In particular, unsafe sex accounted for the entirety of the DALYs associated with cervical cancer. Similarly, TBL cancer also exhibited high incidence and mortality rates among both male and female in China, particularly in male. We found that the ASMR of early-onset TBL cancer in male was 3.3 times higher than that in female, which was mainly caused by the higher number of smokers in male. This finding was consistent with previous studies, which indicated that the high incidence of TBL cancer in young women was primarily due to second-hand smoke and indoor air pollution [27,28]. In addition to smoking and ambient particulate matter pollution, we also found that a diet low in fruits and HFPG contributed significantly to the DALYs associated with TBL cancer. It is consistent with the previous research results [29,30]. Thus, there was a need to implement a series of planned programmes of measures, including the adoption of mandatory tobacco control policies and the promotion of a balanced diet and glycaemic control, to further reduce the burden of early-onset TBL cancer.

In the trend analysis of early-onset cancers in China from 1990–2021 based on GBD 2021 data, we also observed that the incidence, mortality, and DALYs of some early-onset cancers had experienced significant increases over the past 30 years. It is equally urgent for China to strengthen its focus on these cancers with substantially rising burdens. For female, Neuroblastoma and other peripheral nerve cell tumours, non-melanoma skin cancer, malignant skin melanoma, and multiple myeloma were observed the greatest increase in ASIR, suggesting that the risk of these cancers may be increasing in female and that specific research and preventive strategies may need to be developed for female. Neuroblastoma and other peripheral nerve cell tumours, multiple myeloma, non-melanoma skin cancer, mesothelioma, and malignant neoplasm of bone and articular cartilage have shown the greatest increase in ASMR and ASDR in female, which poses a greater threat to female's health, and research on early diagnosis and treatment of these cancers in females should be strengthened. In terms of male, neuroblastoma and other peripheral nerve cell tumours, breast cancer, thyroid cancer, and multiple myeloma had the highest increase in ASIR, and neuroblastoma and other peripheral nerve cell tumours, multiple myeloma, breast cancer, and kidney cancer had the highest increase in ASMR and ASDR, which indicates that the burden of disease for male in these cancers is changing more dramatically, and that there is a need for in-depth study of the causes and formulation of appropriate prevention and control measures. This suggests that the disease burden of these cancers has changed significantly in male, and in-depth study of the causes is needed to develop appropriate prevention and control measures. It is noteworthy that the incidence of some early-onset cancers in China has increased while mortality has decreased. For example, the incidence of thyroid and breast cancers in female has risen, but mortality has declined. The incidence of thyroid and breast cancers in male has increased even more significantly, but mortality has also risen. The incidence of prostate cancer has increased, but mortality has decreased. This phenomenon may be attributed to the advancements in screening and diagnostic technologies in China, such as thyroid ultrasound [20], mammography [21], and prostate-specific antigen screening [31]. Additionally, advancements in targeted therapies, immunotherapy, and precision medicine have significantly increased survival rates for these cancers.

### Strength, limitations, and future research directions

In this study, we were the first to combine the latest cancer data from GBD 2021 and GLOBOCAN 2022 to assess the burden of early-onset cancers and their associated risk factors in China, offering several significant advantages. We emphasise that our analysis represents the best available estimates under current constraints. Benefiting from methodological improvements and the inclusion of more population-based cancer registry data, the current iterations of GBD 2021 and GLOBOCAN 2022 provided more accurate and reliable statistics than previous data. Specifically, GLOBOCAN focused on detailed cancer burden data, while GBD provided comprehensive time trends and multidimensional health data. This complementarity enabled a more thorough analysis, offering valuable estimates for policymakers and researchers, which could strengthen cancer prevention and control efforts in China. The findings from our study on early-onset cancers in China may be relevant to other countries undergoing similar epidemiologic transitions, particularly those with rapid urbanisation and changes in lifestyle factors. As countries experience shifts in risk factors such as smoking, high BMI, and aging populations, they may see similar patterns in cancer incidence, mortality, and DALYs. Additionally, improvements in cancer detection and health care access, as seen in China, could be mirrored in other nations, potentially leading to early diagnosis and improved survival rates. However, the specific burden and risk factor profiles will vary based on each country's unique demographic, socio-economic, and health care contexts, and tailored strategies will be needed to address these factors effectively. Cross-national collaborations, such as the WHO’s Global NCD Action Plan, could harmonise surveillance and policy frameworks to address these shared challenges. However, the limitations of this study must also be acknowledged. Thus, the limitations of each database must be considered when interpreting the results. Second, the COVID-19 pandemic had a significant impact on cancer diagnosis and treatment, but the GLOBOCAN 2022 estimates did not account for the effects of the pandemic, as these estimates were primarily based on pre-2020 data. The COVID-19 pandemic disrupted the continuity of cancer screening and treatment, potentially leading to delays in cancer diagnosis and care [32,33]. These factors may have led to the underestimation of cancer incidence and mortality in our study compared to the true cancer epidemiological data. Third, the mortality-to-incidence ratios assume stable survival rates over time, potentially underestimating mortality improvements from advancements in targeted therapies or deteriorations from diagnostic delays. Finally, China is a vast and diverse country with significant regional disparities in health care access, environmental exposures, and lifestyle factors. In the future, more granular analyses by region or province could provide additional insights into the distribution of cancer burden and help tailor prevention strategies for specific areas.

## CONCLUSIONS

This study, integrating GLOBOCAN 2022 and GBD 2021 data, provides the first comprehensive assessment of early-onset cancers in China. The findings reveal distinct sex and cancer-specific patterns, with rising incidence and shifting risk factors over the past three decades. Thyroid, breast, cervical, and TBL cancers remain the major contributors to the disease burden. While advances in detection and therapy have improved survival for some cancers, others – such as neuroblastoma, multiple myeloma, and kidney cancer – are increasing rapidly. Targeted prevention, early detection, and regionally tailored control strategies are urgently needed to reduce the growing burden of early-onset cancers in China.

## Additional material


Online Supplementary Document
