# Supplementary material for: Status and trends of early-onset cancers and their risk factors in China: population-based study
Source: J Glob Health. 2026 Jan 12;16:04005. doi: 10.7189/jogh.16.04005 (PMC12793929; doi:10.7189/jogh.16.04005)
Supplement: Online Supplementary Document [file jogh-16-04005-s001.pdf]

Supplement to: Yun Z, Yang Q, Han X, Wang C, Wang M, Wang Y, Zhang Y, Wang N, Zhang L, Kong F. Status and trends of early-onset cancers and their risk factors in China: population-based study. J Glob Health. 2026;16:04005.

Catalogue

Table S1: List of International Classification of Diseases (ICD) codes mapped to non-fatal causes and injuries in the GBD 2021 and GLOBOCAN 2022. 1
Table S2: Estimated absolute number, proportion, and ASRs of incidence and mortality by cancer type and sex, among early-onset cancers aged 15 to 19 years worldwide in 2022. 3
Table S3: Estimated absolute number, proportion, and ASRs of incidence and mortality by cancer type and sex, among early-onset cancers aged 20 to 24 years worldwide in 2022. 5
Table S4: Estimated absolute number, proportion, and ASRs of incidence and mortality by cancer type and sex, among early-onset cancers aged 25 to 29 years worldwide in 2022. 7
Table S5: Estimated absolute number, proportion, and ASRs of incidence and mortality by cancer type and sex, among early-onset cancers aged 30 to 34 years worldwide in 2022. 9
Table S6: Estimated absolute number, proportion, and ASRs of incidence and mortality by cancer type and sex, among early-onset cancers aged 35 to 39 years worldwide in 2022. 11
Table S7: Estimated absolute number, proportion, and ASRs of incidence and mortality by cancer type and sex, among early-onset cancers aged 40 to 44 years worldwide in 2022. 13
Table S8: Estimated absolute number, proportion, and ASRs of incidence and mortality by cancer type and sex, among early-onset cancers aged 45 to 49 years worldwide in 2022. 15
Table S9: The incidence, mortality, and DALYs number for 32 early-onset cancers in 1990 and 2021 in China, with TPC from 1990 to 2021. 17
Table S10: The incidence, mortality, and DALYs number for 30 female early-onset cancers in 1990 and 2021 in China, with TPC from 1990 to 2021. 21
Table S11: The incidence, mortality, and DALYs number for 29 male early-onset cancers in 1990 and 2021 in China, with TPC from 1990 to 2021. 25
Table S12: The ASR of incidence, mortality, and DALYs for 30 female early-onset cancers in 1990 and 2021 in China, with AAPC from 1990 to 2021. 28
Table S13: The ASR of incidence, mortality, and DALYs for 29 male early-onset cancers in 1990 and 2021 in China, with AAPC from 1990 to 2021. 31
Table S14: Percentage contribution of environmental and occupational risks to early-onset cancers DALYs in China by sex, 2021. 33
Table S15: Percentage contribution of behavioral and metabolic risks to early-onset cancers DALYs in China by sex, 2021. 36
Table S16: Age-standardized DALYs rate of average annual percent change in early-onset cancers attributable to risk factors from 1990 to 2021 by sex. 42
Figure S1: Top five cancers with the highest increase in ASIR, ASMR and ASDR by sex in China from 1990–2021. 52
Figure S2: Trends in incidence, mortality and DALYs rates for early-onset cancers in China by age group from 1990 to 2021 by sex. 52

Table S1: List of International Classification of Diseases (ICD) codes mapped to non-fatal causes and injuries in the GBD 2021 and GLOBOCAN 2022.

GBD 2021		GLOBOCAN 2022	
**Cancer type**	**ICD10**	**Cancer type**	**ICD10**
Bladder cancer	C67-C67.9	Bladder	C67
Brain and central nervous system cancer	C70-C70.1, C70.9-C72.9	Brain, central nervous system	C70-C72
Breast cancer	C50-C50.629, C50.8-C50.929	Breast	C50
Cervical cancer	C53-C53.9	Cervix uteri	C53
Colon and rectum cancer	C18-C19.0, C20, C21-C21.8	Colorectum	C18–C21
Esophageal cancer	C15-C15.9	Corpus uteri	C54
Eye cancer	C69-C69.92	Gallbladder	C23
Gallbladder and biliary tract cancer	C23, C24-C24.9	Hodgkin lymphoma	C81
Hodgkin lymphoma	C81-C81.49, C81.7-C81.79, C81.9-C81.99	Hypopharynx	C32
Kidney cancer	C64-C64.2, C64.9-C65.9	Kaposi sarcoma	C46
Larynx cancer	C32-C32.9	Kidney	C64-C65
Leukemia	C91-C93.7, C93.9-C95.2, C95.7-C95.92	Larynx	C32
Lip and oral cavity cancer	C00-C07, C08-C08.9	Leukaemia	C91–C95
Liver cancer	C22-C22.4, C22.7-C22.8	Lip, oral cavity	C00–C06
Malignant neoplasm of bone and articular cartilage	C40-C40.92, C41.0-C41.4, C41.8-C41.9	Liver and intrahepatic bile ducts	C22
Malignant skin melanoma	C43-C43.9	Melanoma of skin	C43
Mesothelioma	C45-C45.2, C45.7.C45.9	Mesothelioma	C45
Multiple myeloma	C88-C90.32	Multiple myeloma	C88, C90
Nasopharynx cancer	C11-C11.9	Nasopharynx	C11
Neuroblastoma and other peripheral nervous cell tumors	C47-C47.9	Non-Hodgkin lymphoma	C82-C86, C96
Non-Hodgkin lymphoma	C82-C85.29, C85.7-C86.6, C96-C96.9	Non-Hodgkin lymphoma	C44
Non-melanoma skin cancer	C44.01-C44.99	Oesophagus	C15
Other pharynx cancer	C09-C10.9, C12-C13.9	Oropharynx	C09–C10
Ovarian cancer	C56-C56.2, C56.9	Ovary	C56
Pancreatic cancer	C25-C25.9	Pancreas	C25
Prostate cancer	C61-C61.9	Penis	C60
Soft tissue and other extraosseous sarcomas	C49-C49.9	Prostate	C61
Stomach cancer	C16-C16.9	Salivary glands	C07–C08

Testicular cancer	C62-C62.92	Stomach	C16
Thyroid cancer	C73	Testis	C62
Tracheal, bronchus, and lung cancer	C33, C34-C34.92	Thyroid	C73
Uterine cancer	C54-C54.3, C54.8-C54.9	Trachea, bronchus and lung	C33-C34
		Vagina	C52
		Vulva	C51

Table S2: Estimated absolute number, proportion, and ASRs of incidence and mortality by cancer type and sex, among early-onset cancers aged 15 to 19 years worldwide in 2022.

Cancer type	Estimated new cancer cases and ASR per 100,000 people per year									Estimated cancer-related deaths and ASR per 100,000 people per year								
	Both sex			Female			Male			Both sex			Female			Male		
	Cases	(%)	ASR	Cases	(%)	ASR	Cases	(%)	ASR	Cases	(%)	ASR	Cases	(%)	ASR	Cases	(%)	ASR
All cancers	7836	100%	12.52	4098	100%	13.41	3738	100%	11.76	2203	100%	3.76	892	100%	3.16	1311	100%	4.28
Bladder	31	0.40%	0.04	6	0.15%	0.02	25	0.67%	0.06	6	0.27%	0.01	1	0.11%	0	5	0.38%	0.01
Brain, central nervous system	938	11.97%	1.21	392	9.57%	1.1	546	14.61%	1.32	463	21.02%	0.6	167	18.72%	0.47	296	22.58%	0.71
Breast	120	1.53%	0.34	120	2.93%	0.34	0	0.00%	0	12	0.54%	0.03	12	1.35%	0.03	0	0.00%	0
Cervix uteri	25	0.32%	0.07	25	0.61%	0.07	0	0.00%	0	0	0.00%	0	0	0.00%	0	0	0.00%	0
Colorectum	239	3.05%	0.31	78	1.90%	0.22	161	4.31%	0.39	87	3.95%	0.11	33	3.70%	0.09	54	4.12%	0.13
Corpus uteri	12	0.15%	0.03	12	0.29%	0.03	0	0.00%	0	1	0.05%	0	1	0.11%	0	0	0.00%	0
Gallbladder	5	0.06%	0.01	4	0.10%	0.01	1	0.03%	0	0	0.00%	0	0	0.00%	0	0	0.00%	0
Hodgkin lymphoma	151	1.93%	0.2	44	1.07%	0.12	107	2.86%	0.26	15	0.68%	0.02	7	0.78%	0.02	8	0.61%	0.02
Hypopharynx	0	0.00%	0	0	0.00%	0	0	0.00%	0	2	0.09%	0	0	0.00%	0	2	0.15%	0
Kaposi sarcoma	9	0.11%	0.01	9	0.22%	0.03	0	0.00%	0	19	0.86%	0.02	19	2.13%	0.05	0	0.00%	0
Kidney	89	1.14%	0.12	33	0.81%	0.09	56	1.50%	0.14	27	1.23%	0.03	4	0.45%	0.01	23	1.75%	0.06
Larynx	1	0.01%	0	0	0.00%	0	1	0.03%	0	5	0.23%	0.01	0	0.00%	0	5	0.38%	0.01
Leukaemia	1747	22.29%	2.26	616	15.03%	1.72	1131	30.26%	2.73	877	39.81%	1.14	354	39.69%	0.99	523	39.89%	1.26
Lip, oral cavity	42	0.54%	0.05	19	0.46%	0.05	23	0.62%	0.06	4	0.18%	0.01	4	0.45%	0.01	0	0.00%	0
Liver and intrahepatic bile ducts	188	2.40%	0.24	63	1.54%	0.18	125	3.34%	0.3	152	6.90%	0.2	40	4.48%	0.11	112	8.54%	0.27
Melanoma of skin	38	0.48%	0.05	25	0.61%	0.07	13	0.35%	0.03	6	0.27%	0.01	0	0.00%	0	6	0.46%	0.01
Mesothelioma	5	0.06%	0.01	4	0.10%	0.01	1	0.03%	0	0	0.00%	0	0	0.00%	0	0	0.00%	0
Multiple myeloma	42	0.54%	0.05	9	0.22%	0.03	33	0.88%	0.08	53	2.41%	0.07	28	3.14%	0.08	25	1.91%	0.06
Nasopharynx	192	2.45%	0.25	55	1.34%	0.15	137	3.67%	0.33	37	1.68%	0.05	3	0.34%	0.01	34	2.59%	0.08
Non-Hodgkin lymphoma	670	8.55%	0.87	190	4.64%	0.53	480	12.84%	1.16	210	9.53%	0.27	74	8.30%	0.21	136	10.37%	0.33
Non-melanoma skin cancer	154	1.97%	0.2	85	2.07%	0.24	69	1.85%	0.17	6	0.27%	0.01	3	0.34%	0.01	3	0.23%	0.01
Oesophagus	15	0.19%	0.02	5	0.12%	0.01	10	0.27%	0.02	4	0.18%	0.01	0	0.00%	0	4	0.31%	0.01
Oropharynx	21	0.27%	0.03	12	0.29%	0.03	9	0.24%	0.02	7	0.32%	0.01	7	0.78%	0.02	0	0.00%	0
Ovary	460	5.87%	1.29	460	11.22%	1.29	0	0.00%	0	65	2.95%	0.18	65	7.29%	0.18	0	0.00%	0
Pancreas	38	0.48%	0.05	20	0.49%	0.06	18	0.48%	0.04	13	0.59%	0.02	12	1.35%	0.03	1	0.08%	0

Penis	7	0.09%	0.02	0	0.00%	0	7	0.19%	0.02	5	0.23%	0.01	0	0.00%	0	5	0.38%	0.01
Prostate	8	0.10%	0.02	0	0.00%	0	8	0.21%	0.02	18	0.82%	0.04	0	0.00%	0	18	1.37%	0.04
Salivary glands	114	1.45%	0.15	69	1.68%	0.19	45	1.20%	0.11	2	0.09%	0	2	0.22%	0.01	0	0.00%	0
Stomach	113	1.44%	0.15	47	1.15%	0.13	66	1.77%	0.16	41	1.86%	0.05	29	3.25%	0.08	12	0.92%	0.03
Testis	75	0.96%	0.18	0	0.00%	0	75	2.01%	0.18	6	0.27%	0.01	0	0.00%	0	6	0.46%	0.01
Thyroid	2093	26.71%	2.71	1590	38.80%	4.44	503	13.46%	1.21	9	0.41%	0.01	7	0.78%	0.02	2	0.15%	0
Trachea, bronchus and lung	176	2.25%	0.23	88	2.15%	0.25	88	2.35%	0.21	51	2.32%	0.07	20	2.24%	0.06	31	2.36%	0.07
Vagina	9	0.11%	0.03	9	0.22%	0.03	0	0.00%	0	0	0.00%	0	0	0.00%	0	0	0.00%	0
Vulva	9	0.11%	0.03	9	0.22%	0.03	0	0.00%	0	0	0.00%	0	0	0.00%	0	0	0.00%	0

Table S3: Estimated absolute number, proportion, and ASRs of incidence and mortality by cancer type and sex, among early-onset cancers aged 20 to 24 years worldwide in 2022.

Cancer type	Estimated new cancer cases and ASR per 100,000 people per year									Estimated cancer-related deaths and ASR per 100,000 people per year								
	Both sex			Female			Male			Both sex			Female			Male		
	Cases	(%)	ASR	Cases	(%)	ASR	Cases	(%)	ASR	Cases	(%)	ASR	Cases	(%)	ASR	Cases	(%)	ASR
All cancers	15447	100%	23.37	9524	100%	30.06	5923	100%	17.50	2355	100%	4.01	915	100%	3.26	1440	100%	4.67
Bladder	70	0.45%	0.10	25	0.26%	0.07	45	0.76%	0.12	6	0.25%	0.01	1	0.11%	0.00	5	0.35%	0.01
Brain, central nervous system	817	5.29%	1.13	405	4.25%	1.20	412	6.96%	1.07	325	13.80%	0.45	114	12.46%	0.34	211	14.65%	0.55
Breast	531	3.44%	1.57	531	5.58%	1.57	0	0	0.00	22	0.93%	0.07	22	2.40%	0.07	0	0	0.00
Cervix uteri	189	1.22%	0.56	189	1.98%	0.56	0	0	0.00	46	1.95%	0.14	46	5.03%	0.14	0	0	0.00
Colorectum	506	3.28%	0.70	248	2.60%	0.73	258	4.36%	0.67	185	7.86%	0.26	60	6.56%	0.18	125	8.68%	0.32
Corpus uteri	83	0.54%	0.25	83	0.87%	0.25	0	0	0.00	7	0.30%	0.02	7	0.77%	0.02	0	0	0.00
Gallbladder	8	0.05%	0.01	7	0.07%	0.02	1	0.02%	0.00	1	0.04%	0.00	0	0	0.00	1	0.07%	0.00
Hodgkin lymphoma	187	1.21%	0.26	94	0.99%	0.28	93	1.57%	0.24	18	0.76%	0.02	8	0.87%	0.02	10	0.69%	0.03
Hypopharynx	8	0.05%	0.01	4	0.04%	0.01	4	0.07%	0.01	1	0.04%	0.00	0	0	0.00	1	0.07%	0.00
Kaposi sarcoma	9	0.06%	0.01	0	0	0.00	9	0.15%	0.02	9	0.38%	0.01	5	0.55%	0.01	4	0.28%	0.01
Kidney	166	1.07%	0.23	64	0.67%	0.19	102	1.72%	0.27	31	1.32%	0.04	19	2.08%	0.06	12	0.83%	0.03
Larynx	15	0.10%	0.02	8	0.08%	0.02	7	0.12%	0.02	9	0.38%	0.01	4	0.44%	0.01	5	0.35%	0.01
Leukaemia	1333	8.63%	1.85	540	5.67%	1.60	793	13.39%	2.06	654	27.77%	0.91	226	24.70%	0.67	428	29.72%	1.11
Lip, oral cavity	84	0.54%	0.12	43	0.45%	0.13	41	0.69%	0.11	15	0.64%	0.02	11	1.20%	0.03	4	0.28%	0.01
Liver and intrahepatic bile ducts	389	2.52%	0.54	117	1.23%	0.35	272	4.59%	0.71	262	11.13%	0.36	62	6.78%	0.18	200	13.89%	0.52
Melanoma of skin	36	0.23%	0.05	9	0.09%	0.03	27	0.46%	0.07	13	0.55%	0.02	1	0.11%	0.00	12	0.83%	0.03
Mesothelioma	12	0.08%	0.02	4	0.04%	0.01	8	0.14%	0.02	4	0.17%	0.01	1	0.11%	0.00	3	0.21%	0.01
Multiple myeloma	23	0.15%	0.03	12	0.13%	0.04	11	0.19%	0.03	26	1.10%	0.04	19	2.08%	0.06	7	0.49%	0.02
Nasopharynx	251	1.62%	0.35	104	1.09%	0.31	147	2.48%	0.38	32	1.36%	0.04	12	1.31%	0.04	20	1.39%	0.05
Non-Hodgkin lymphoma	594	3.85%	0.82	231	2.43%	0.68	363	6.13%	0.94	188	7.98%	0.26	48	5.25%	0.14	140	9.72%	0.36
Non-melanoma skin cancer	136	0.88%	0.19	75	0.79%	0.22	61	1.03%	0.16	14	0.59%	0.02	3	0.33%	0.01	11	0.76%	0.03
Oesophagus	33	0.21%	0.05	17	0.18%	0.05	16	0.27%	0.04	24	1.02%	0.03	12	1.31%	0.04	12	0.83%	0.03
Oropharynx	19	0.12%	0.03	6	0.06%	0.02	13	0.22%	0.03	9	0.38%	0.01	0	0	0.00	9	0.63%	0.02
Ovary	654	4.23%	1.94	654	6.87%	1.94	0	0	0.00	56	2.38%	0.17	56	6.12%	0.17	0	0	0.00
Pancreas	89	0.58%	0.12	65	0.68%	0.19	24	0.41%	0.06	22	0.93%	0.03	6	0.66%	0.02	16	1.11%	0.04
Penis	2	0.01%	0.01	0	0	0.00	2	0.03%	0.01	0	0.00%	0.00	0	0	0.00	0	0	0.00

Prostate	15	0.10%	0.04	0	0	0.00	15	0.25%	0.04	5	0.21%	0.01	0	0	0.00	5	0.35%	0.01
Salivary glands	117	0.76%	0.16	53	0.56%	0.16	64	1.08%	0.17	9	0.38%	0.01	5	0.55%	0.01	4	0.28%	0.01
Stomach	239	1.55%	0.33	129	1.35%	0.38	110	1.86%	0.29	123	5.22%	0.17	68	7.43%	0.20	55	3.82%	0.14
Testis	147	0.95%	0.38	0	0	0.00	147	2.48%	0.38	17	0.72%	0.04	0	0	0.00	17	1.18%	0.04
Thyroid	8153	52.78%	11.29	5558	58.36%	16.46	2595	43.81%	6.75	37	1.57%	0.05	27	2.95%	0.08	10	0.69%	0.03
Trachea, bronchus and lung	522	3.38%	0.72	239	2.51%	0.71	283	4.78%	0.74	181	7.69%	0.25	68	7.43%	0.20	113	7.85%	0.29
Vagina	1	0.01%	0.00	1	0.01%	0.00	0	0	0.00	0	0	0.00	0	0	0.00	0	0	0
Vulva	9	0.06%	0.03	9	0.09%	0.03	0	0	0.00	4	0.17%	0.01	4	0.44%	0.01	0	0	0

Table S4: Estimated absolute number, proportion, and ASRs of incidence and mortality by cancer type and sex, among early-onset cancers aged 25 to 29 years worldwide in 2022.

Cancer type	Estimated new cancer cases and ASR per 100,000 people per year									Estimated cancer-related deaths and ASR per 100,000 people per year								
	Both sex			Female			Male			Both sex			Female			Male		
	Cases	(%)	ASR	Cases	(%)	ASR	Cases	(%)	ASR	Cases	(%)	ASR	Cases	(%)	ASR	Cases	(%)	ASR
All cancers	45714	100%	57.49	28970	100%	75.93	16744	100%	40.89	5371	100%	7.31	2269	100%	6.42	3102	100%	8.11
Bladder	258	0.56%	0.31	81	0.28%	0.20	177	1.06%	0.40	26	0.48%	0.03	6	0.26%	0.02	20	0.64%	0.05
Brain, central nervous system	1409	3.08%	1.69	622	2.15%	1.57	787	4.70%	1.79	634	11.80%	0.76	205	9.03%	0.52	429	13.83%	0.98
Breast	2675	5.85%	6.76	2675	9.23%	6.76	0	0	0.00	213	3.97%	0.54	213	9.39%	0.54	0	0	0.00
Cervix uteri	1448	3.17%	3.66	1448	5.00%	3.66	0	0	0.00	224	4.17%	0.57	224	9.87%	0.57	0	0	0.00
Colorectum	1554	3.40%	1.86	630	2.17%	1.59	924	5.52%	2.10	466	8.68%	0.56	173	7.62%	0.44	293	9.45%	0.67
Corpus uteri	419	0.92%	1.06	419	1.45%	1.06	0	0	0.00	37	0.69%	0.09	37	1.63%	0.09	0	0	0.00
Gallbladder	22	0.05%	0.03	14	0.05%	0.04	8	0.05%	0.02	10	0.19%	0.01	3	0.13%	0.01	7	0.23%	0.02
Hodgkin lymphoma	254	0.56%	0.30	124	0.43%	0.31	130	0.78%	0.30	32	0.60%	0.04	6	0.26%	0.02	26	0.84%	0.06
Hypopharynx	9	0.02%	0.01	1	0.00%	0.00	8	0.05%	0.02	8	0.15%	0.01	4	0.18%	0.01	4	0.13%	0.01
Kaposi sarcoma	21	0.05%	0.03	1	0.00%	0.00	20	0.12%	0.05	9	0.17%	0.01	3	0.13%	0.01	6	0.19%	0.01
Kidney	410	0.90%	0.49	137	0.47%	0.35	273	1.63%	0.62	67	1.25%	0.08	22	0.97%	0.06	45	1.45%	0.10
Larynx	17	0.04%	0.02	1	0.00%	0.00	16	0.10%	0.04	10	0.19%	0.01	6	0.26%	0.02	4	0.13%	0.01
Leukaemia	2051	4.49%	2.46	890	3.07%	2.25	1161	6.93%	2.64	948	17.65%	1.13	369	16.26%	0.93	579	18.67%	1.32
Lip, oral cavity	212	0.46%	0.25	93	0.32%	0.24	119	0.71%	0.27	32	0.60%	0.04	22	0.97%	0.06	10	0.32%	0.02
Liver and intrahepatic bile ducts	1360	2.98%	1.63	315	1.09%	0.80	1045	6.24%	2.38	865	16.11%	1.04	170	7.49%	0.43	695	22.40%	1.58
Melanoma of skin	81	0.18%	0.10	44	0.15%	0.11	37	0.22%	0.08	39	0.73%	0.05	24	1.06%	0.06	15	0.48%	0.03
Mesothelioma	13	0.03%	0.02	10	0.03%	0.03	3	0.02%	0.01	1	0.02%	0.00	1	0.04%	0.00	0	0	0.00
Multiple myeloma	89	0.19%	0.11	42	0.14%	0.11	47	0.28%	0.11	49	0.91%	0.06	14	0.62%	0.04	35	1.13%	0.08
Nasopharynx	713	1.56%	0.85	218	0.75%	0.55	495	2.96%	1.13	139	2.59%	0.17	31	1.37%	0.08	108	3.48%	0.25
Non-Hodgkin lymphoma	1011	2.21%	1.21	429	1.48%	1.08	582	3.48%	1.32	240	4.47%	0.29	69	3.04%	0.17	171	5.51%	0.39
Non-melanoma skin cancer	288	0.63%	0.34	121	0.42%	0.31	167	1.00%	0.38	35	0.65%	0.04	21	0.93%	0.05	14	0.45%	0.03
Oesophagus	84	0.18%	0.10	46	0.16%	0.12	38	0.23%	0.09	27	0.50%	0.03	17	0.75%	0.04	10	0.32%	0.02
Oropharynx	27	0.06%	0.03	6	0.02%	0.02	21	0.13%	0.05	6	0.11%	0.01	4	0.18%	0.01	2	0.06%	0.00
Ovary	1107	2.42%	2.80	1107	3.82%	2.80	0	0	0.00	161	3.00%	0.41	161	7.10%	0.41	0	0	0.00
Pancreas	166	0.36%	0.20	95	0.33%	0.24	71	0.42%	0.16	88	1.64%	0.11	38	1.67%	0.10	50	1.61%	0.11
Penis	37	0.08%	0.08	0	0	0.00	37	0.22%	0.08	1	0.02%	0.00	0	0	0.00	1	0.03%	0.00

Prostate	22	0.05%	0.05	0	0	0.00	22	0.13%	0.05	10	0.19%	0.02	0	0	0.00	10	0.32%	0.02
Salivary glands	254	0.56%	0.30	137	0.47%	0.35	117	0.70%	0.27	15	0.28%	0.02	4	0.18%	0.01	11	0.35%	0.03
Stomach	867	1.90%	1.04	518	1.79%	1.31	349	2.08%	0.79	363	6.76%	0.43	173	7.62%	0.44	190	6.13%	0.43
Testis	308	0.67%	0.70	0	0	0.00	308	1.84%	0.70	30	0.56%	0.07	0	0	0.00	30	0.97%	0.07
Thyroid	26790	58.60%	32.07	17799	61.44%	44.99	8991	53.70%	20.45	122	2.27%	0.15	82	3.61%	0.21	40	1.29%	0.09
Trachea, bronchus and lung	1693	3.70%	2.03	902	3.11%	2.28	791	4.72%	1.80	455	8.47%	0.54	158	6.96%	0.40	297	9.57%	0.68
Vagina	18	0.04%	0.05	18	0.06%	0.05	0	0	0.00	1	0.02%	0.00	1	0.04%	0.00	0	0	0.00
Vulva	27	0.06%	0.07	27	0.09%	0.07	0	0	0.00	8	0.15%	0.02	8	0.35%	0.02	0	0	0.00

Table S5: Estimated absolute number, proportion, and ASRs of incidence and mortality by cancer type and sex, among early-onset cancers aged 30 to 34 years worldwide in 2022.

Cancer type	Estimated new cancer cases and ASR per 100,000 people per year									Estimated cancer-related deaths and ASR per 100,000 people per year								
	Both sex			Female			Male			Both sex			Female			Male		
	Cases	(%)	ASR	Cases	(%)	ASR	Cases	(%)	ASR	Cases	(%)	ASR	Cases	(%)	ASR	Cases	(%)	ASR
All cancers	103260	100%	93.28	67786	100%	125.76	35474	100%	63.04	14009	100%	13.20	6276	100%	12.19	7733	100%	14.15
Bladder	629	0.61%	0.55	145	0.21%	0.26	484	1.36%	0.81	21	0.15%	0.02	9	0.14%	0.02	12	0.16%	0.02
Brain, central nervous system	2655	2.57%	2.31	1118	1.65%	2.01	1537	4.33%	2.58	1237	8.83%	1.07	438	6.98%	0.79	799	10.33%	1.34
Breast	10405	10.08%	18.75	10405	15.35%	18.75	0	0	0.00	1013	7.23%	1.83	1013	16.14%	1.83	0	0	0.00
Cervix uteri	5041	4.88%	9.08	5041	7.44%	9.08	0	0	0.00	774	5.53%	1.39	774	12.33%	1.39	0	0	0.00
Colorectum	4125	3.99%	3.58	1875	2.77%	3.38	2250	6.34%	3.78	1264	9.02%	1.10	603	9.61%	1.09	661	8.55%	1.11
Corpus uteri	1079	1.04%	1.94	1079	1.59%	1.94	0	0	0.00	135	0.96%	0.24	135	2.15%	0.24	0	0	0.00
Gallbladder	70	0.07%	0.06	27	0.04%	0.05	43	0.12%	0.07	60	0.43%	0.05	32	0.51%	0.06	28	0.36%	0.05
Hodgkin lymphoma	320	0.31%	0.28	150	0.22%	0.27	170	0.48%	0.29	33	0.24%	0.03	19	0.30%	0.03	14	0.18%	0.02
Hypopharynx	16	0.02%	0.01	0	0.00%	0.00	16	0.05%	0.03	6	0.04%	0.01	0	0.00%	0.00	6	0.08%	0.01
Kaposi sarcoma	7	0.01%	0.01	0	0.00%	0.00	7	0.02%	0.01	11	0.08%	0.01	4	0.06%	0.01	7	0.09%	0.01
Kidney	1308	1.27%	1.14	498	0.73%	0.90	810	2.28%	1.36	190	1.36%	0.17	80	1.27%	0.14	110	1.42%	0.18
Larynx	39	0.04%	0.03	20	0.03%	0.04	19	0.05%	0.03	21	0.15%	0.02	5	0.08%	0.01	16	0.21%	0.03
Leukaemia	3008	2.91%	2.61	1248	1.84%	2.25	1760	4.96%	2.95	1274	9.09%	1.11	492	7.84%	0.89	782	10.11%	1.31
Lip, oral cavity	607	0.59%	0.53	260	0.38%	0.47	347	0.98%	0.58	138	0.99%	0.12	40	0.64%	0.07	98	1.27%	0.16
Liver and intrahepatic bile ducts	4210	4.08%	3.66	735	1.08%	1.32	3475	9.80%	5.83	2979	21.26%	2.59	471	7.50%	0.85	2508	32.43%	4.21
Melanoma of skin	113	0.11%	0.10	72	0.11%	0.13	41	0.12%	0.07	46	0.33%	0.04	21	0.33%	0.04	25	0.32%	0.04
Mesothelioma	27	0.03%	0.02	10	0.01%	0.02	17	0.05%	0.03	19	0.14%	0.02	0	0.00%	0.00	19	0.25%	0.03
Multiple myeloma	153	0.15%	0.13	63	0.09%	0.11	90	0.25%	0.15	67	0.48%	0.06	22	0.35%	0.04	45	0.58%	0.08
Nasopharynx	1974	1.91%	1.72	649	0.96%	1.17	1325	3.74%	2.22	378	2.70%	0.33	108	1.72%	0.19	270	3.49%	0.45
Non-Hodgkin lymphoma	2082	2.02%	1.81	875	1.29%	1.58	1207	3.40%	2.03	535	3.82%	0.46	180	2.87%	0.32	355	4.59%	0.60
Non-melanoma skin cancer	605	0.59%	0.53	291	0.43%	0.52	314	0.89%	0.53	59	0.42%	0.05	13	0.21%	0.02	46	0.59%	0.08
Oesophagus	147	0.14%	0.13	69	0.10%	0.12	78	0.22%	0.13	83	0.59%	0.07	22	0.35%	0.04	61	0.79%	0.10
Oropharynx	62	0.06%	0.05	17	0.03%	0.03	45	0.13%	0.08	8	0.06%	0.01	0	0.00%	0.00	8	0.10%	0.01
Ovary	1754	1.70%	3.16	1754	2.59%	3.16	0	0	0.00	312	2.23%	0.56	312	4.97%	0.56	0	0	0.00
Pancreas	455	0.44%	0.40	212	0.31%	0.38	243	0.69%	0.41	286	2.04%	0.25	125	1.99%	0.23	161	2.08%	0.27
Penis	70	0.07%	0.12	0	0	0.00	70	0.20%	0.12	17	0.12%	0.03	0	0	0.00	17	0.22%	0.03

Prostate	73	0.07%	0.12	0	0	0.00	73	0.21%	0.12	32	0.23%	0.05	0	0	0.00	32	0.41%	0.05
Salivary glands	404	0.39%	0.35	182	0.27%	0.33	222	0.63%	0.37	46	0.33%	0.04	19	0.30%	0.03	27	0.35%	0.05
Stomach	2258	2.19%	1.96	1318	1.94%	2.37	940	2.65%	1.58	1198	8.55%	1.04	630	10.04%	1.14	568	7.35%	0.95
Testis	549	0.53%	0.92	0	0	0.00	549	1.55%	0.92	53	0.38%	0.09	0	0	0.00	53	0.69%	0.09
Thyroid	53949	52.25%	46.87	36600	53.99%	65.95	17349	48.91%	29.11	177	1.26%	0.15	134	2.14%	0.24	43	0.56%	0.07
Trachea, bronchus and lung	4980	4.82%	4.33	2987	4.41%	5.38	1993	5.62%	3.34	1515	10.81%	1.32	553	8.81%	1.00	962	12.44%	1.61
Vagina	37	0.04%	0.07	37	0.05%	0.07	0	0	0.00	10	0.07%	0.02	10	0.16%	0.02	0	0	0.00
Vulva	49	0.05%	0.09	49	0.07%	0.09	0	0	0.00	12	0.09%	0.02	12	0.19%	0.02	0	0	0.00

Table S6: Estimated absolute number, proportion, and ASRs of incidence and mortality by cancer type and sex, among early-onset cancers aged 35 to 39 years worldwide in 2022.

Cancer type	Estimated new cancer cases and ASR per 100,000 people per year									Estimated cancer-related deaths and ASR per 100,000 people per year								
	Both sex			Female			Male			Both sex			Female			Male		
	Cases	(%)	ASR	Cases	(%)	ASR	Cases	(%)	ASR	Cases	(%)	ASR	Cases	(%)	ASR	Cases	(%)	ASR
All cancers	128896	100%	125.24	86334	100%	170.75	42562	100%	82.17	20579	100%	20.60	9020	100%	18.52	11559	100%	22.57
Bladder	790	0.61%	0.74	165	0.19%	0.32	625	1.47%	1.14	66	0.32%	0.06	12	0.13%	0.02	54	0.47%	0.10
Brain, central nervous system	2842	2.20%	2.66	1282	1.48%	2.47	1560	3.67%	2.84	1233	5.99%	1.15	437	4.84%	0.84	796	6.89%	1.45
Breast	18603	14.43%	35.81	18603	21.55%	35.81	0	0	0.00	1982	9.63%	3.82	1982	21.97%	3.82	0	0	0.00
Cervix uteri	8101	6.28%	15.59	8101	9.38%	15.59	0	0	0.00	1408	6.84%	2.71	1408	15.61%	2.71	0	0	0.00
Colorectum	6052	4.70%	5.66	2700	3.13%	5.20	3352	7.88%	6.11	1690	8.21%	1.58	650	7.21%	1.25	1040	9.00%	1.89
Corpus uteri	1931	1.50%	3.72	1931	2.24%	3.72	0	0	0.00	151	0.73%	0.29	151	1.67%	0.29	0	0	0.00
Gallbladder	144	0.11%	0.13	82	0.09%	0.16	62	0.15%	0.11	75	0.36%	0.07	39	0.43%	0.08	36	0.31%	0.07
Hodgkin lymphoma	248	0.19%	0.23	145	0.17%	0.28	103	0.24%	0.19	48	0.23%	0.04	18	0.20%	0.03	30	0.26%	0.05
Hypopharynx	42	0.03%	0.04	5	0.01%	0.01	37	0.09%	0.07	26	0.13%	0.02	1	0.01%	0.00	25	0.22%	0.05
Kaposi sarcoma	34	0.03%	0.03	13	0.02%	0.03	21	0.05%	0.04	1	0.00%	0.00	1	0.01%	0.00	0	0.00%	0.00
Kidney	1880	1.46%	1.76	562	0.65%	1.08	1318	3.10%	2.40	150	0.73%	0.14	35	0.39%	0.07	115	0.99%	0.21
Larynx	98	0.08%	0.09	17	0.02%	0.03	81	0.19%	0.15	52	0.25%	0.05	8	0.09%	0.02	44	0.38%	0.08
Leukaemia	2673	2.07%	2.50	1160	1.34%	2.23	1513	3.55%	2.76	1201	5.84%	1.12	483	5.35%	0.93	718	6.21%	1.31
Lip, oral cavity	691	0.54%	0.65	228	0.26%	0.44	463	1.09%	0.84	196	0.95%	0.18	44	0.49%	0.08	152	1.31%	0.28
Liver and intrahepatic bile ducts	6894	5.35%	6.45	1054	1.22%	2.03	5840	13.72%	10.64	5052	24.55%	4.73	691	7.66%	1.33	4361	37.73%	7.94
Melanoma of skin	156	0.12%	0.15	77	0.09%	0.15	79	0.19%	0.14	78	0.38%	0.07	29	0.32%	0.06	49	0.42%	0.09
Mesothelioma	27	0.02%	0.03	12	0.01%	0.02	15	0.04%	0.03	24	0.12%	0.02	12	0.13%	0.02	12	0.10%	0.02
Multiple myeloma	236	0.18%	0.22	98	0.11%	0.19	138	0.32%	0.25	129	0.63%	0.12	39	0.43%	0.08	90	0.78%	0.16
Nasopharynx	2719	2.11%	2.54	760	0.88%	1.46	1959	4.60%	3.57	665	3.23%	0.62	157	1.74%	0.30	508	4.39%	0.93
Non-Hodgkin lymphoma	2130	1.65%	1.99	1028	1.19%	1.98	1102	2.59%	2.01	578	2.81%	0.54	208	2.31%	0.40	370	3.20%	0.67
Non-melanoma skin cancer	737	0.57%	0.69	380	0.44%	0.73	357	0.84%	0.65	80	0.39%	0.07	42	0.47%	0.08	38	0.33%	0.07
Oesophagus	365	0.28%	0.34	155	0.18%	0.30	210	0.49%	0.38	171	0.83%	0.16	54	0.60%	0.10	117	1.01%	0.21
Oropharynx	102	0.08%	0.10	28	0.03%	0.05	74	0.17%	0.13	58	0.28%	0.05	23	0.25%	0.04	35	0.30%	0.06
Ovary	2110	1.64%	4.06	2110	2.44%	4.06	0	0	0.00	398	1.93%	0.77	398	4.41%	0.77	0	0	0.00
Pancreas	737	0.57%	0.69	271	0.31%	0.52	466	1.09%	0.85	465	2.26%	0.44	148	1.64%	0.28	317	2.74%	0.58
Penis	104	0.08%	0.19	0	0	0.00	104	0.24%	0.19	31	0.15%	0.06	0	0	0.00	31	0.27%	0.06

Prostate	59	0.05%	0.11	0	0	0.00	59	0.14%	0.11	29	0.14%	0.05	0	0	0.00	29	0.25%	0.05
Salivary glands	401	0.31%	0.38	237	0.27%	0.46	164	0.39%	0.30	64	0.31%	0.06	24	0.27%	0.05	40	0.35%	0.07
Stomach	2994	2.32%	2.80	1483	1.72%	2.85	1511	3.55%	2.75	1477	7.18%	1.38	714	7.92%	1.37	763	6.60%	1.39
Testis	355	0.28%	0.65	0	0	0.00	355	0.83%	0.65	31	0.15%	0.06	0	0	0.00	31	0.27%	0.06
Thyroid	55658	43.18%	52.09	38317	44.38%	73.76	17341	40.74%	31.59	236	1.15%	0.22	144	1.60%	0.28	92	0.80%	0.17
Trachea, bronchus and lung	8837	6.86%	8.27	5184	6.00%	9.98	3653	8.58%	6.65	2702	13.13%	2.53	1036	11.49%	1.99	1666	14.41%	3.03
Vagina	58	0.04%	0.11	58	0.07%	0.11	0	0	0.00	9	0.04%	0.02	9	0.10%	0.02	0	0	0.00
Vulva	88	0.07%	0.17	88	0.10%	0.17	0	0	0.00	23	0.11%	0.04	23	0.25%	0.04	0	0	0.00

Table S7: Estimated absolute number, proportion, and ASRs of incidence and mortality by cancer type and sex, among early-onset cancers aged 40 to 44 years worldwide in 2022.

Cancer type	Estimated new cancer cases and ASR per 100,000 people per year									Estimated cancer-related deaths and ASR per 100,000 people per year								
	Both sex			Female			Male			Both sex			Female			Male		
	Cases	(%)	ASR	Cases	(%)	ASR	Cases	(%)	ASR	Cases	(%)	ASR	Cases	(%)	ASR	Cases	(%)	ASR
All cancers	167624	100%	180.66	112108	100%	245.85	55516	100%	118.76	37202	100%	40.91	15967	100%	35.97	21235	100%	45.60
Bladder	1261	0.75%	1.31	314	0.28%	0.67	947	1.71%	1.92	177	0.48%	0.18	29	0.18%	0.06	148	0.70%	0.30
Brain, central nervous system	3531	2.11%	3.67	1819	1.62%	3.88	1712	3.08%	3.47	1696	4.56%	1.76	702	4.40%	1.50	994	4.68%	2.01
Breast	29963	17.88%	63.93	29963	26.73%	63.93	0	0	0.00	3323	8.93%	7.09	3323	20.81%	7.09	0	0	0.00
Cervix uteri	12416	7.41%	26.49	12416	11.08%	26.49	0	0	0.00	2468	6.63%	5.27	2468	15.46%	5.27	0	0	0.00
Colorectum	10194	6.08%	10.59	4465	3.98%	9.53	5729	10.32%	11.60	2803	7.53%	2.91	1197	7.50%	2.55	1606	7.56%	3.25
Corpus uteri	3814	2.28%	8.14	3814	3.40%	8.14	0	0	0.00	277	0.74%	0.59	277	1.73%	0.59	0	0	0.00
Gallbladder	319	0.19%	0.33	178	0.16%	0.38	141	0.25%	0.29	164	0.44%	0.17	93	0.58%	0.20	71	0.33%	0.14
Hodgkin lymphoma	203	0.12%	0.21	67	0.06%	0.14	136	0.24%	0.28	51	0.14%	0.05	21	0.13%	0.04	30	0.14%	0.06
Hypopharynx	143	0.09%	0.15	14	0.01%	0.03	129	0.23%	0.26	71	0.19%	0.07	0	0.00%	0.00	71	0.33%	0.14
Kaposi sarcoma	21	0.01%	0.02	11	0.01%	0.02	10	0.02%	0.02	3	0.01%	0.00	0	0.00%	0.00	3	0.01%	0.01
Kidney	2620	1.56%	2.72	861	0.77%	1.84	1759	3.17%	3.56	284	0.76%	0.30	92	0.58%	0.20	192	0.90%	0.39
Larynx	288	0.17%	0.30	27	0.02%	0.06	261	0.47%	0.53	116	0.31%	0.12	6	0.04%	0.01	110	0.52%	0.22
Leukaemia	2932	1.75%	3.05	1335	1.19%	2.85	1597	2.88%	3.23	1340	3.60%	1.39	579	3.63%	1.24	761	3.58%	1.54
Lip, oral cavity	1038	0.62%	1.08	335	0.30%	0.71	703	1.27%	1.42	347	0.93%	0.36	99	0.62%	0.21	248	1.17%	0.50
Liver and intrahepatic bile ducts	13130	7.83%	13.64	2054	1.83%	4.38	11076	19.95%	22.44	9844	26.46%	10.23	1314	8.23%	2.80	8530	40.17%	17.28
Melanoma of skin	251	0.15%	0.26	119	0.11%	0.25	132	0.24%	0.27	110	0.30%	0.11	53	0.33%	0.11	57	0.27%	0.12
Mesothelioma	45	0.03%	0.05	26	0.02%	0.06	19	0.03%	0.04	22	0.06%	0.02	7	0.04%	0.01	15	0.07%	0.03
Multiple myeloma	535	0.32%	0.56	225	0.20%	0.48	310	0.56%	0.63	206	0.55%	0.21	93	0.58%	0.20	113	0.53%	0.23
Nasopharynx	3855	2.30%	4.01	1087	0.97%	2.32	2768	4.99%	5.61	1081	2.91%	1.12	284	1.78%	0.61	797	3.75%	1.61
Non-Hodgkin lymphoma	2556	1.52%	2.66	1157	1.03%	2.47	1399	2.52%	2.83	715	1.92%	0.74	257	1.61%	0.55	458	2.16%	0.93
Non-melanoma skin cancer	865	0.52%	0.90	375	0.33%	0.80	490	0.88%	0.99	144	0.39%	0.15	56	0.35%	0.12	88	0.41%	0.18
Oesophagus	1184	0.71%	1.23	259	0.23%	0.55	925	1.67%	1.87	742	1.99%	0.77	121	0.76%	0.26	621	2.92%	1.26
Oropharynx	231	0.14%	0.24	50	0.04%	0.11	181	0.33%	0.37	69	0.19%	0.07	6	0.04%	0.01	63	0.30%	0.13
Ovary	3446	2.06%	7.35	3446	3.07%	7.35	0	0	0.00	937	2.52%	2.00	937	5.87%	2.00	0	0	0.00
Pancreas	1540	0.92%	1.60	525	0.47%	1.12	1015	1.83%	2.06	1125	3.02%	1.17	385	2.41%	0.82	740	3.48%	1.50
Penis	129	0.08%	0.26	0	0	0.00	129	0.23%	0.26	27	0.07%	0.05	0	0	0.00	27	0.13%	0.05

Prostate	180	0.11%	0.36	0	0	0.00	180	0.32%	0.36	44	0.12%	0.09	0	0	0.00	44	0.21%	0.09
Salivary glands	508	0.30%	0.53	272	0.24%	0.58	236	0.43%	0.48	56	0.15%	0.06	31	0.19%	0.07	25	0.12%	0.05
Stomach	5034	3.00%	5.23	2289	2.04%	4.88	2745	4.94%	5.56	2680	7.20%	2.78	1088	6.81%	2.32	1592	7.50%	3.22
Testis	305	0.18%	0.62	0	0	0.00	305	0.55%	0.62	51	0.14%	0.10	0	0	0.00	51	0.24%	0.10
Thyroid	49230	29.37%	51.15	35672	31.82%	76.11	13558	24.42%	27.46	306	0.82%	0.32	197	1.23%	0.42	109	0.51%	0.22
Trachea, bronchus and lung	15617	9.32%	16.23	8693	7.75%	18.55	6924	12.47%	14.03	5872	15.78%	6.10	2201	13.78%	4.70	3671	17.29%	7.44
Vagina	86	0.05%	0.18	86	0.08%	0.18	0	0	0.00	10	0.03%	0.02	10	0.06%	0.02	0	0	0.00
Vulva	154	0.09%	0.33	154	0.14%	0.33	0	0	0.00	41	0.11%	0.09	41	0.26%	0.09	0	0	0.00

Table S8: Estimated absolute number, proportion, and ASRs of incidence and mortality by cancer type and sex, among early-onset cancers aged 45 to 49 years worldwide in 2022.

Cancer type	Estimated new cancer cases and ASR per 100,000 people per year									Estimated cancer-related deaths and ASR per 100,000 people per year								
	Both sex			Female			Male			Both sex			Female			Male		
	Cases	(%)	ASR	Cases	(%)	ASR	Cases	(%)	ASR	Cases	(%)	ASR	Cases	(%)	ASR	Cases	(%)	ASR
All cancers	274911	100%	277.54	175386	100%	357.80	99525	100%	200.44	77448	100%	79.32	31769	100%	66.38	45679	100%	91.76
Bladder	2419	0.88%	2.35	474	0.27%	0.94	1945	1.95%	3.70	405	0.52%	0.39	99	0.31%	0.20	306	0.67%	0.58
Brain, central nervous system	5662	2.06%	5.50	2804	1.60%	5.56	2858	2.87%	5.44	2878	3.72%	2.79	1166	3.67%	2.31	1712	3.75%	3.26
Breast	49686	18.07%	98.46	49686	28.33%	98.46	0	0	0.00	6259	8.08%	12.40	6259	19.70%	12.40	0	0	0.00
Cervix uteri	18927	6.88%	37.51	18927	10.79%	37.51	0	0	0.00	4504	5.82%	8.93	4504	14.18%	8.93	0	0	0.00
Colorectum	20691	7.53%	20.09	8653	4.93%	17.15	12038	12.10%	22.92	6189	7.99%	6.01	2501	7.87%	4.96	3688	8.07%	7.02
Corpus uteri	8561	3.11%	16.97	8561	4.88%	16.97	0	0	0.00	680	0.88%	1.35	680	2.14%	1.35	0	0	0.00
Gallbladder	844	0.31%	0.82	548	0.31%	1.09	296	0.30%	0.56	534	0.69%	0.52	347	1.09%	0.69	187	0.41%	0.36
Hodgkin lymphoma	214	0.08%	0.21	79	0.05%	0.16	135	0.14%	0.26	65	0.08%	0.06	20	0.06%	0.04	45	0.10%	0.09
Hypopharynx	477	0.17%	0.46	21	0.01%	0.04	456	0.46%	0.87	254	0.33%	0.25	7	0.02%	0.01	247	0.54%	0.47
Kaposi sarcoma	35	0.01%	0.03	16	0.01%	0.03	19	0.02%	0.04	21	0.03%	0.02	17	0.05%	0.03	4	0.01%	0.01
Kidney	4645	1.69%	4.51	1554	0.89%	3.08	3091	3.11%	5.88	681	0.88%	0.66	216	0.68%	0.43	465	1.02%	0.89
Larynx	1015	0.37%	0.99	83	0.05%	0.16	932	0.94%	1.77	367	0.47%	0.36	23	0.07%	0.05	344	0.75%	0.65
Leukaemia	4286	1.56%	4.16	1880	1.07%	3.73	2406	2.42%	4.58	2120	2.74%	2.06	871	2.74%	1.73	1249	2.73%	2.38
Lip, oral cavity	1960	0.71%	1.90	600	0.34%	1.19	1360	1.37%	2.59	685	0.88%	0.67	159	0.50%	0.32	526	1.15%	1.00
Liver and intrahepatic bile ducts	23765	8.64%	23.08	3716	2.12%	7.36	20049	20.14%	38.17	18086	23.35%	17.56	2625	8.26%	5.20	15461	33.85%	29.43
Melanoma of skin	404	0.15%	0.39	226	0.13%	0.45	178	0.18%	0.34	210	0.27%	0.20	87	0.27%	0.17	123	0.27%	0.23
Mesothelioma	108	0.04%	0.10	58	0.03%	0.11	50	0.05%	0.10	65	0.08%	0.06	28	0.09%	0.06	37	0.08%	0.07
Multiple myeloma	1160	0.42%	1.13	514	0.29%	1.02	646	0.65%	1.23	479	0.62%	0.47	185	0.58%	0.37	294	0.64%	0.56
Nasopharynx	5707	2.08%	5.54	1587	0.90%	3.14	4120	4.14%	7.84	2031	2.62%	1.97	389	1.22%	0.77	1642	3.59%	3.13
Non-Hodgkin lymphoma	4103	1.49%	3.98	1821	1.04%	3.61	2282	2.29%	4.34	1311	1.69%	1.27	482	1.52%	0.96	829	1.81%	1.58
Non-melanoma skin cancer	1579	0.57%	1.53	712	0.41%	1.41	867	0.87%	1.65	231	0.30%	0.22	97	0.31%	0.19	134	0.29%	0.26
Oesophagus	4013	1.46%	3.90	662	0.38%	1.31	3351	3.37%	6.38	2520	3.25%	2.45	295	0.93%	0.58	2225	4.87%	4.24
Oropharynx	475	0.17%	0.46	108	0.06%	0.21	367	0.37%	0.70	205	0.26%	0.20	26	0.08%	0.05	179	0.39%	0.34
Ovary	6479	2.36%	12.84	6479	3.69%	12.84	0	0	0.00	2137	2.76%	4.23	2137	6.73%	4.23	0	0	0.00
Pancreas	3640	1.32%	3.53	1307	0.75%	2.59	2333	2.34%	4.44	2729	3.52%	2.65	905	2.85%	1.79	1824	3.99%	3.47
Penis	346	0.13%	0.66	0	0	0.00	346	0.35%	0.66	90	0.12%	0.17	0	0	0.00	90	0.20%	0.17

Prostate	382	0.14%	0.73	0	0	0.00	382	0.38%	0.73	130	0.17%	0.25	0	0	0.00	130	0.28%	0.25
Salivary glands	783	0.28%	0.76	379	0.22%	0.75	404	0.41%	0.77	148	0.19%	0.14	33	0.10%	0.07	115	0.25%	0.22
Stomach	11310	4.11%	10.98	4441	2.53%	8.80	6869	6.90%	13.08	5522	7.13%	5.36	2008	6.32%	3.98	3514	7.69%	6.69
Testis	252	0.09%	0.48	0	0	0.00	252	0.25%	0.48	36	0.05%	0.07	0	0	0.00	36	0.08%	0.07
Thyroid	55319	20.12%	53.71	42188	24.05%	83.60	13131	13.19%	25.00	550	0.71%	0.53	380	1.20%	0.75	170	0.37%	0.32
Trachea, bronchus and lung	35227	12.81%	34.20	16865	9.62%	33.42	18362	18.45%	34.96	15212	19.64%	14.77	5109	16.08%	10.12	10103	22.12%	19.23
Vagina	225	0.08%	0.45	225	0.13%	0.45	0	0	0.00	59	0.08%	0.12	59	0.19%	0.12	0	0	0.00
Vulva	212	0.08%	0.42	212	0.12%	0.42	0	0	0.00	55	0.07%	0.11	55	0.17%	0.11	0	0	0.00

Table S9: The incidence, mortality, and DALYs number for 32 early-onset cancers in 1990 and 2021 in China, with TPC from 1990 to 2021.

Early-onset cancers	Incidence			Mortality			DALYs		
	1990, number (95% UI)	2021, number (95% UI)	TPC (95% UI)	1990, number (95% UI)	2021, number (95% UI)	TPC (95% UI)	1990, number (95% UI)	2021, number (95% UI)	TPC (95% UI)
Bladder cancer	5171.77 (3330.25 to 6183.78)	8954.02 (7107.38 to 11395.65)	0.73 (0.24 to 1.73)	1916.24 (1241.55 to 2292.35)	1575.37 (1245.91 to 2021.21)	-0.18 (-0.41 to 0.30)	98783.34 (64433.31 to 117820.62)	81538.01 (64580.16 to 105145.41)	-0.17 (-0.41 to 0.29)
Brain and central nervous system cancer	15577.47 (11296.49 to 19639.05)	29948.84 (23093.84 to 38687.17)	0.92 (0.47 to 1.55)	11447.91 (8298.56 to 14451.44)	13265.25 (10144.75 to 17609.82)	0.16 (-0.12 to 0.56)	642093.74 (464947.43 to 810270.44)	703728.67 (538661.53 to 929730.68)	0.10 (-0.17 to 0.48)
Breast cancer	37250.13 (29629.00 to 45889.01)	113675.86 (85742.51 to 146342.50)	2.05 (1.13 to 3.35)	13747.94 (10931.74 to 16954.95)	16842.52 (12653.74 to 21851.70)	0.23 (-0.14 to 0.72)	708965.53 (563186.60 to 875807.64)	883688.30 (669817.95 to 1143992.58)	0.25 (-0.12 to 0.77)
Cervical cancer	28243.67 (22277.05 to 35813.10)	49796.24 (35518.21 to 65634.10)	0.76 (0.14 to 1.55)	10020.35 (7890.43 to 12628.33)	9212.38 (6630.27 to 12141.72)	-0.08 (-0.41 to 0.34)	516820.44 (407041.90 to 652148.23)	467647.35 (335980.55 to 621267.21)	-0.10 (-0.42 to 0.31)
Colon and rectum cancer	35027.26 (29409.35 to 40729.02)	78692.27 (62703.11 to 96467.59)	1.25 (0.68 to 1.97)	22619.46 (18985.71 to 26252.24)	24475.98 (19469.18 to 29983.10)	0.08 (-0.20 to 0.44)	1177805.62 (988155.01 to 1365275.58)	1243552.26 (983058.92 to 1524383.13)	0.06 (-0.22 to 0.40)
Esophageal cancer	25380.20 (21029.05 to 30165.06)	18898.87 (14639.67 to 23588.69)	-0.26 (-0.46 to 0.01)	22353.26 (18486.33 to 26566.59)	13002.25 (10029.61 to 16498.22)	-0.42 (-0.59 to -0.21)	1064842.65 (883199.21 to 1262305.88)	605781.12 (467322.02 to 767302.91)	-0.43 (-0.59 to -0.23)
Eye cancer	320.19 (182.17 to 475.19)	502.48 (268.10 to 718.08)	0.57 (-0.04 to 1.33)	63.09 (36.50 to 88.20)	57.32 (31.52 to 77.98)	-0.09 (-0.43 to 0.28)	3527.71 (2033.35 to 4961.64)	3197.54 (1723.54 to 4360.50)	-0.09 (-0.43 to 0.26)
Gallbladder and biliary tract cancer	2034.69 (1359.13 to 2459.96)	3893.24 (2417.46 to 5088.76)	0.91 (0.40 to 1.69)	1730.99 (1159.43 to 2089.77)	1946.30 (1260.37 to 2533.62)	0.12 (-0.18 to 0.59)	85135.42 (56634.68 to 102741.63)	93389.47 (60237.36 to 121377.36)	0.10 (-0.20 to 0.56)

Hodgkin lymphoma	1803.53 (809.20 to 2505.12)	1261.64 (820.26 to 1752.76)	-0.30 (-0.55 to 0.26)	1495.53 (674.52 to 2070.30)	439.55 (295.71 to 645.00)	-0.71 (-0.81 to -0.45)	87219.47 (39415.34 to 121046.42)	24260.47 (16385.19 to 35497.89)	-0.72 (-0.82 to -0.48)
Kidney cancer	3828.41 (3300.02 to 4377.48)	13311.09 (10774.83 to 16361.77)	2.48 (1.69 to 3.55)	1479.83 (1277.36 to 1693.92)	2525.61 (2022.24 to 3143.77)	0.71 (0.29 to 1.26)	78412.34 (67764.09 to 89371.37)	130240.08 (104403.83 to 160660.79)	0.66 (0.27 to 1.18)
Larynx cancer	1893.81 (1487.65 to 2239.28)	2808.79 (2161.50 to 3571.79)	0.48 (0.09 to 1.02)	1360.38 (1077.53 to 1615.66)	1067.17 (814.75 to 1363.99)	-0.22 (-0.43 to 0.07)	66535.11 (52723.84 to 78744.86)	51378.95 (39367.91 to 65345.97)	-0.23 (-0.43 to 0.05)
Leukemia	27056.35 (20514.39 to 31571.22)	27613.32 (19729.74 to 34642.05)	0.02 (-0.18 to 0.31)	24148.91 (18416.91 to 28328.83)	15483.42 (11295.37 to 19418.26)	-0.36 (-0.48 to -0.18)	1445520.29 (1107868.76 to 1693283.05)	870518.84 (632670.97 to 1089099.38)	-0.40 (-0.51 to -0.23)
Lip and oral cavity cancer	3242.42 (2753.55 to 3767.12)	7606.62 (6074.97 to 9326.16)	1.35 (0.76 to 2.06)	1626.95 (1383.64 to 1901.23)	2061.74 (1620.23 to 2573.27)	0.27 (-0.07 to 0.68)	83279.71 (70926.23 to 97304.70)	102460.20 (80366.64 to 126923.80)	0.23 (-0.09 to 0.62)
Liver cancer	30185.39 (24838.63 to 36227.37)	38508.72 (30544.44 to 50232.43)	0.28 (-0.09 to 0.73)	27360.02 (22538.10 to 32769.07)	28127.08 (22350.59 to 36519.34)	0.03 (-0.26 to 0.40)	1375710.47 (1134928.34 to 1647715.69)	1363072.15 (1083216.43 to 1769073.99)	-0.01 (-0.29 to 0.35)
Malignant neoplasm of bone and articular cartilage	2174.56 (1399.09 to 3891.83)	5467.63 (3441.77 to 7212.05)	1.51 (0.12 to 3.83)	1627.84 (1038.20 to 2948.65)	2499.26 (1590.49 to 3322.58)	0.54 (-0.31 to 1.98)	99362.75 (63418.16 to 180134.06)	139194.51 (88941.27 to 184472.33)	0.40 (-0.37 to 1.69)
Malignant skin melanoma	1228.45 (695.03 to 1536.95)	3744.52 (2019.21 to 5105.93)	2.05 (0.84 to 3.80)	811.85 (452.54 to 1006.11)	924.53 (504.46 to 1246.29)	0.14 (-0.31 to 0.70)	42813.05 (23732.72 to 53060.37)	48104.09 (26363.04 to 64849.48)	0.12 (-0.32 to 0.69)
Mesothelioma	337.75 (282.39 to 404.81)	556.79 (450.76 to 683.36)	0.65 (0.21 to 1.21)	303.55 (254.75 to 364.95)	484.59 (393.77 to 594.27)	0.60 (0.16 to 1.13)	15706.68 (13228.54 to 18872.59)	23842.53 (19406.51 to 28967.17)	0.52 (0.10 to 1.02)
Multiple myeloma	296.57 (210.39	1915.95 (1098.97	5.46	243.93 (173.05	1127.24 (657.19	3.62 (0.91	12826.38 (9134.02	57169.93 (32818.13	3.46 (0.84

	to 572.33)	to 2615.56)	(1.62 to 10.21)	to 468.55)	to 1492.43)	to 6.87)	to 24422.07)	to 75929.94)	to 6.57)
Nasopharynx cancer	18367.90 (15562.83 to 21245.57)	27074.84 (21164.90 to 33893.93)	0.47 (0.10 to 0.97)	12068.18 (10231.36 to 13881.84)	6091.24 (4748.13 to 7657.29)	-0.50 (-0.62 to -0.33)	629317.06 (533115.62 to 722885.24)	312022.40 (243977.97 to 389844.66)	-0.50 (-0.62 to -0.34)
Neuroblastoma and other peripheral nervous cell tumors	91.52 (64.11 to 132.40)	427.74 (327.41 to 531.32)	3.67 (2.09 to 5.70)	56.76 (42.91 to 75.69)	205.11 (160.84 to 251.57)	2.61 (1.45 to 3.98)	3323.84 (2508.89 to 4457.21)	11122.99 (8854.11 to 13582.84)	2.35 (1.27 to 3.58)
Non-Hodgkin lymphoma	9304.06 (7835.19 to 11074.85)	21570.75 (16651.67 to 26494.40)	1.32 (0.77 to 2.03)	6369.26 (5344.18 to 7572.06)	6193.18 (4782.49 to 7597.56)	-0.03 (-0.26 to 0.28)	357398.97 (300375.23 to 424511.70)	333048.87 (256051.80 to 410064.74)	-0.07 (-0.29 to 0.23)
Non-melanoma skin cancer	12672.79 (9532.48 to 15882.46)	102865.50 (77827.70 to 131602.12)	7.12 (5.79 to 8.35)	768.52 (627.23 to 955.96)	1042.39 (808.00 to 1300.57)	0.36 (0.02 to 0.76)	39953.65 (32561.00 to 49717.35)	52679.21 (40945.40 to 65415.27)	0.32 (-0.00 to 0.70)
Other pharynx cancer	861.11 (703.89 to 1040.21)	1474.82 (1158.64 to 1863.58)	0.71 (0.21 to 1.35)	618.83 (504.57 to 752.77)	492.64 (379.65 to 628.77)	-0.20 (-0.44 to 0.11)	29967.66 (24541.90 to 36185.88)	23562.11 (18322.71 to 29895.20)	-0.21 (-0.44 to 0.09)
Ovarian cancer	8587.93 (5020.09 to 11724.89)	11339.35 (8278.88 to 15199.54)	0.32 (-0.22 to 1.49)	2913.12 (1760.64 to 3975.12)	3531.77 (2553.11 to 4734.87)	0.21 (-0.29 to 1.18)	153874.33 (91308.47 to 209935.53)	176403.44 (126934.17 to 236354.71)	0.15 (-0.33 to 1.11)
Pancreatic cancer	6240.82 (5256.33 to 7386.02)	10301.58 (7944.13 to 12878.47)	0.65 (0.18 to 1.25)	5687.11 (4791.69 to 6710.96)	8887.05 (6861.73 to 11111.82)	0.56 (0.12 to 1.14)	280905.18 (236991.31 to 330410.10)	424576.18 (327789.24 to 529790.05)	0.51 (0.08 to 1.05)
Prostate cancer	694.16 (318.38 to 962.33)	2388.81 (1623.76 to 3239.23)	2.44 (1.19 to 5.21)	340.22 (154.97 to 468.03)	378.13 (256.17 to 515.22)	0.11 (-0.29 to 1.03)	17522.94 (7738.68 to 24324.66)	19831.03 (13138.29 to 26823.41)	0.13 (-0.28 to 1.09)
Soft tissue and other extraosseous sarcomas	1660.94 (1152.28 to	1591.14 (1106.12 to 2361.56)	-0.04 (-0.28 to	887.36 (621.51 to 1163.20)	568.75 (399.16 to 841.80)	-0.36 (-0.52 to	49395.11 (34839.15 to 64941.33)	29900.69 (20983.65 to 44371.86)	-0.39 (-0.54 to

	2176.07)		0.30)			-0.12)			-0.17)
Stomach cancer	70248.17 (55605.96 to 82397.39)	58119.32 (45912.46 to 74437.82)	-0.17 (-0.36 to 0.09)	54445.40 (43552.93 to 63913.66)	30568.44 (23988.19 to 39506.28)	-0.44 (-0.57 to -0.26)	2691979.23 (2159191.48 to 3152675.38)	1480083.63 (1170131.75 to 1899621.21)	-0.45 (-0.57 to -0.28)
Testicular cancer	1385.67 (1147.31 to 1638.66)	4223.68 (3339.86 to 5316.18)	2.05 (1.20 to 3.15)	686.06 (565.05 to 809.62)	440.74 (339.74 to 555.31)	-0.36 (-0.54 to -0.13)	40535.32 (33470.21 to 47850.13)	26723.29 (20940.14 to 33347.94)	-0.34 (-0.53 to -0.10)
Thyroid cancer	5571.71 (4352.45 to 6775.99)	17157.21 (13671.59 to 22388.64)	2.08 (1.32 to 3.44)	628.31 (507.59 to 749.26)	649.31 (514.54 to 817.44)	0.03 (-0.21 to 0.42)	35830.53 (28752.46 to 43774.80)	40821.60 (31637.28 to 51978.82)	0.14 (-0.12 to 0.59)
Tracheal, bronchus, and lung cancer	35889.78 (30752.73 to 41673.33)	52900.59 (41336.18 to 65169.38)	0.47 (0.10 to 0.94)	32520.16 (27890.38 to 37748.00)	40816.15 (31721.25 to 50082.77)	0.26 (-0.08 to 0.67)	1603076.79 (1374282.58 to 1855486.15)	1940033.22 (1512282.18 to 2377447.05)	0.21 (-0.11 to 0.60)
Uterine cancer	7608.80 (4290.70 to 10082.08)	14312.09 (9842.02 to 20370.33)	0.88 (0.26 to 2.38)	2254.96 (1230.52 to 3004.57)	1597.36 (1109.79 to 2200.70)	-0.29 (-0.52 to 0.27)	115737.06 (62139.62 to 154752.10)	83645.38 (57606.29 to 117195.51)	-0.28 (-0.52 to 0.31)

TPC=total percentage change.

Table S10: The incidence, mortality, and DALYs number for 30 female early-onset cancers in 1990 and 2021 in China, with TPC from 1990 to 2021.

Early-onset cancers	Incidence			Mortality			DALYs		
	1990, number (95% UI)	2021, number (95% UI)	TPC (95% UI)	1990, number (95% UI)	2021, number (95% UI)	TPC (95% UI)	1990, number (95% UI)	2021, number (95% UI)	TPC (95% UI)
Bladder cancer	1346.09 (946.08 to 1738.75)	1517.31 (1083.36 to 2064.43)	0.13 (-0.29 to 0.78)	528.55 (375.49 to 673.34)	289.51 (207.91 to 393.72)	-0.45 (-0.65 to -0.14)	27745.12 (19752.73 to 35411.68)	15000.79 (10720.87 to 20258.60)	-0.46 (-0.66 to -0.14)
Brain and central nervous system cancer	7200.16 (5225.19 to 9320.97)	13722.30 (10224.92 to 18969.51)	0.91 (0.32 to 1.83)	4904.07 (3531.02 to 6313.95)	5051.01 (3742.15 to 6964.93)	0.03 (-0.29 to 0.53)	274320.08 (198292.10 to 353594.78)	268516.15 (198116.93 to 369088.12)	-0.02 (-0.33 to 0.45)
Breast cancer	36688.89 (29142.12 to 45382.59)	110371.41 (82542.87 to 142344.82)	2.01 (1.10 to 3.32)	13566.95 (10747.38 to 16802.55)	16439.33 (12282.09 to 21419.56)	0.21 (-0.15 to 0.72)	699470.16 (554280.94 to 866932.80)	861866.60 (648610.17 to 1119103.01)	0.23 (-0.14 to 0.75)
Cervical cancer	28243.67 (22277.05 to 35813.10)	49796.24 (35518.21 to 65634.10)	0.76 (0.14 to 1.55)	10020.35 (7890.43 to 12628.33)	9212.38 (6630.27 to 12141.72)	-0.08 (-0.41 to 0.34)	516820.44 (407041.90 to 652148.23)	467647.35 (335980.55 to 621267.21)	-0.10 (-0.42 to 0.31)
Colon and rectum cancer	14229.93 (11034.14 to 18060.28)	22766.72 (16569.47 to 30498.39)	0.60 (0.06 to 1.41)	9022.02 (6987.69 to 11474.79)	6667.39 (4831.89 to 8938.14)	-0.26 (-0.50 to 0.11)	471928.65 (364596.07 to 600389.14)	338342.95 (244200.13 to 455143.39)	-0.28 (-0.52 to 0.08)
Esophageal cancer	3978.78 (1854.40 to 5183.11)	2198.10 (1579.48 to 3002.90)	-0.45 (-0.64 to 0.09)	3262.95 (1538.84 to 4245.62)	1145.63 (839.12 to 1569.66)	-0.65 (-0.77 to -0.30)	156067.55 (74794.54 to 203327.86)	54132.17 (39667.04 to 74024.47)	-0.65 (-0.77 to -0.32)
Eye cancer	146.69 (74.35 to 243.51)	227.01 (122.34 to 360.76)	0.55 (-0.28 to 1.63)	28.47 (14.15 to 46.14)	25.39 (14.35 to 39.06)	-0.11 (-0.58 to 0.47)	1604.41 (797.09 to 2638.01)	1429.93 (796.80 to 2234.96)	-0.11 (-0.58 to 0.47)
Gallbladder and biliary tract cancer	873.81 (513.07 to 1180.78)	1278.60 (798.59 to 1823.68)	0.46 (-0.05 to 1.56)	753.34 (440.39 to 1018.20)	676.19 (427.85 to 974.67)	-0.10 (-0.41 to 0.55)	37042.48 (21573.58 to 50123.05)	32032.89 (20276.86 to 46120.44)	-0.14 (-0.43 to 0.49)
Hodgkin lymphoma	686.91 (240.20 to 1012.15)	465.33 (272.38 to 695.10)	-0.32 (-0.59 to 0.52)	558.75 (195.38 to 819.18)	145.32 (89.15 to 219.95)	-0.74 (-0.84 to -0.41)	32832.49 (11529.56 to 48221.60)	8109.55 (4989.38 to 12433.60)	-0.75 (-0.85 to -0.44)

Kidney cancer	1224.62 (950.45 to 1574.78)	3124.58 (2205.44 to 4237.42)	1.55 (0.63 to 2.86)	415.33 (322.11 to 528.23)	467.24 (331.43 to 644.88)	0.12 (-0.27 to 0.69)	23047.41 (17924.73 to 29268.40)	25067.27 (17890.00 to 34255.52)	0.09 (-0.30 to 0.64)
Larynx cancer	329.60 (137.05 to 446.94)	454.33 (291.29 to 670.38)	0.38 (-0.11 to 2.24)	232.31 (94.71 to 313.88)	154.75 (97.54 to 222.97)	-0.33 (-0.57 to 0.53)	11855.91 (4773.26 to 16016.23)	7699.83 (4847.16 to 11039.09)	-0.35 (-0.58 to 0.51)
Leukemia	12064.73 (8348.74 to 15250.56)	10412.45 (6238.97 to 13943.18)	-0.14 (-0.41 to 0.23)	10742.90 (7455.02 to 13521.26)	5812.25 (3675.31 to 7829.30)	-0.46 (-0.62 to -0.22)	637382.56 (442467.41 to 802595.94)	323781.42 (203428.91 to 434383.08)	-0.49 (-0.64 to -0.28)
Lip and oral cavity cancer	1304.13 (1033.48 to 1616.49)	2175.96 (1637.00 to 2885.40)	0.67 (0.17 to 1.34)	549.47 (437.97 to 679.48)	420.94 (316.76 to 559.18)	-0.23 (-0.47 to 0.09)	29081.99 (23188.43 to 35925.31)	21573.93 (16223.47 to 28758.64)	-0.26 (-0.48 to 0.05)
Liver cancer	5475.23 (4249.99 to 6857.09)	5095.74 (3789.26 to 6741.91)	-0.07 (-0.35 to 0.39)	4971.27 (3868.40 to 6194.64)	3707.00 (2740.94 to 4845.23)	-0.25 (-0.48 to 0.11)	254628.70 (197851.05 to 317264.69)	179386.71 (132713.72 to 234924.40)	-0.30 (-0.51 to 0.05)
Malignant neoplasm of bone and articular cartilage	847.11 (492.28 to 1831.08)	1876.32 (1091.43 to 2825.19)	1.21 (-0.27 to 3.71)	633.30 (369.88 to 1393.81)	861.69 (510.40 to 1283.93)	0.36 (-0.55 to 1.92)	38283.25 (22406.99 to 84786.88)	47280.07 (28176.35 to 70509.59)	0.24 (-0.60 to 1.63)
Malignant skin melanoma	483.79 (257.04 to 699.16)	1532.29 (634.03 to 2575.96)	2.17 (0.38 to 4.61)	308.31 (161.50 to 445.11)	349.52 (142.28 to 559.60)	0.13 (-0.52 to 1.01)	16473.06 (8559.69 to 23832.81)	18227.48 (7428.11 to 29323.19)	0.11 (-0.53 to 0.95)
Mesothelioma	108.41 (76.98 to 189.09)	159.08 (109.31 to 215.52)	0.47 (-0.28 to 1.32)	100.49 (71.20 to 174.54)	145.70 (99.62 to 198.64)	0.45 (-0.29 to 1.28)	5274.95 (3724.17 to 9178.45)	7265.30 (5000.27 to 9818.22)	0.38 (-0.33 to 1.17)
Multiple myeloma	130.99 (80.13 to 316.17)	699.27 (248.05 to 1059.92)	4.34 (0.00 to 10.63)	107.84 (65.99 to 260.93)	411.39 (147.88 to 612.16)	2.81 (-0.27 to 7.18)	5622.45 (3454.10 to 13577.96)	20614.63 (7348.68 to 30808.27)	2.67 (-0.30 to 6.84)
Nasopharynx cancer	6041.26 (4653.59 to 7562.24)	6499.35 (4670.64 to 8963.52)	0.08 (-0.28 to 0.61)	3720.51 (2872.74 to 4676.99)	1287.97 (939.80 to 1739.50)	-0.65 (-0.76 to -0.49)	195476.91 (151127.27 to 245188.34)	66624.51 (48638.77 to 89704.38)	-0.66 (-0.77 to -0.50)
Neuroblastoma and other	41.80 (24.38 to	152.95 (84.62 to	2.66 (0.62	25.85 (16.20 to	73.36 (41.84 to	1.84 (0.26	1521.30 (946.53 to	3991.35 (2315.76	1.62 (0.17

peripheral nervous cell tumors	70.63)	216.61)	to 5.00)	42.23)	100.61)	to 3.55)	2485.81)	to 5465.03)	to 3.20)
Non-Hodgkin lymphoma	3729.52 (2689.05 to 4716.50)	6631.50 (4632.05 to 8952.54)	0.78 (0.24 to 1.72)	2552.73 (1834.89 to 3225.62)	1887.56 (1324.66 to 2555.46)	-0.26 (-0.49 to 0.13)	144432.70 (103681.38 to 182652.09)	101930.04 (71398.78 to 138215.49)	-0.29 (-0.51 to 0.09)
Non-melanoma skin cancer	6039.41 (4513.02 to 7669.68)	60351.51 (46105.24 to 76728.58)	8.99 (7.24 to 10.75)	371.98 (289.05 to 480.68)	504.34 (354.79 to 690.27)	0.36 (-0.14 to 0.97)	19172.50 (14921.65 to 24842.99)	25080.38 (17741.14 to 34055.82)	0.31 (-0.16 to 0.89)
Other pharynx cancer	135.31 (78.05 to 177.28)	253.58 (160.46 to 385.63)	0.87 (0.13 to 3.01)	88.81 (50.72 to 115.68)	66.97 (42.47 to 100.03)	-0.25 (-0.55 to 0.62)	4616.52 (2608.63 to 6037.73)	3383.45 (2145.51 to 5066.20)	-0.27 (-0.56 to 0.59)
Ovarian cancer	8587.93 (5020.09 to 11724.89)	11339.35 (8278.88 to 15199.54)	0.32 (-0.22 to 1.49)	2913.12 (1760.64 to 3975.12)	3531.77 (2553.11 to 4734.87)	0.21 (-0.29 to 1.18)	153874.33 (91308.47 to 209935.53)	176403.44 (126934.17 to 236354.71)	0.15 (-0.33 to 1.11)
Pancreatic cancer	1838.90 (1394.70 to 2355.01)	2466.22 (1784.65 to 3277.64)	0.34 (-0.15 to 1.02)	1671.94 (1269.47 to 2135.07)	2100.44 (1508.45 to 2778.15)	0.26 (-0.21 to 0.89)	83093.89 (62948.51 to 105838.96)	99552.93 (71310.23 to 131783.64)	0.20 (-0.25 to 0.81)
Soft tissue and other extraosseous sarcomas	798.25 (479.92 to 1107.76)	580.24 (382.13 to 943.92)	-0.27 (-0.52 to 0.28)	423.10 (256.77 to 582.07)	206.75 (136.30 to 334.43)	-0.51 (-0.68 to -0.14)	23817.91 (14494.76 to 32882.50)	10952.72 (7236.60 to 17765.11)	-0.54 (-0.69 to -0.19)
Stomach cancer	21918.67 (17060.74 to 27781.82)	14636.57 (11186.73 to 19675.66)	-0.33 (-0.52 to -0.03)	17144.74 (13373.27 to 21682.68)	7812.73 (5918.52 to 10442.71)	-0.54 (-0.67 to -0.34)	867858.81 (675768.66 to 1097848.91)	384357.86 (291588.68 to 516041.48)	-0.56 (-0.68 to -0.36)
Thyroid cancer	4323.83 (3026.04 to 5485.85)	10016.62 (7206.89 to 15596.12)	1.32 (0.56 to 2.85)	397.43 (284.22 to 507.51)	250.89 (178.96 to 388.43)	-0.37 (-0.58 to 0.03)	23020.22 (16641.04 to 29757.93)	17517.12 (12021.44 to 27142.20)	-0.24 (-0.49 to 0.31)
Tracheal, bronchus, and lung cancer	12345.70 (9625.72 to 15659.74)	19706.92 (14838.61 to 25509.37)	0.60 (0.12 to 1.29)	11042.72 (8622.05 to 13943.85)	13999.45 (10527.15 to 18082.87)	0.27 (-0.11 to 0.81)	550505.80 (429912.53 to 694976.67)	667019.91 (502304.64 to 863199.78)	0.21 (-0.14 to 0.72)
Uterine cancer	7608.80 (4290.70 to	14312.09 (9842.02 to	0.88 (0.26 to 2.38)	2254.96 (1230.52 to	1597.36 (1109.79 to	-0.29 (-0.52 to	115737.06 (62139.62 to	83645.38 (57606.29 to	-0.28 (-0.52 to

	10082.08)	20370.33)		3004.57)	2200.70)	0.27)	154752.10)	117195.51)	0.31)

TPC=total percentage change.

Table S11: The incidence, mortality, and DALYs number for 29 male early-onset cancers in 1990 and 2021 in China, with TPC from 1990 to 2021.

Early-onset cancers	Incidence			Mortality			DALYs		
	1990, cases (95% UI)	2021, cases (95% UI)	TPC 95% UI)	1990, cases (95% UI)	2021, cases (95% UI)	TPC (95% UI)	1990, cases (95% UI)	2021, cases (95% UI)	TPC (95% UI)
Bladder cancer	3825.67 (2129.55 to 4842.91)	7436.71 (5589.14 to 9922.27)	0.94 (0.27 to 2.39)	1387.69 (780.02 to 1753.88)	1285.86 (964.70 to 1732.48)	-0.07 (-0.41 to 0.63)	71038.22 (39623.16 to 89554.09)	66537.23 (49851.94 to 89090.09)	-0.06 (-0.40 to 0.64)
Brain and central nervous system cancer	8377.31 (5096.82 to 11666.37)	16226.54 (9608.57 to 22571.91)	0.94 (0.25 to 1.86)	6543.84 (4009.33 to 9075.42)	8214.24 (4950.97 to 11551.60)	0.26 (-0.20 to 0.87)	367773.66 (226481.14 to 513271.91)	435212.52 (262036.69 to 612559.13)	0.18 (-0.25 to 0.76)
Breast cancer	561.24 (358.17 to 764.29)	3304.45 (1250.72 to 4740.45)	4.89 (1.86 to 8.32)	180.99 (108.87 to 246.65)	403.19 (147.27 to 577.37)	1.23 (0.09 to 2.65)	9495.37 (5694.10 to 12935.72)	21821.70 (8056.31 to 30837.58)	1.30 (0.13 to 2.74)
Colon and rectum cancer	20797.33 (15728.19 to 25356.53)	55925.55 (41917.22 to 72753.16)	1.69 (0.89 to 2.82)	13597.44 (10319.23 to 16609.72)	17808.58 (13403.39 to 23235.48)	0.31 (-0.09 to 0.88)	705876.97 (529354.09 to 858436.31)	905209.32 (682207.06 to 1174516.85)	0.28 (-0.11 to 0.83)
Esophageal cancer	21401.42 (17590.78 to 26341.67)	16700.78 (12523.56 to 21369.93)	-0.22 (-0.46 to 0.08)	19090.31 (15704.79 to 23532.38)	11856.62 (8858.98 to 15288.00)	-0.38 (-0.57 to -0.14)	908775.11 (748399.89 to 1115572.10)	551648.95 (414821.32 to 710596.49)	-0.39 (-0.58 to -0.16)
Eye cancer	173.50 (73.63 to 275.24)	275.47 (108.47 to 432.78)	0.59 (0.06 to 1.43)	34.63 (15.26 to 49.51)	31.93 (13.16 to 50.07)	-0.08 (-0.38 to 0.36)	1923.30 (843.99 to 2748.29)	1767.61 (716.41 to 2745.38)	-0.08 (-0.37 to 0.35)
Gallbladder and biliary tract cancer	1160.88 (711.27 to 1523.04)	2614.64 (1414.33 to 3657.06)	1.25 (0.48 to 2.39)	977.65 (598.63 to 1290.48)	1270.11 (712.53 to 1783.87)	0.30 (-0.15 to 0.96)	48092.93 (29367.19 to 63295.87)	61356.58 (34445.35 to 85535.12)	0.28 (-0.16 to 0.91)
Hodgkin lymphoma	1116.62 (427.51 to 1576.91)	796.31 (458.36 to 1224.50)	-0.29 (-0.55 to 0.29)	936.77 (359.60 to 1321.49)	294.23 (179.03 to 476.12)	-0.69 (-0.81 to -0.40)	54386.98 (20765.50 to 76649.80)	16150.91 (9881.69 to 26057.27)	-0.70 (-0.82 to -0.43)
Kidney cancer	2603.79 (2138.17 to 3132.69)	10186.51 (7900.64 to 12833.14)	2.91 (1.86 to 4.30)	1064.49 (875.56 to 1283.38)	2058.38 (1597.81 to 2605.72)	0.93 (0.41 to 1.62)	55364.93 (45619.34 to 66521.26)	105172.81 (81748.41 to 131881.07)	0.90 (0.38 to 1.56)

Larynx cancer	1564.21 (1235.59 to 1927.45)	2354.47 (1743.60 to 3099.17)	0.51 (0.05 to 1.11)	1128.07 (885.80 to 1389.04)	912.42 (672.77 to 1210.45)	-0.19 (-0.44 to 0.15)	54679.19 (42871.57 to 67072.32)	43679.12 (32290.01 to 57730.17)	-0.20 (-0.44 to 0.12)
Leukemia	14991.62 (9435.20 to 19034.84)	17200.86 (10594.66 to 22847.11)	0.15 (-0.17 to 0.66)	13406.01 (8442.74 to 17049.23)	9671.17 (5982.03 to 12930.72)	-0.28 (-0.48 to 0.03)	808137.73 (507599.56 to 1024955.12)	546737.42 (336303.85 to 731490.28)	-0.32 (-0.51 to -0.03)
Lip and oral cavity cancer	1938.29 (1599.71 to 2318.81)	5430.67 (4125.63 to 7032.74)	1.80 (0.90 to 2.87)	1077.48 (883.06 to 1290.91)	1640.80 (1236.22 to 2149.82)	0.52 (0.03 to 1.08)	54197.73 (44540.31 to 64720.67)	80886.27 (61256.44 to 105296.98)	0.49 (0.01 to 1.04)
Liver cancer	24710.16 (19818.54 to 30427.06)	33412.98 (25383.73 to 44101.90)	0.35 (-0.05 to 0.91)	22388.74 (17970.98 to 27511.43)	24420.08 (18515.93 to 32240.66)	0.09 (-0.24 to 0.54)	1121081.76 (901759.79 to 1379301.44)	1183685.44 (901075.31 to 1567029.14)	0.06 (-0.26 to 0.49)
Malignant neoplasm of bone and articular cartilage	1327.45 (811.34 to 2372.49)	3591.31 (2034.00 to 5043.32)	1.71 (0.30 to 4.56)	994.54 (603.28 to 1779.24)	1637.57 (942.09 to 2279.10)	0.65 (-0.22 to 2.41)	61079.49 (37550.00 to 108155.83)	91914.44 (52992.26 to 127944.84)	0.50 (-0.28 to 2.08)
Malignant skin melanoma	744.66 (354.98 to 992.46)	2212.22 (1155.01 to 3208.77)	1.97 (0.88 to 4.25)	503.53 (233.57 to 676.21)	575.01 (306.07 to 824.41)	0.14 (-0.27 to 0.92)	26339.99 (12176.54 to 35319.67)	29876.60 (15970.78 to 42963.26)	0.13 (-0.27 to 0.91)
Mesothelioma	229.34 (176.62 to 292.44)	397.71 (307.75 to 513.76)	0.73 (0.12 to 1.44)	203.06 (156.10 to 258.38)	338.88 (261.49 to 438.61)	0.67 (0.08 to 1.34)	10431.73 (8002.72 to 13242.53)	16577.23 (12840.36 to 21303.57)	0.59 (0.04 to 1.22)
Multiple myeloma	165.58 (113.51 to 338.52)	1216.68 (607.36 to 1720.14)	6.35 (1.68 to 12.43)	136.09 (93.05 to 277.37)	715.86 (356.16 to 1019.60)	4.26 (0.93 to 8.59)	7203.93 (4942.75 to 14520.43)	36555.30 (18134.11 to 52098.82)	4.07 (0.87 to 8.20)
Nasopharynx cancer	12326.65 (9994.91 to 15075.28)	20575.49 (15483.55 to 27035.74)	0.67 (0.18 to 1.37)	8347.67 (6767.62 to 10120.21)	4803.26 (3623.61 to 6197.12)	-0.42 (-0.60 to -0.18)	433840.15 (351756.99 to 523860.20)	245397.89 (185139.49 to 316632.38)	-0.43 (-0.60 to -0.20)
Neuroblastoma and other peripheral nervous cell tumors	49.71 (34.99 to 69.40)	274.79 (200.17 to 356.97)	4.53 (2.47 to 7.34)	30.90 (23.79 to 40.11)	131.75 (99.13 to 166.81)	3.26 (1.77 to 5.15)	1802.54 (1394.65 to 2343.07)	7131.64 (5390.61 to 8960.32)	2.96 (1.59 to 4.71)
Non-Hodgkin lymphoma	5574.54	14939.25	1.68	3816.53	4305.62	0.13	212966.28	231118.83	0.09

	(4504.00 to 7311.82)	(10942.30 to 19321.70)	(0.86 to 2.85)	(3080.77 to 5017.08)	(3148.47 to 5606.00)	(-0.22 to 0.61)	(172044.98 to 278584.83)	(170273.55 to 297378.69)	(-0.24 to 0.54)
Non-melanoma skin cancer	6633.38 (5035.89 to 8340.36)	42513.99 (31698.17 to 54208.80)	5.41 (4.41 to 6.31)	396.54 (283.35 to 552.08)	538.06 (349.21 to 747.42)	0.36 (-0.06 to 0.88)	20781.14 (14997.91 to 28773.90)	27598.82 (18162.51 to 37539.74)	0.33 (-0.07 to 0.84)
Other pharynx cancer	725.80 (570.21 to 910.23)	1221.25 (916.00 to 1579.66)	0.68 (0.10 to 1.39)	530.02 (415.19 to 666.54)	425.67 (318.02 to 553.84)	-0.20 (-0.47 to 0.15)	25351.14 (19927.64 to 31796.62)	20178.66 (15127.87 to 26207.93)	-0.20 (-0.48 to 0.13)
Pancreatic cancer	4401.92 (3613.39 to 5328.28)	7835.36 (5935.59 to 10190.01)	0.78 (0.21 to 1.49)	4015.17 (3308.36 to 4870.21)	6786.61 (5145.14 to 8852.78)	0.69 (0.15 to 1.37)	197811.29 (163705.05 to 239312.93)	325023.25 (247012.54 to 421959.58)	0.64 (0.13 to 1.29)
Prostate cancer	694.16 (318.38 to 962.33)	2388.81 (1623.76 to 3239.23)	2.44 (1.19 to 5.21)	340.22 (154.97 to 468.03)	378.13 (256.17 to 515.22)	0.11 (-0.29 to 1.03)	17522.94 (7738.68 to 24324.66)	19831.03 (13138.29 to 26823.41)	0.13 (-0.28 to 1.09)
Soft tissue and other extraosseous sarcomas	862.69 (526.25 to 1339.51)	1010.90 (636.32 to 1701.82)	0.17 (-0.17 to 0.60)	464.26 (284.06 to 723.36)	362.00 (229.77 to 625.88)	-0.22 (-0.46 to 0.08)	25577.20 (15791.77 to 40045.28)	18947.97 (12075.89 to 32594.95)	-0.26 (-0.48 to 0.02)
Stomach cancer	48329.50 (34297.45 to 59090.46)	43482.75 (32129.11 to 58720.66)	-0.10 (-0.35 to 0.24)	37300.66 (26398.86 to 45627.18)	22755.71 (16743.72 to 30876.72)	-0.39 (-0.56 to -0.16)	1824120.42 (1295146.26 to 2227244.53)	1095725.77 (805200.86 to 1479826.91)	-0.40 (-0.57 to -0.17)
Testicular cancer	1385.67 (1147.31 to 1638.66)	4223.68 (3339.86 to 5316.18)	2.05 (1.20 to 3.15)	686.06 (565.05 to 809.62)	440.74 (339.74 to 555.31)	-0.36 (-0.54 to -0.13)	40535.32 (33470.21 to 47850.13)	26723.29 (20940.14 to 33347.94)	-0.34 (-0.53 to -0.10)
Thyroid cancer	1247.88 (989.79 to 1633.25)	7140.59 (5214.73 to 9363.27)	4.72 (2.68 to 7.23)	230.88 (182.91 to 296.22)	398.42 (281.12 to 528.44)	0.73 (0.09 to 1.51)	12810.31 (10119.13 to 16472.28)	23304.48 (16399.85 to 30826.02)	0.82 (0.14 to 1.67)
Tracheal, bronchus, and lung cancer	23544.09 (18764.41 to 28524.37)	33193.67 (24184.61 to 43711.78)	0.41 (-0.08 to 1.01)	21477.44 (17114.35 to 26040.95)	26816.71 (19392.58 to 35444.28)	0.25 (-0.19 to 0.78)	1052570.99 (840924.21 to 1276291.39)	1273013.30 (922064.38 to 1674771.73)	0.21 (-0.21 to 0.71)

TPC=total percentage change.

Table S12: The ASR of incidence, mortality, and DALYs for 30 female early-onset cancers in 1990 and 2021 in China, with AAPC from 1990 to 2021.

Early-onset cancers	Incidence			Mortality			DALYs		
	ASR (95 % UI), 1990	ASR (95 % UI), 2021	AAPC (95% CI)	ASR (95 % UI), 1990	ASR (95 % UI), 2021	AAPC (95% CI)	ASR (95 % UI), 1990	ASR (95 % UI), 2021	AAPC (95% CI)
Bladder cancer	0.49 (0.34 to 0.64)	0.39 (0.27 to 0.53)	-1.20 (-1.44 to -0.95)	0.20 (0.14 to 0.26)	0.07 (0.05 to 0.10)	-3.98 (-4.27 to -3.70)	10.14 (7.05 to 13.18)	3.84 (2.72 to 5.25)	-3.84 (-4.13 to -3.54)
Brain and central nervous system cancer	2.44 (1.72 to 3.17)	4.01 (2.86 to 5.62)	1.63 (1.55 to 1.72)	1.69 (1.19 to 2.19)	1.41 (1.02 to 1.95)	-0.80 (-0.89 to -0.71)	91.28 (64.68 to 118.76)	78.16 (56.79 to 108.11)	-0.72 (-0.81 to -0.63)
Breast cancer	13.81 (10.87 to 17.11)	27.25 (20.12 to 35.70)	2.16 (2.07 to 2.25)	5.14 (4.05 to 6.37)	4.02 (2.98 to 5.26)	-1.21 (-1.35 to -1.07)	260.20 (204.63 to 323.90)	214.22 (158.11 to 281.21)	-1.04 (-1.17 to -0.91)
Cervical cancer	10.25 (7.92 to 13.03)	12.75 (9.04 to 16.96)	1.41 (1.16 to 1.65)	3.77 (2.94 to 4.77)	2.27 (1.61 to 3.04)	-1.20 (-1.39 to -1.02)	190.17 (148.05 to 241.53)	117.43 (83.47 to 156.94)	-1.14 (-1.32 to -0.96)
Colon and rectum cancer	5.24 (4.01 to 6.68)	5.75 (4.14 to 7.77)	-0.06 (-0.33 to 0.21)	3.32 (2.54 to 4.23)	1.68 (1.22 to 2.26)	-2.82 (-3.06 to -2.57)	169.49 (129.55 to 216.05)	87.29 (63.17 to 117.47)	-2.77 (-3.02 to -2.51)
Esophageal cancer	1.60 (0.73 to 2.12)	0.52 (0.37 to 0.72)	-4.64 (-5.01 to -4.27)	1.32 (0.60 to 1.73)	0.27 (0.20 to 0.38)	-6.20 (-6.63 to -5.76)	61.98 (28.62 to 81.57)	13.11 (9.41 to 18.09)	-6.10 (-6.53 to -5.66)
Eye cancer	0.05 (0.02 to 0.09)	0.07 (0.03 to 0.11)	1.68 (1.34 to 2.02)	0.01 (0.00 to 0.02)	0.01 (0.00 to 0.01)	-0.95 (-1.21 to -0.70)	0.56 (0.27 to 0.94)	0.40 (0.21 to 0.62)	-0.66 (-0.93 to -0.39)
Gallbladder and biliary tract cancer	0.34 (0.20 to 0.46)	0.31 (0.19 to 0.46)	-0.26 (-0.40 to -0.12)	0.29 (0.18 to 0.40)	0.16 (0.10 to 0.24)	-2.02 (-2.12 to -1.92)	14.14 (8.39 to 19.31)	7.82 (4.90 to 11.41)	-2.02 (-2.11 to -1.92)
Hodgkin lymphoma	0.22 (0.08 to 0.33)	0.14 (0.08 to 0.23)	-1.56 (-1.87 to -1.25)	0.18 (0.06 to 0.27)	0.04 (0.03 to 0.07)	-5.10 (-5.33 to -4.87)	10.33 (3.56 to 15.29)	2.51 (1.51 to 4.02)	-5.03 (-5.27 to -4.79)
Kidney cancer	0.43 (0.32 to 0.55)	0.84 (0.59 to 1.17)	3.12 (2.72 to 3.52)	0.15 (0.11 to 0.19)	0.12 (0.09 to 0.17)	-0.03 (-0.37 to 0.32)	7.87 (6.01 to 10.16)	6.86 (4.82 to 9.44)	0.10 (-0.23 to 0.44)
Larynx cancer	0.12 (0.05 to 0.17)	0.11 (0.07 to 0.17)	-0.14 (-0.31 to 0.03)	0.09 (0.04 to 0.12)	0.04 (0.02 to 0.06)	-2.75 (-2.88 to -2.62)	4.37 (1.85 to 5.97)	1.95 (1.18 to 2.86)	-2.66 (-2.79 to -2.53)
Leukemia	3.91 (2.67 to 5.01)	3.13 (1.81 to 4.22)	-0.76 (-0.83 to -0.68)	3.46 (2.37 to 4.42)	1.76 (1.09 to 2.37)	-2.33 (-2.45 to -2.19)	198.88 (136.63 to 254.22)	102.96 (63.50 to 137.96)	-2.28 (-2.41 to -2.15)
Lip and oral cavity cancer	0.47 (0.37 to 0.60)	0.56 (0.42 to 0.76)	0.50 (0.18 to 0.81)	0.20 (0.16 to 0.26)	0.11 (0.08 to 0.14)	-2.48 (-2.75 to -2.22)	10.42 (8.21 to 13.16)	5.60 (4.15 to 7.46)	-2.40 (-2.66 to -2.13)
Liver cancer	2.05 (1.54 to	1.26 (0.92 to	-2.31 (-2.72	1.86 (1.41 to	0.92 (0.67 to	-3.02 (-3.45	92.98 (70.27 to	45.45 (33.20 to	-3.09 (-3.53

	2.60)	1.69)	to -1.89)	2.35)	1.22)	to -2.59)	117.55)	60.80)	to -2.66)
Malignant neoplasm of bone and articular cartilage	0.27 (0.15 to 0.60)	0.56 (0.32 to 0.84)	2.27 (1.44 to 3.11)	0.20 (0.11 to 0.46)	0.25 (0.15 to 0.38)	0.28 (-0.54 to 1.10)	11.88 (6.68 to 26.64)	14.77 (8.75 to 21.96)	0.21 (-0.60 to 1.02)
Malignant skin melanoma	0.17 (0.09 to 0.25)	0.41 (0.17 to 0.70)	3.40 (3.05 to 3.76)	0.11 (0.06 to 0.16)	0.09 (0.04 to 0.15)	-0.49 (-0.64 to -0.34)	5.75 (2.97 to 8.42)	4.91 (1.96 to 8.04)	-0.37 (-0.53 to -0.22)
Mesothelioma	0.04 (0.03 to 0.07)	0.04 (0.03 to 0.06)	0.41 (0.15 to 0.67)	0.04 (0.03 to 0.06)	0.04 (0.03 to 0.05)	0.37 (0.11 to 0.63)	1.86 (1.30 to 3.23)	1.93 (1.32 to 2.65)	0.37 (0.11 to 0.64)
Multiple myeloma	0.05 (0.03 to 0.12)	0.18 (0.06 to 0.28)	3.28 (2.75 to 3.81)	0.04 (0.02 to 0.10)	0.11 (0.04 to 0.16)	1.93 (1.36 to 2.50)	2.00 (1.19 to 4.92)	5.46 (1.88 to 8.32)	2.03 (1.46 to 2.60)
Nasopharynx cancer	2.16 (1.63 to 2.77)	1.74 (1.21 to 2.45)	-1.37 (-1.83 to -0.91)	1.36 (1.03 to 1.74)	0.33 (0.24 to 0.45)	-5.60 (-6.00 to -5.20)	69.68 (52.78 to 88.88)	17.45 (12.50 to 24.08)	-5.50 (-5.91 to -5.08)
Neuroblastoma and other peripheral nervous cell tumors	0.01 (0.01 to 0.02)	0.05 (0.02 to 0.07)	4.67 (4.40 to 4.94)	0.01 (0.00 to 0.01)	0.02 (0.01 to 0.03)	3.59 (3.37 to 3.80)	0.48 (0.28 to 0.82)	1.23 (0.67 to 1.77)	3.58 (3.36 to 3.80)
Non-Hodgkin lymphoma	1.27 (0.92 to 1.64)	1.80 (1.22 to 2.47)	0.88 (0.33 to 1.43)	0.87 (0.63 to 1.11)	0.52 (0.36 to 0.71)	-2.07 (-2.53 to -1.62)	47.42 (34.40 to 60.81)	29.33 (20.12 to 39.98)	-2.00 (-2.47 to -1.53)
Non-melanoma skin cancer	2.10 (1.30 to 3.16)	15.17 (10.15 to 21.94)	4.38 (3.56 to 5.22)	0.14 (0.11 to 0.18)	0.13 (0.09 to 0.17)	0.40 (0.11 to 0.70)	6.91 (5.32 to 8.93)	6.53 (4.48 to 8.86)	0.43 (0.13 to 0.73)
Other pharynx cancer	0.05 (0.03 to 0.07)	0.06 (0.04 to 0.10)	1.16 (0.85 to 1.47)	0.03 (0.02 to 0.04)	0.02 (0.01 to 0.03)	-2.15 (-2.30 to -2.00)	1.66 (0.95 to 2.19)	0.88 (0.56 to 1.34)	-2.04 (-2.20 to -1.89)
Ovarian cancer	3.01 (1.79 to 4.10)	3.03 (2.14 to 4.17)	-0.18 (-0.30 to -0.06)	1.09 (0.67 to 1.48)	0.88 (0.62 to 1.21)	-1.06 (-1.19 to -0.92)	55.78 (33.75 to 76.13)	45.14 (31.84 to 62.19)	-1.03 (-1.16 to -0.89)
Pancreatic cancer	0.71 (0.54 to 0.90)	0.60 (0.43 to 0.80)	-0.87 (-1.01 to -0.72)	0.64 (0.49 to 0.82)	0.51 (0.36 to 0.68)	-1.12 (-1.25 to -0.98)	31.34 (23.78 to 40.10)	24.56 (17.61 to 32.84)	-1.15 (-1.29 to -1.01)
Soft tissue and other extraosseous sarcomas	0.27 (0.16 to 0.39)	0.16 (0.10 to 0.27)	-1.81 (-1.95 to -1.68)	0.15 (0.09 to 0.21)	0.06 (0.04 to 0.09)	-3.38 (-3.56 to -3.20)	7.95 (4.69 to 11.51)	3.15 (2.00 to 5.15)	-3.34 (-3.52 to -3.16)
Stomach cancer	8.20 (6.36 to 10.46)	3.73 (2.78 to 5.02)	-2.79 (-2.91 to -2.67)	6.46 (5.00 to 8.24)	1.96 (1.46 to 2.63)	-4.23 (-4.42 to -4.04)	320.22 (247.72 to 408.26)	98.44 (73.11 to 132.17)	-4.17 (-4.36 to -3.99)
Thyroid cancer	1.53 (1.06 to 2.01)	2.65 (1.85 to 4.18)	1.79 (1.61 to 1.97)	0.14 (0.10 to 0.19)	0.07 (0.05 to 0.10)	-2.99 (-3.16 to -2.82)	8.10 (5.63 to 10.77)	4.68 (3.16 to 7.31)	-2.20 (-2.36 to -2.03)
Tracheal, bronchus, and lung cancer	4.74 (3.69 to 5.98)	4.78 (3.58 to 6.31)	-0.35 (-0.47 to -0.24)	4.24 (3.31 to 5.33)	3.40 (2.54 to 4.48)	-1.17 (-1.33 to -1.01)	206.99 (161.42 to 260.54)	165.20 (123.50 to 217.88)	-1.20 (-1.35 to -1.04)

Uterine cancer	2.90 (1.64 to 3.90)	3.52 (2.36 to 5.01)	0.59 (0.17 to 1.02)	0.86 (0.47 to 1.16)	0.39 (0.27 to 0.55)	-2.90 (-3.29 to -2.51)	43.30 (23.50 to 58.73)	20.87 (13.99 to 29.50)	-2.70 (-3.08 to -2.31)

Table S13: The ASR of incidence, mortality, and DALYs for 29 male early-onset cancers in 1990 and 2021 in China, with AAPC from 1990 to 2021.

Early-onset cancers	Incidence			Mortality			DALYs		
	ASR (95 % UI), 1990	ASR (95 % UI), 2021	AAPC (95% CI)	ASR (95 % UI), 1990	ASR (95 % UI), 2021	AAPC (95% CI)	ASR (95 % UI), 1990	ASR (95 % UI), 2021	AAPC (95% CI)
Bladder cancer	1.31 (0.73 to 1.71)	1.78 (1.30 to 2.39)	0.96 (0.76 to 1.17)	0.49 (0.27 to 0.63)	0.30 (0.22 to 0.41)	-1.84 (-2.07 to -1.61)	24.40 (13.52 to 31.51)	15.84 (11.51 to 21.52)	-1.68 (-1.92 to -1.44)
Brain and central nervous system cancer	2.62 (1.57 to 3.75)	4.40 (2.58 to 6.37)	1.68 (1.58 to 1.79)	2.06 (1.25 to 2.94)	2.14 (1.26 to 3.09)	-0.05 (-0.15 to 0.04)	112.53 (67.93 to 160.45)	117.53 (69.12 to 169.22)	-0.03 (-0.13 to 0.07)
Breast cancer	0.19 (0.12 to 0.27)	0.78 (0.29 to 1.15)	6.40 (5.53 to 7.28)	0.06 (0.04 to 0.09)	0.09 (0.03 to 0.14)	2.65 (1.96 to 3.35)	3.24 (1.92 to 4.53)	5.17 (1.89 to 7.45)	2.88 (2.20 to 3.57)
Colon and rectum cancer	7.06 (5.23 to 8.66)	13.39 (9.98 to 17.59)	2.17 (1.86 to 2.48)	4.62 (3.43 to 5.69)	4.26 (3.18 to 5.62)	-0.43 (-0.66 to -0.19)	234.51 (172.36 to 287.96)	220.59 (164.66 to 289.45)	-0.38 (-0.63 to -0.13)
Esophageal cancer	7.81 (6.23 to 9.65)	3.76 (2.79 to 4.87)	-3.09 (-3.32 to -2.86)	6.98 (5.59 to 8.63)	2.66 (1.98 to 3.47)	-3.87 (-4.15 to -3.59)	327.81 (262.94 to 404.61)	125.56 (93.55 to 163.56)	-3.89 (-4.17 to -3.61)
Eye cancer	0.05 (0.02 to 0.09)	0.07 (0.03 to 0.12)	1.43 (1.26 to 1.60)	0.01 (0.00 to 0.02)	0.01 (0.00 to 0.01)	-1.01 (-1.14 to -0.89)	0.62 (0.27 to 0.93)	0.45 (0.18 to 0.73)	-0.79 (-0.92 to -0.66)
Gallbladder and biliary tract cancer	0.41 (0.25 to 0.54)	0.62 (0.33 to 0.88)	1.64 (1.40 to 1.89)	0.34 (0.21 to 0.46)	0.30 (0.16 to 0.42)	-0.31 (-0.43 to -0.20)	16.69 (10.12 to 22.32)	14.51 (7.96 to 20.46)	-0.28 (-0.40 to -0.16)
Hodgkin lymphoma	0.34 (0.13 to 0.49)	0.22 (0.12 to 0.34)	-1.50 (-1.84 to -1.17)	0.29 (0.11 to 0.41)	0.08 (0.05 to 0.13)	-4.49 (-4.74 to -4.24)	16.13 (6.13 to 23.32)	4.49 (2.63 to 7.32)	-4.47 (-4.73 to -4.20)
Kidney cancer	0.88 (0.70 to 1.08)	2.47 (1.88 to 3.20)	4.44 (4.07 to 4.81)	0.36 (0.29 to 0.45)	0.49 (0.37 to 0.64)	1.73 (1.44 to 2.03)	18.48 (14.85 to 22.65)	25.60 (19.59 to 33.18)	1.82 (1.52 to 2.12)
Larynx cancer	0.57 (0.44 to 0.70)	0.53 (0.39 to 0.70)	-0.26 (-0.35 to -0.18)	0.41 (0.32 to 0.51)	0.21 (0.15 to 0.27)	-2.43 (-2.51 to -2.34)	19.54 (15.05 to 24.20)	10.03 (7.36 to 13.36)	-2.38 (-2.47 to -2.29)
Leukemia	4.45 (2.74 to 5.78)	4.86 (2.88 to 6.68)	0.26 (0.13 to 0.39)	3.95 (2.44 to 5.14)	2.75 (1.67 to 3.74)	-1.36 (-1.46 to -1.25)	231.68 (143.12 to 301.44)	161.58 (97.73 to 219.47)	-1.37 (-1.48 to -1.25)
Lip and oral cavity cancer	0.67 (0.53 to 0.82)	1.29 (0.97 to 1.71)	2.83 (2.50 to 3.16)	0.38 (0.30 to 0.46)	0.38 (0.28 to 0.51)	0.51 (0.29 to 0.73)	18.61 (14.80 to 22.83)	19.20 (14.33 to 25.41)	0.57 (0.35 to 0.79)
Liver cancer	8.57 (6.74 to 10.76)	7.83 (5.77 to 10.52)	-0.77 (-1.15 to -0.38)	7.78 (6.12 to 9.73)	5.73 (4.22 to 7.67)	-1.52 (-1.89 to -1.16)	382.40 (301.33 to 478.76)	282.10 (207.93 to 377.95)	-1.57 (-1.94 to -1.19)
Malignant neoplasm of bone and	0.39 (0.23 to	1.01 (0.57 to	3.65 (3.01 to	0.29 (0.17 to	0.46 (0.27 to	1.71 (1.07 to	17.43 (10.37 to	27.17 (15.68 to	1.66 (1.04 to

articular cartilage	0.71)	1.47)	4.30)	0.53)	0.67)	2.36)	31.51)	39.12)	2.28)
Malignant skin melanoma	0.25 (0.12 to 0.34)	0.56 (0.29 to 0.83)	3.11 (2.72 to 3.50)	0.17 (0.08 to 0.23)	0.14 (0.07 to 0.21)	-0.39 (-0.59 to -0.19)	8.59 (4.06 to 11.69)	7.51 (3.96 to 10.93)	-0.30 (-0.50 to -0.09)
Mesothelioma	0.08 (0.06 to 0.10)	0.09 (0.07 to 0.12)	1.47 (1.07 to 1.87)	0.07 (0.05 to 0.09)	0.08 (0.06 to 0.10)	1.34 (0.94 to 1.73)	3.46 (2.66 to 4.44)	4.02 (3.09 to 5.19)	1.28 (0.90 to 1.66)
Multiple myeloma	0.05 (0.04 to 0.12)	0.30 (0.14 to 0.44)	4.81 (4.29 to 5.34)	0.05 (0.03 to 0.10)	0.18 (0.09 to 0.26)	3.56 (3.01 to 4.11)	2.32 (1.55 to 4.86)	9.33 (4.44 to 13.53)	3.66 (3.11 to 4.22)
Nasopharynx cancer	4.11 (3.24 to 5.19)	5.10 (3.61 to 7.05)	-0.14 (-0.72 to 0.44)	2.83 (2.22 to 3.54)	1.15 (0.84 to 1.52)	-3.89 (-4.32 to -3.45)	143.48 (112.87 to 179.59)	59.86 (43.91 to 79.17)	-3.84 (-4.30 to -3.37)
Neuroblastoma and other peripheral nervous cell tumors	0.01 (0.01 to 0.02)	0.07 (0.05 to 0.10)	6.46 (6.02 to 6.90)	0.01 (0.01 to 0.01)	0.04 (0.03 to 0.05)	5.40 (4.97 to 5.83)	0.54 (0.40 to 0.72)	1.97 (1.42 to 2.59)	5.35 (4.92 to 5.77)
Non-Hodgkin lymphoma	1.79 (1.41 to 2.40)	3.75 (2.68 to 4.97)	2.77 (2.39 to 3.14)	1.22 (0.96 to 1.63)	1.10 (0.79 to 1.45)	-0.17 (-0.43 to 0.09)	65.84 (52.02 to 87.73)	60.99 (43.78 to 80.37)	-0.12 (-0.38 to 0.14)
Non-melanoma skin cancer	2.15 (1.36 to 3.20)	10.15 (6.56 to 14.84)	2.80 (2.05 to 3.56)	0.13 (0.09 to 0.18)	0.13 (0.08 to 0.19)	0.33 (0.16 to 0.50)	6.72 (4.68 to 9.33)	7.01 (4.45 to 9.68)	0.35 (0.19 to 0.52)
Other pharynx cancer	0.26 (0.20 to 0.34)	0.28 (0.20 to 0.36)	-0.46 (-1.05 to 0.14)	0.19 (0.15 to 0.25)	0.10 (0.07 to 0.13)	-3.13 (-3.65 to -2.60)	9.11 (7.01 to 11.63)	4.64 (3.40 to 6.07)	-3.08 (-3.61 to -2.55)
Pancreatic cancer	1.55 (1.24 to 1.90)	1.83 (1.38 to 2.39)	0.51 (0.37 to 0.65)	1.41 (1.13 to 1.74)	1.58 (1.19 to 2.06)	0.32 (0.20 to 0.44)	68.46 (54.94 to 84.18)	76.80 (57.79 to 99.85)	0.31 (0.17 to 0.44)
Prostate cancer	0.24 (0.11 to 0.34)	0.55 (0.36 to 0.78)	2.84 (2.66 to 3.03)	0.12 (0.05 to 0.16)	0.09 (0.06 to 0.12)	-1.28 (-1.54 to -1.01)	5.90 (2.64 to 8.32)	4.72 (3.02 to 6.59)	-1.05 (-1.34 to -0.77)
Soft tissue and other extraosseous sarcomas	0.27 (0.16 to 0.43)	0.26 (0.16 to 0.45)	-0.33 (-0.43 to -0.22)	0.15 (0.09 to 0.24)	0.09 (0.06 to 0.16)	-1.88 (-2.02 to -1.74)	8.06 (4.77 to 12.77)	4.93 (3.03 to 8.62)	-1.89 (-2.04 to -1.74)
Stomach cancer	17.10 (12.08 to 21.10)	10.22 (7.30 to 13.82)	-1.83 (-1.93 to -1.72)	13.29 (9.35 to 16.44)	5.28 (3.81 to 7.20)	-3.22 (-3.37 to -3.07)	639.33 (450.19 to 790.19)	258.17 (185.90 to 350.56)	-3.18 (-3.34 to -3.03)
Testicular cancer	0.41 (0.33 to 0.51)	1.19 (0.88 to 1.59)	3.11 (2.74 to 3.49)	0.20 (0.16 to 0.25)	0.12 (0.09 to 0.16)	-2.53 (-2.92 to -2.13)	11.82 (9.46 to 14.56)	7.64 (5.78 to 9.74)	-2.28 (-2.70 to -1.87)
Thyroid cancer	0.41 (0.31 to 0.55)	1.78 (1.26 to 2.40)	5.99 (5.58 to 6.41)	0.08 (0.06 to 0.10)	0.10 (0.07 to 0.13)	1.33 (1.09 to 1.58)	4.15 (3.17 to 5.49)	5.82 (3.98 to 7.84)	1.71 (1.47 to 1.96)
Tracheal, bronchus, and lung cancer	8.39 (6.61 to 10.33)	7.62 (5.58 to 10.09)	-0.40 (-0.51 to -0.28)	7.66 (6.03 to 9.44)	6.17 (4.48 to 8.20)	-0.82 (-0.95 to -0.68)	368.35 (290.55 to 454.18)	297.73 (216.73 to 394.21)	-0.84 (-0.98 to -0.71)

Table S14: Percentage contribution of environmental and occupational risks to early-onset cancers DALYs in China by sex, 2021.

Risk factors	Kidney cancer			Larynx cancer			Leukemia			Mesothelioma			Nasopharynx cancer			Ovarian cancer	Tracheal, bronchus, and lung cancer		
	Both	Female	Male	Both	Female	Male	Both	Female	Male	Both	Female	Male	Both	Female	Male	Female	Both	Female	Male
All risk factors, % (95% UI)	21.20 (11.97 to 30.49)	15.45 (6.16 to 24.91)	22.58 (13.54 to 31.86)	72.74 (66.89 to 77.89)	18.63 (13.22 to 24.71)	82.40 (78.54 to 85.58)	11.96 (9.61 to 14.53)	10.52 (8.04 to 12.94)	12.81 (10.00 to 15.72)	43.24 (30.52 to 54.93)	31.11 (13.73 to 47.71)	48.11 (32.18 to 63.16)	42.55 (35.61 to 49.75)	8.94 (6.25 to 12.35)	51.76 (44.23 to 59.39)	6.05 (1.13 to 11.72)	69.75 (63.13 to 75.57)	50.14 (40.77 to 59.66)	80.09 (76.18 to 83.83)
Ambient particulate matter pollution, % (95% UI)	-	-	-	-	-	-	-	-	-	-	-	-	-	-	-	-	20.87 (12.56 to 29.07)	**20.51 (11.66 to 28.77)**	21.06 (13.00 to 29.22)
Household air pollution from solid fuels, % (95% UI)	-	-	-	-	-	-	-	-	-	-	-	-	-	-	-	-	4.25 (0.63 to 14.02)	5.06 (0.75 to 16.03)	3.83 (0.58 to 12.97)
Residential radon, % (95% UI)	-	-	-	-	-	-	-	-	-	-	-	-	-	-	-	-	3.71 (-1.78 to 9.92)	3.75 (-1.75 to 10.00)	3.69 (-1.79 to 9.84)
Occupational exposure to arsenic, % (95% UI)	-	-	-	-	-	-	-	-	-	-	-	-	-	-	-	-	1.04 (0.39 to 1.69)	1.18 (0.44 to 1.97)	0.97 (0.36 to 1.55)
Occupational exposure to asbestos, % (95% UI)	-	-	-	0.22 (0.09 to 0.39)	0.07 (0.01 to 0.15)	0.24 (0.10 to 0.46)	-	-	-	**43.24 (30.52 to 54.93)**	**31.11 (13.73 to 47.71)**	**48.11 (32.18 to 63.16)**	-	-	-	0.12 (0.03 to 0.28)	0.48 (0.24 to 0.81)	0.15 (0.04 to 0.31)	0.66 (0.30 to 1.17)
Occupational exposure to benzene, % (95% UI)	-	-	-	-	-	-	2.00 (0.59 to 3.38)	2.64 (0.76 to 4.37)	1.62 (0.48 to 2.66)	-	-	-	-	-	-	-	-	-	-

Occupational exposure to beryllium, % (95% UI)	-	-	-	-	-	-	-	-	-	-	-	-	-	-	-	-	0.04 (0.03 to 0.05)	0.04 (0.03 to 0.06)	0.03 (0.02 to 0.05)
Occupational exposure to cadmium, % (95% UI)	-	-	-	-	-	-	-	-	-	-	-	-	-	-	-	-	0.10 (0.07 to 0.13)	0.12 (0.08 to 0.16)	0.09 (0.06 to 0.13)
Occupational exposure to chromium, % (95% UI)	-	-	-	-	-	-	-	-	-	-	-	-	-	-	-	-	0.22 (0.18 to 0.26)	0.25 (0.19 to 0.32)	0.20 (0.15 to 0.25)
Occupational exposure to diesel engine exhaust, % (95% UI)	-	-	-	-	-	-	-	-	-	-	-	-	-	-	-	-	2.29 (1.90 to 2.75)	2.41 (1.82 to 3.14)	2.22 (1.70 to 2.80)
Occupational exposure to formaldehyde, % (95% UI)	-	-	-	-	-	-	0.98 (0.79 to 1.20)	1.11 (0.76 to 1.48)	0.90 (0.65 to 1.17)	-	-	-	2.54 (1.44 to 3.83)	2.95 (1.33 to 4.98)	2.43 (1.09 to 4.15)	-	-	-	-
Occupational exposure to nickel, % (95% UI)	-	-	-	-	-	-	-	-	-	-	-	-	-	-	-	-	1.00 (0.21 to 2.20)	1.08 (0.24 to 2.39)	0.95 (0.20 to 2.08)
Occupational exposure to polycyclic aromatic hydrocarbons, % (95% UI)	-	-	-	-	-	-	-	-	-	-	-	-	-	-	-	-	0.73 (0.57 to 0.92)	0.86 (0.58 to 1.15)	0.67 (0.44 to 0.89)
Occupational exposure to silica, % (95% UI)	-	-	-	-	-	-	-	-	-	-	-	-	-	-	-	-	4.61 (2.16 to	4.54 (2.13 to 7.08)	4.64 (2.17 to 7.31)

																	7.27)		
Occupational exposure to sulfuric acid, % (95% UI)	-	-	-	6.24 (2.64 to 11.47)	6.73 (2.87 to 12.21)	6.16 (2.57 to 11.33)		-	-	-	-	-	-	-	-	-	-	-	-

All risk factors include environmental, occupational behavioral and metabolic risk. Bolding in the table indicates the risk factors with the highest contribution percentage.

Table S15: Percentage contribution of behavioral and metabolic risks to early-onset cancers DALYs in China by sex, 2021.

Cancer	Sex	Alcohol use	Drug use	Chewing tobacco	Secondhand smoke	Smoking	Low physical activity	Unsafe sex	Diet high in processed meat	Diet high in red meat	Diet high in sodium	Diet low in calcium	Diet low in fiber	Diet low in fruits	Diet low in milk	Diet low in vegetables	Diet low in whole grains	High body-mass index	High fasting plasma glucose
Bladder cancer	Both	-	-	-	-	**27.10 (23.28 to 31.19)**	-	-	-	-	-	-	-	-	-	-	-	-	2.75 (-0.42 to 6.00)
	Female	-	-	-	-	2.08 (1.39 to 2.98)	-	-	-	-	-	-	-	-	-	-	-	-	**2.20 (-0.30 to 4.84)**
	Male	-	-	-	-	**32.82 (29.06 to 36.61)**	-	-	-	-	-	-	-	-	-	-	-	-	2.87 (-0.44 to 6.34)
Breast cancer	Both	1.40 (0.87 to 2.08)	-	-	2.49 (-0.63 to 5.60)	0.46 (0.30 to 0.69)	1.21 (0.21 to 2.39)	-	-	**13.55 (-0.01 to 28.62)**	-	-	-	-	-	-	-	-	2.38 (-0.70 to 5.69)
	Female	1.14 (0.65 to 1.73)	-	-	2.53 (-0.64 to 5.69)	0.47 (0.30 to 0.70)	1.24 (0.21 to 2.43)	-	-	**13.55 (-0.01 to 28.63)**	-	-	-	-	-	-	-	-	2.44 (-0.72 to 5.82)
	Male	11.56 (8.27 to 14.73)	-	-	0.93 (-0.23 to 2.20)	-	-	-	-	**13.22 (-0.01 to 27.91)**	-	-	-	-	-	-	-	-	-
Cervical cancer	Female	-	-	-	-	3.41 (1.78 to 5.59)	-	**100.00 (100.00 to 100.00)**	-	-	-	-	-	-	-	-	-	-	-
Colon	Both	8.07	-	-	-	6.34 (4.02	1.38 (0.67	-	3.11	15.95	-	6.81	1.14	-	17.20	-	**17.74**	7.66 (3.18 to	3.95 (1.94

and rectum cancer		(6.24 to 9.99)				to 8.58)	to 2.47)		(-0.72 to 6.66)	(-0.01 to 31.46)		(4.84 to 8.86)	(0.49 to 2.02)		(4.70 to 27.94)		**(7.44 to 26.40)**	12.29)	to 6.27)
	Female	0.86 (0.56 to 1.26)	-	-	-	0.47 (0.27 to 0.75)	2.52 (1.11 to 4.52)	-	3.47 (-0.83 to 7.53)	15.88 (-0.01 to 31.10)	-	11.13 (8.30 to 13.89)	1.29 (0.45 to 2.36)	-	**22.44 (6.24 to 35.65)**	-	17.58 (7.34 to 26.24)	7.84 (3.30 to 12.57)	3.40 (1.65 to 5.22)
	Male	10.78 (8.55 to 13.26)	-	-	-	8.55 (5.53 to 11.64)	0.95 (0.34 to 2.22)	-	2.98 (-0.68 to 6.38)	15.98 (-0.01 to 31.54)	-	5.18 (3.56 to 7.13)	1.09 (0.44 to 2.12)	-	15.23 (4.21 to 24.83)	-	**17.80 (7.48 to 26.43)**	7.59 (3.13 to 12.14)	4.16 (2.02 to 6.69)
Esophageal cancer	Both	24.01 (18.46 to 30.01)	-	1.90 (0.91 to 3.33)	-	**32.95 (26.96 to 38.44)**	-	-	-	-	-	-	-	-	-	2.36 (-0.34 to 7.23)	-	-	-
	Female	**2.41 (1.32 to 3.80)**	-	0.79 (0.34 to 1.59)	-	1.85 (1.11 to 2.92)	-	-	-	-	-	-	-	-	-	1.94 (-0.28 to 6.49)	-	-	-
	Male	26.16 (20.07 to 32.49)	-	2.01 (0.96 to 3.59)	-	**36.04 (29.55 to 42.40)**	-	-	-	-	-	-	-	-	-	2.40 (-0.34 to 7.43)	-	-	-
Gallbladder and biliary tract cancer	Both	-	-	-	-	-	-	-	-	-	-	-	-	-	-	-	-	**11.48 (7.96 to 15.66)**	-
	Female	-	-	-	-	-	-	-	-	-	-	-	-	-	-	-	-	**11.79 (8.16 to 16.11)**	-
	Male	-	-	-	-	-	-	-	-	-	-	-	-	-	-	-	-	**11.31 (7.85 to 15.49)**	-
Kidney	Both	-	-	-	-	6.80 (4.25	-	-	-	-	-	-	-	-	-	-	-	**15.36 (5.87**	-

cancer						to 9.36)												**to 25.13)**	
	Female	-	-	-	-	0.25 (0.13 to 0.44)	-	-	-	-	-	-	-	-	-	-	-	**15.11 (5.82 to 24.65)**	-
	Male	-	-	-	-	8.37 (5.41 to 11.46)	-	-	-	-	-	-	-	-	-	-	-	**15.42 (5.89 to 25.26)**	-
Larynx cancer	Both	18.98 (10.65 to 27.19)	-	-	-	**66.33 (59.76 to 71.32)**	-	-	-	-	-	-	-	-	-	-	-	-	-
	Female	1.80 (0.63 to 3.07)	-	-	-	**11.15 (7.64 to 15.69)**	-	-	-	-	-	-	-	-	-	-	-	-	-
	Male	22.05 (12.27 to 30.87)	-	-	-	**76.18 (71.80 to 80.09)**	-	-	-	-	-	-	-	-	-	-	-	-	-
Leukemia	Both	-	-	-	-	2.50 (0.98 to 4.08)	-	-	-	-	-	-	-	-	-	-	-	**7.01 (5.19 to 8.94)**	-
	Female	-	-	-	-	0.16 (0.05 to 0.29)	-	-	-	-	-	-	-	-	-	-	-	**6.93 (5.15 to 8.93)**	-
	Male	-	-	-	-	3.89 (1.58 to 6.20)	-	-	-	-	-	-	-	-	-	-	-	**7.04 (5.23 to 8.92)**	-
Lip and oral cavity cancer	Both	**32.79 (26.57 to 39.01)**	-	2.59 (1.43 to 4.37)	-	28.69 (20.70 to 35.63)	-	-	-	-	-	-	-	-	-	-	-	-	-
	Female	**4.29 (2.67 to 6.34)**	-	3.17 (1.42 to 5.77)	-	1.69 (0.92 to 2.86)	-	-	-	-	-	-	-	-	-	-	-	-	-
	Male	**40.48 (33.09 to 47.54)**	-	2.43 (1.10 to 4.57)	-	35.95 (26.55 to 44.79)	-	-	-	-	-	-	-	-	-	-	-	-	-
Liver	Both	8.96	5.14	-	-	**14.60**	-	-	-	-	-	-	-	-	-	-	-	8.14 (3.26 to	0.78 (0.09

cancer		(5.14 to 14.09)	(3.92 to 6.53)			**(5.12 to 23.50)**												14.04)	to 1.61)
	Female	9.26 (6.85 to 12.16)	**11.81 (7.90 to 15.74)**	-	-	1.10 (0.32 to 2.17)	-	-	-	-	-	-	-	-	-	-	-	8.55 (3.43 to 15.11)	1.66 (0.17 to 3.40)
	Male	8.91 (4.75 to 14.70)	4.12 (3.21 to 5.08)	-	-	**16.67 (5.87 to 26.97)**	-	-	-	-	-	-	-	-	-	-	-	8.07 (3.23 to 13.87)	0.65 (0.07 to 1.30)
Multiple myeloma	Both	-	-	-	-	-	-	-	-	-	-	-	-	-	-	-	-	**5.67 (-1.78 to 14.75)**	-
	Female	-	-	-	-	-	-	-	-	-	-	-	-	-	-	-	-	**5.76 (-1.89 to 14.93)**	-
	Male	-	-	-	-	-	-	-	-	-	-	-	-	-	-	-	-	**5.61 (-1.75 to 14.50)**	-
Nasopharynx cancer	Both	**32.47 (24.61 to 40.01)**	-	-	-	14.20 (10.74 to 17.58)	-	-	-	-	-	-	-	-	-	-	-	-	-
	Female	**5.40 (3.22 to 8.38)**	-	-	-	0.80 (0.51 to 1.23)	-	-	-	-	-	-	-	-	-	-	-	-	-
	Male	**39.89 (30.84 to 48.32)**	-	-	-	17.87 (13.94 to 22.15)	-	-	-	-	-	-	-	-	-	-	-	-	-
Non-Hodgkin lymphoma	Both	-	-	-	-	-	-	-	-	-	-	-	-	-	-	-	-	**3.98 (1.33 to 6.73)**	-
	Female	-	-	-	-	-	-	-	-	-	-	-	-	-	-	-	-	**3.97 (1.32 to 6.78)**	-

	Male	-	-	-	-	-	-	-	-	-	-	-	-	-	-	-	-	**3.99 (1.33 to 6.71)**	-
Other pharynx cancer	Both	35.28 (27.10 to 42.78)	-	-	-	**45.02 (37.27 to 51.74)**	-	-	-	-	-	-	-	-	-	-	-	-	-
	Female	**4.39 (2.56 to 6.76)**	-	-	-	3.96 (2.47 to 5.89)	-	-	-	-	-	-	-	-	-	-	-	-	-
	Male	40.53 (31.36 to 48.89)	-	-	-	**51.97 (44.02 to 59.45)**	-	-	-	-	-	-	-	-	-	-	-	-	-
Ovarian cancer	Female	-	-	-	-	-	-	-	-	-	-	-	-	-	-	-	-	**5.94 (1.06 to 11.62)**	-
Pancreatic cancer	Both	-	-	-	-	**21.10 (18.59 to 23.42)**	-	-	-	-	-	-	-	-	-	-	-	0.39 (-1.05 to 3.20)	13.00 (1.54 to 24.83)
	Female	-	-	-	-	1.94 (1.33 to 2.83)	-	-	-	-	-	-	-	-	-	-	-	0.63 (-1.00 to 3.61)	**11.19 (1.21 to 21.80)**
	Male	-	-	-	-	**26.99 (24.82 to 29.31)**	-	-	-	-	-	-	-	-	-	-	-	0.32 (-1.08 to 3.10)	13.56 (1.63 to 26.29)
Prostate cancer	Male	-	-	-	-	**8.26 (3.83 to 12.54)**	-	-	-	-	-	-1.22 (-2.65 to 0.22)	-	-	-6.65 (-18.94 to 5.24)	-	-	-	-
Stomach cancer	Both	-	-	-	-	**10.13 (8.15 to 12.07)**	-	-	-	-	8.20 (-0.00 to 40.79)	-	-	-	-	-	-	-	-
	Female	-	-	-	-	0.66 (0.45 to 0.93)	-	-	-	-	**8.11 (-0.00 to**	-	-	-	-	-	-	-	-

											**40.42)**								
	Male	-	-	-	-	**13.49 (11.28 to 15.72)**	-	-	-	-	8.23 (-0.00 to 40.95)	-	-	-	-	-	-	-	-
Thyroid cancer	Both	-	-	-	-	-	-	-	-	-	-	-	-	-	-	-	-	**11.61 (8.70 to 14.77)**	-
	Female	-	-	-	-	-	-	-	-	-	-	-	-	-	-	-	-	**11.65 (8.75 to 14.68)**	-
	Male	-	-	-	-	-	-	-	-	-	-	-	-	-	-	-	-	**11.58 (8.68 to 14.75)**	-
Tracheal, bronchus, and lung cancer	Both	-	-	-	7.91 (1.06 to 14.98)	**46.71 (40.97 to 52.61)**	-	-	-	-	-	-	-	3.06 (1.53 to 4.62)	-	-	-	-	1.28 (-0.27 to 2.87)
	Female	-	-	-	12.99 (1.71 to 23.75)	7.56 (5.11 to 10.78)	-	-	-	-	-	-	-	2.94 (1.50 to 4.42)	-	-	-	-	1.11 (-0.23 to 2.54)
	Male	-	-	-	5.23 (0.65 to 10.53)	**67.36 (63.95 to 70.72)**	-	-	-	-	-	-	-	3.13 (1.51 to 4.79)	-	-	-	-	1.37 (-0.28 to 3.14)
Uterine cancer	Female	-	-	-	-	-	-	-	-	-	-	-	-	-	-	-	-	**25.10 (17.83 to 34.12)**	-

Bolding in the table indicates the risk factors with the highest contribution percentage.

Table S16: Age-standardized DALYs rate of average annual percent change in early-onset cancers attributable to risk factors from 1990 to 2021 by sex.

Cancer	Risk factors	Sex	AAPC (95% CI)
Bladder cancer	All risk factors	Both	-1.94 (-2.14 to -1.75)
	All risk factors	Female	-2.66 (-2.97 to -2.35)
	All risk factors	Male	-1.86 (-2.04 to -1.68)
	High fasting plasma glucose	Both	-0.40 (-0.66 to -0.14)
	High fasting plasma glucose	Female	-1.95 (-2.15 to -1.74)
	High fasting plasma glucose	Male	-0.02 (-0.30 to 0.26)
	Smoking	Both	-2.05 (-2.23 to -1.87)
	Smoking	Female	-3.23 (-3.73 to -2.73)
	Smoking	Male	-1.94 (-2.12 to -1.75)
Breast cancer	Alcohol use	Both	-0.19 (-0.48 to 0.11)
	Alcohol use	Female	-0.75 (-0.98 to -0.52)
	Alcohol use	Male	2.58 (1.53 to 3.65)
	All risk factors	Both	-0.51 (-0.69 to -0.32)
	All risk factors	Female	-0.63 (-0.84 to -0.42)
	All risk factors	Male	2.48 (1.34 to 3.63)
	Diet high in red meat	Both	-0.62 (-0.87 to -0.38)
	Diet high in red meat	Female	-0.73 (-1.00 to -0.47)
	Diet high in red meat	Male	2.37 (1.19 to 3.57)
	High fasting plasma glucose	Both	0.54 (0.32 to 0.77)
	High fasting plasma glucose	Female	0.45 (0.20 to 0.69)
	Low physical activity	Both	-0.24 (-0.35 to -0.12)
	Low physical activity	Female	-0.30 (-0.41 to -0.18)
	Secondhand smoke	Both	-1.02 (-1.26 to -0.77)
	Secondhand smoke	Female	-1.10 (-1.34 to -0.85)
	Secondhand smoke	Male	2.54 (1.35 to 3.75)
	Smoking	Both	-0.76 (-0.91 to -0.61)
	Smoking	Female	-0.87 (-1.04 to -0.70)
Cervical cancer	All risk factors	Female	-1.14 (-1.32 to -0.96)
	Smoking	Female	-1.15 (-1.55 to -0.74)

	Unsafe sex	Female	-1.14 (-1.32 to -0.96)
Colon and rectum cancer	All risk factors	Both	-0.84 (-0.96 to -0.72)
	All risk factors	Female	-2.25 (-2.46 to -2.04)
	All risk factors	Male	-0.14 (-0.28 to 0.01)
	Alcohol use	Both	0.07 (-0.15 to 0.29)
	Alcohol use	Female	-1.89 (-2.18 to -1.59)
	Alcohol use	Male	0.16 (-0.06 to 0.37)
	Diet high in processed meat	Both	1.60 (1.40 to 1.80)
	Diet high in processed meat	Female	0.42 (0.24 to 0.59)
	Diet high in processed meat	Male	2.25 (2.01 to 2.49)
	Diet high in red meat	Both	-0.58 (-0.70 to -0.47)
	Diet high in red meat	Female	-1.95 (-2.19 to -1.72)
	Diet high in red meat	Male	0.19 (0.06 to 0.31)
	Diet low in calcium	Both	-3.47 (-3.67 to -3.28)
	Diet low in calcium	Female	-3.62 (-3.83 to -3.41)
	Diet low in calcium	Male	-3.35 (-3.66 to -3.05)
	Diet low in fiber	Both	-3.47 (-3.76 to -3.18)
	Diet low in fiber	Female	-4.72 (-5.06 to -4.38)
	Diet low in fiber	Male	-2.81 (-3.10 to -2.52)
	Diet low in milk	Both	-0.98 (-1.11 to -0.86)
	Diet low in milk	Female	-2.14 (-2.39 to -1.89)
	Diet low in milk	Male	0.02 (-0.11 to 0.16)
	Diet low in whole grains	Both	-0.88 (-1.02 to -0.75)
	Diet low in whole grains	Female	-2.21 (-2.47 to -1.95)
	Diet low in whole grains	Male	-0.12 (-0.24 to 0.01)
	High body-mass index	Both	2.10 (1.99 to 2.21)
	High body-mass index	Female	0.64 (0.46 to 0.83)
	High body-mass index	Male	2.89 (2.75 to 3.03)
	High fasting plasma glucose	Both	0.42 (0.26 to 0.59)
	High fasting plasma glucose	Female	-0.91 (-1.12 to -0.70)

	High fasting plasma glucose	Male	0.88 (0.65 to 1.11)
	Low physical activity	Both	-1.07 (-1.19 to -0.94)
	Low physical activity	Female	-2.00 (-2.15 to -1.84)
	Low physical activity	Male	0.26 (0.09 to 0.43)
	Smoking	Both	-0.81 (-0.92 to -0.69)
	Smoking	Female	-2.19 (-2.45 to -1.92)
	Smoking	Male	-0.68 (-0.80 to -0.57)
Esophageal cancer	All risk factors	Both	-4.21 (-4.79 to -3.63)
	All risk factors	Female	-9.64 (-10.40 to -8.87)
	All risk factors	Male	-3.93 (-4.64 to -3.22)
	Alcohol use	Both	-3.18 (-3.53 to -2.84)
	Alcohol use	Female	-4.95 (-5.36 to -4.54)
	Alcohol use	Male	-3.04 (-3.44 to -2.65)
	Chewing tobacco	Both	-3.05 (-3.54 to -2.55)
	Chewing tobacco	Female	-4.71 (-5.64 to -3.77)
	Chewing tobacco	Male	-2.90 (-3.37 to -2.43)
	Diet low in vegetables	Both	-11.54 (-12.70 to -10.37)
	Diet low in vegetables	Female	-13.79 (-14.99 to -12.57)
	Diet low in vegetables	Male	-11.20 (-12.31 to -10.07)
	Smoking	Both	-4.05 (-4.46 to -3.65)
	Smoking	Female	-5.58 (-6.05 to -5.11)
	Smoking	Male	-3.94 (-4.32 to -3.55)
Gallbladder and biliary tract cancer	All risk factors	Both	0.96 (0.82 to 1.10)
	All risk factors	Female	0.19 (-0.06 to 0.44)
	All risk factors	Male	1.63 (1.46 to 1.80)
	High body-mass index	Both	0.96 (0.82 to 1.10)
	High body-mass index	Female	0.19 (-0.06 to 0.44)
	High body-mass index	Male	1.63 (1.46 to 1.80)
Kidney cancer	All risk factors	Both	2.65 (2.05 to 3.25)
	All risk factors	Female	2.14 (1.35 to 2.93)

	All risk factors	Male	2.82 (2.20 to 3.45)
	High body-mass index	Both	3.56 (2.98 to 4.13)
	High body-mass index	Female	2.19 (1.41 to 2.98)
	High body-mass index	Male	4.03 (3.42 to 4.64)
	Occupational exposure to trichloroethylene	Both	2.02 (1.52 to 2.51)
	Occupational exposure to trichloroethylene	Female	1.35 (0.82 to 1.89)
	Occupational exposure to trichloroethylene	Male	2.24 (1.72 to 2.76)
	Smoking	Both	1.21 (0.70 to 1.72)
	Smoking	Female	0.08 (-0.23 to 0.39)
	Smoking	Male	1.30 (0.80 to 1.81)
Larynx cancer	All risk factors	Both	-2.50 (-2.58 to -2.42)
	All risk factors	Female	-2.50 (-2.69 to -2.32)
	All risk factors	Male	-2.41 (-2.49 to -2.33)
	Alcohol use	Both	-2.02 (-2.21 to -1.82)
	Alcohol use	Female	-2.25 (-2.59 to -1.91)
	Alcohol use	Male	-1.93 (-2.13 to -1.73)
	Occupational exposure to asbestos	Both	-0.61 (-1.68 to 0.47)
	Occupational exposure to asbestos	Female	-2.73 (-3.32 to -2.14)
	Occupational exposure to asbestos	Male	-0.37 (-1.53 to 0.80)
	Occupational exposure to sulfuric acid	Both	-2.18 (-2.42 to -1.94)
	Occupational exposure to sulfuric acid	Female	-2.20 (-2.42 to -1.97)
	Occupational exposure to sulfuric acid	Male	-2.08 (-2.34 to -1.82)
	Smoking	Both	-2.48 (-2.69 to -2.27)
	Smoking	Female	-2.69 (-2.85 to -2.53)
	Smoking	Male	-2.38 (-2.56 to -2.20)
Leukemia	All risk factors	Both	-0.34 (-0.55 to -0.13)
	All risk factors	Female	-0.60 (-0.77 to -0.43)
	All risk factors	Male	-0.22 (-0.38 to -0.05)
	High body-mass index	Both	0.18 (0.01 to 0.35)
	High body-mass index	Female	-0.72 (-0.81 to -0.63)

	High body-mass index	Male	0.72 (0.57 to 0.88)
	Occupational exposure to benzene	Both	-0.32 (-0.51 to -0.14)
	Occupational exposure to benzene	Female	-0.02 (-0.21 to 0.18)
	Occupational exposure to benzene	Male	-0.59 (-0.81 to -0.36)
	Occupational exposure to formaldehyde	Both	-0.24 (-0.41 to -0.08)
	Occupational exposure to formaldehyde	Female	-0.51 (-0.67 to -0.34)
	Occupational exposure to formaldehyde	Male	-0.05 (-0.24 to 0.15)
	Smoking	Both	-1.56 (-1.67 to -1.45)
	Smoking	Female	-2.31 (-2.43 to -2.20)
	Smoking	Male	-1.46 (-1.57 to -1.36)
Lip and oral cavity cancer	All risk factors	Both	0.29 (-0.05 to 0.63)
	All risk factors	Female	-1.57 (-1.84 to -1.30)
	All risk factors	Male	0.50 (0.13 to 0.87)
	Alcohol use	Both	0.56 (0.15 to 0.97)
	Alcohol use	Female	-1.67 (-2.08 to -1.26)
	Alcohol use	Male	0.68 (0.24 to 1.13)
	Chewing tobacco	Both	0.20 (0.01 to 0.39)
	Chewing tobacco	Female	-1.27 (-1.42 to -1.11)
	Chewing tobacco	Male	0.85 (0.59 to 1.11)
	Smoking	Both	0.08 (-0.30 to 0.46)
	Smoking	Female	-1.95 (-2.26 to -1.65)
	Smoking	Male	0.07 (-0.46 to 0.60)
Liver cancer	Alcohol use	Both	-1.19 (-1.70 to -0.68)
	Alcohol use	Female	-2.02 (-2.64 to -1.40)
	Alcohol use	Male	-0.97 (-1.46 to -0.46)
	All risk factors	Both	-0.91 (-1.44 to -0.38)
	All risk factors	Female	-0.92 (-1.55 to -0.27)
	All risk factors	Male	-0.88 (-1.39 to -0.38)
	Drug use	Both	-0.74 (-1.05 to -0.43)
	Drug use	Female	-0.04 (-1.07 to 1.00)

	Drug use	Male	-0.62 (-1.24 to -0.00)
	High body-mass index	Both	3.20 (2.65 to 3.74)
	High body-mass index	Female	2.20 (1.62 to 2.79)
	High body-mass index	Male	3.43 (2.89 to 3.97)
	High fasting plasma glucose	Both	-0.06 (-0.65 to 0.54)
	High fasting plasma glucose	Female	-1.20 (-1.53 to -0.86)
	High fasting plasma glucose	Male	0.34 (0.06 to 0.63)
	Smoking	Both	-1.69 (-2.24 to -1.14)
	Smoking	Female	-2.30 (-2.95 to -1.65)
	Smoking	Male	-1.63 (-2.17 to -1.09)
Mesothelioma	All risk factors	Both	1.38 (0.29 to 2.49)
	All risk factors	Female	0.28 (-0.33 to 0.89)
	All risk factors	Male	1.88 (0.62 to 3.14)
	Occupational exposure to asbestos	Both	1.38 (0.29 to 2.49)
	Occupational exposure to asbestos	Female	0.28 (-0.33 to 0.89)
	Occupational exposure to asbestos	Male	1.88 (0.62 to 3.14)
Multiple myeloma	All risk factors	Both	7.44 (6.88 to 8.00)
	All risk factors	Female	6.58 (6.02 to 7.15)
	All risk factors	Male	8.05 (7.50 to 8.60)
	High body-mass index	Both	7.44 (6.88 to 8.00)
	High body-mass index	Female	6.58 (6.02 to 7.15)
	High body-mass index	Male	8.05 (7.50 to 8.60)
Nasopharynx cancer	All risk factors	Both	-3.16 (-3.50 to -2.81)
	All risk factors	Female	-4.18 (-4.58 to -3.77)
	All risk factors	Male	-3.05 (-3.39 to -2.70)
	Alcohol use	Both	-3.08 (-3.43 to -2.74)
	Alcohol use	Female	-4.42 (-4.83 to -4.00)
	Alcohol use	Male	-2.87 (-3.21 to -2.54)
	Occupational exposure to formaldehyde	Both	-2.44 (-2.90 to -1.98)
	Occupational exposure to formaldehyde	Female	-3.27 (-3.76 to -2.77)

	Occupational exposure to formaldehyde	Male	-2.14 (-2.57 to -1.71)
	Smoking	Both	-3.48 (-3.83 to -3.13)
	Smoking	Female	-4.79 (-5.20 to -4.37)
	Smoking	Male	-3.39 (-3.74 to -3.03)
Non-Hodgkin lymphoma	All risk factors	Both	1.53 (1.26 to 1.80)
	All risk factors	Female	0.34 (0.04 to 0.64)
	All risk factors	Male	2.19 (1.88 to 2.50)
	High body-mass index	Both	1.53 (1.26 to 1.80)
	High body-mass index	Female	0.34 (0.04 to 0.64)
	High body-mass index	Male	2.19 (1.88 to 2.50)
Other pharynx cancer	All risk factors	Both	-2.81 (-3.25 to -2.37)
	All risk factors	Female	-1.94 (-2.28 to -1.59)
	All risk factors	Male	-2.75 (-3.21 to -2.28)
	Alcohol use	Both	-2.53 (-2.96 to -2.11)
	Alcohol use	Female	-1.73 (-2.13 to -1.33)
	Alcohol use	Male	-2.47 (-2.91 to -2.02)
	Smoking	Both	-3.04 (-3.50 to -2.59)
	Smoking	Female	-2.13 (-2.31 to -1.96)
	Smoking	Male	-2.96 (-3.43 to -2.49)
Ovarian cancer	All risk factors	Female	5.05 (4.87 to 5.23)
	High body-mass index	Female	5.47 (5.19 to 5.74)
	Occupational exposure to asbestos	Female	-1.11 (-1.66 to -0.56)
Pancreatic cancer	All risk factors	Both	0.41 (0.31 to 0.52)
	All risk factors	Female	0.86 (0.63 to 1.09)
	All risk factors	Male	0.47 (0.36 to 0.57)
	High fasting plasma glucose	Both	1.10 (0.88 to 1.32)
	High fasting plasma glucose	Female	0.35 (0.10 to 0.60)
	High fasting plasma glucose	Male	1.46 (1.25 to 1.66)
	Smoking	Both	-0.18 (-0.27 to -0.10)
	Smoking	Female	-0.97 (-1.31 to -0.63)

	Smoking	Male	-0.07 (-0.15 to 0.02)
Prostate cancer	All risk factors	Male	-1.55 (-3.28 to 0.21)
	Smoking	Male	-1.71 (-1.95 to -1.47)
Stomach cancer	All risk factors	Both	-3.60 (-3.86 to -3.35)
	All risk factors	Female	-4.05 (-4.35 to -3.75)
	All risk factors	Male	-3.46 (-3.69 to -3.24)
	Diet high in sodium	Both	-3.48 (-3.63 to -3.33)
	Diet high in sodium	Female	-4.05 (-4.36 to -3.74)
	Diet high in sodium	Male	-3.14 (-3.41 to -2.86)
	Smoking	Both	-3.78 (-4.00 to -3.56)
	Smoking	Female	-3.94 (-4.19 to -3.69)
	Smoking	Male	-3.68 (-3.88 to -3.47)
Thyroid cancer	All risk factors	Both	1.03 (0.83 to 1.22)
	All risk factors	Female	-0.70 (-0.93 to -0.47)
	All risk factors	Male	3.07 (2.53 to 3.61)
	High body-mass index	Both	1.03 (0.83 to 1.22)
	High body-mass index	Female	-0.70 (-0.93 to -0.47)
	High body-mass index	Male	3.07 (2.53 to 3.61)
Tracheal bronchus and lung cancer	All risk factors	Both	-1.22 (-1.35 to -1.09)
	All risk factors	Female	-1.70 (-1.87 to -1.53)
	All risk factors	Male	-0.96 (-1.10 to -0.82)
	Ambient particulate matter pollution	Both	2.25 (2.07 to 2.44)
	Ambient particulate matter pollution	Female	2.82 (2.68 to 2.97)
	Ambient particulate matter pollution	Male	2.04 (1.82 to 2.25)
	Diet low in fruits	Both	-4.37 (-4.85 to -3.90)
	Diet low in fruits	Female	-4.58 (-5.13 to -4.03)
	Diet low in fruits	Male	-4.31 (-4.75 to -3.88)
	High fasting plasma glucose	Both	0.44 (0.25 to 0.64)
	High fasting plasma glucose	Female	0.36 (0.10 to 0.62)
	High fasting plasma glucose	Male	0.55 (0.35 to 0.74)

	Household air pollution from solid fuels	Both	-7.30 (-7.65 to -6.94)
	Household air pollution from solid fuels	Female	-7.27 (-7.64 to -6.90)
	Household air pollution from solid fuels	Male	-7.28 (-7.65 to -6.92)
	Occupational exposure to arsenic	Both	-0.03 (-0.20 to 0.14)
	Occupational exposure to arsenic	Female	0.13 (-0.10 to 0.37)
	Occupational exposure to arsenic	Male	-0.09 (-0.24 to 0.06)
	Occupational exposure to asbestos	Both	0.52 (-0.63 to 1.69)
	Occupational exposure to asbestos	Female	-1.25 (-1.79 to -0.70)
	Occupational exposure to asbestos	Male	0.84 (-0.41 to 2.11)
	Occupational exposure to beryllium	Both	-0.91 (-1.14 to -0.67)
	Occupational exposure to beryllium	Female	-0.63 (-0.96 to -0.29)
	Occupational exposure to beryllium	Male	-1.02 (-1.21 to -0.83)
	Occupational exposure to cadmium	Both	-0.06 (-0.23 to 0.10)
	Occupational exposure to cadmium	Female	0.15 (-0.10 to 0.39)
	Occupational exposure to cadmium	Male	-0.12 (-0.28 to 0.03)
	Occupational exposure to chromium	Both	0.33 (0.17 to 0.49)
	Occupational exposure to chromium	Female	0.64 (0.42 to 0.86)
	Occupational exposure to chromium	Male	0.20 (0.06 to 0.34)
	Occupational exposure to diesel engine exhaust	Both	0.16 (-0.04 to 0.36)
	Occupational exposure to diesel engine exhaust	Female	0.65 (0.38 to 0.92)
	Occupational exposure to diesel engine exhaust	Male	-0.04 (-0.21 to 0.12)
	Occupational exposure to nickel	Both	-0.44 (-0.63 to -0.26)
	Occupational exposure to nickel	Female	-0.27 (-0.53 to -0.01)
	Occupational exposure to nickel	Male	-0.47 (-0.64 to -0.31)
	Occupational exposure to polycyclic aromatic hydrocarbons	Both	0.29 (0.13 to 0.45)
	Occupational exposure to polycyclic aromatic hydrocarbons	Female	0.64 (0.41 to 0.88)
	Occupational exposure to polycyclic aromatic hydrocarbons	Male	0.13 (-0.02 to 0.27)
	Occupational exposure to silica	Both	-0.58 (-0.78 to -0.38)
	Occupational exposure to silica	Female	-0.61 (-0.88 to -0.35)
	Occupational exposure to silica	Male	-0.53 (-0.71 to -0.35)

	Residential radon	Both	-0.91 (-1.08 to -0.74)
	Residential radon	Female	-0.99 (-1.26 to -0.72)
	Residential radon	Male	-0.82 (-0.95 to -0.69)
	Secondhand smoke	Both	-0.95 (-1.13 to -0.78)
	Secondhand smoke	Female	-1.24 (-1.55 to -0.93)
	Secondhand smoke	Male	-0.65 (-0.78 to -0.51)
	Smoking	Both	-0.96 (-1.09 to -0.83)
	Smoking	Female	-0.96 (-1.15 to -0.76)
	Smoking	Male	-0.86 (-1.00 to -0.72)
Uterine cancer	All risk factors	Female	0.03 (-0.38 to 0.44)
	High body-mass index	Female	0.03 (-0.38 to 0.44)

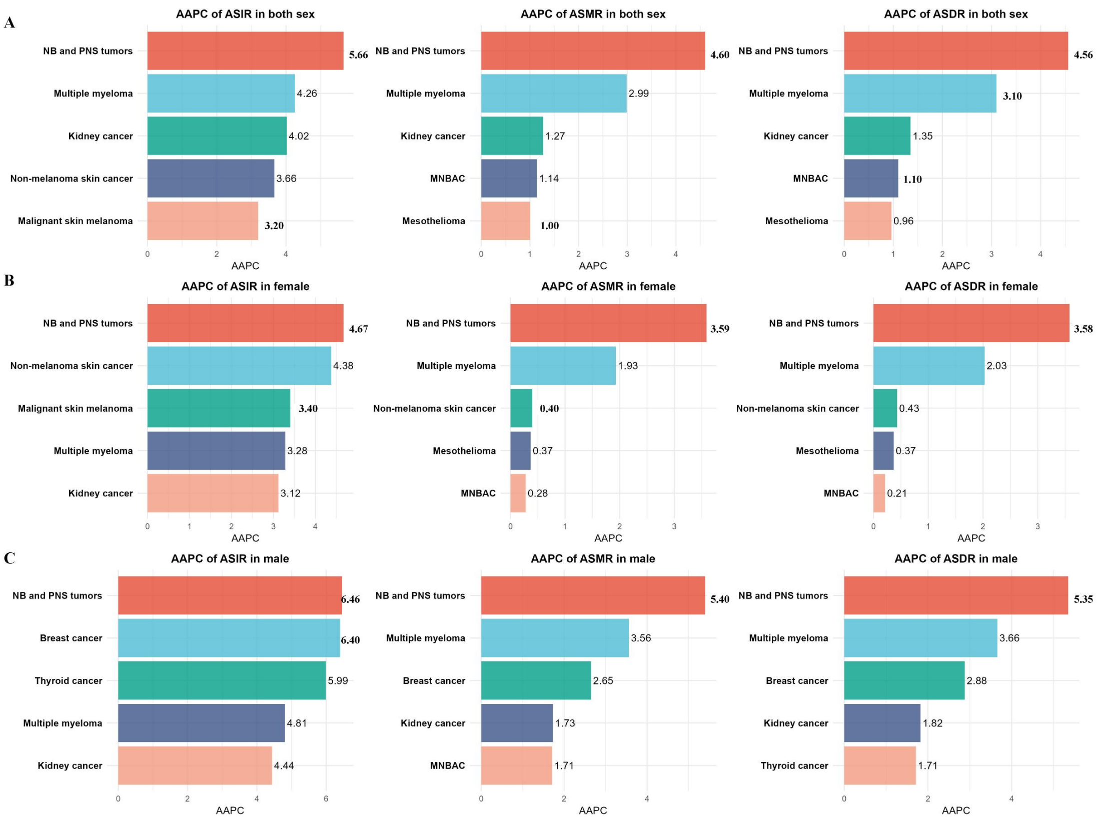

Figure S1: Top five cancers with the highest increase in ASIR, ASMR and ASDR by sex in China from 1990 - 2021.

Panel A. AAPC in both sexes. Panel B. AAPC in both female. Panel C. AAPC in both male. AAPC – average annual percent change, ASDR – age-standardised disability-adjusted life years rate, ASIR – age-standardised incidence rate, ASMR – age-standardised mortality rate.

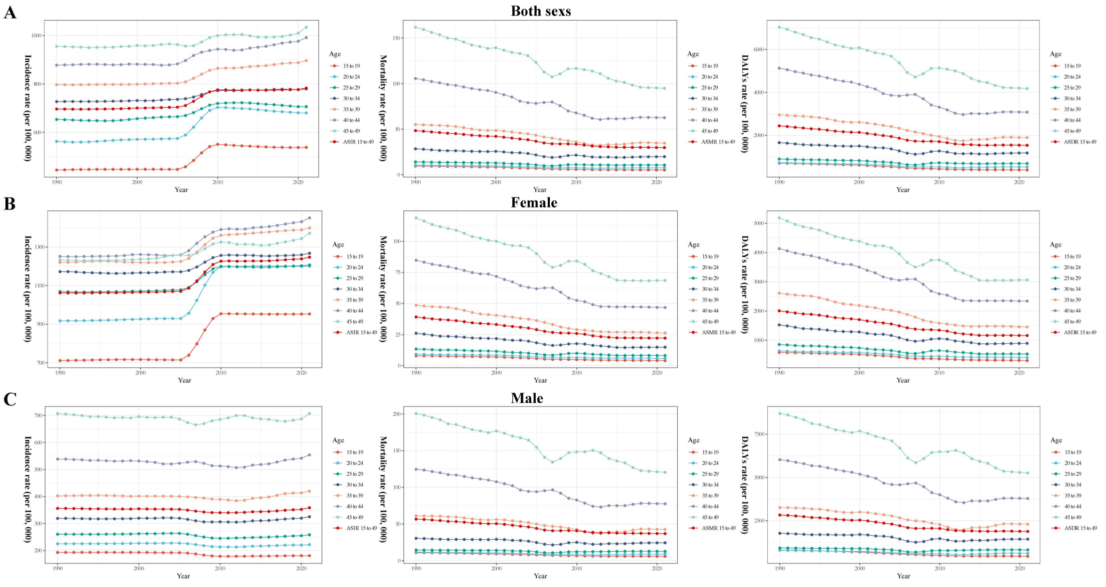

Figure S2: Trends in incidence, mortality and DALYs rates for early-onset cancers in China by age group from 1990 to 2021 by sex.

DALYs – disability-adjusted life years.
